# Tuning Heptazine-Based g‑C_3_N_4_ Structures for Photocatalysis by Enhancing Chemical Stability and Electron–Hole Pair Separation: A Computational Study

**DOI:** 10.1021/acsomega.5c07366

**Published:** 2026-01-21

**Authors:** Leticia C.S. Faria, Aditya N. Raju, Julio C.V. Chagas, Adelia J.A. Aquino, Reed Nieman, Francisco B.C. Machado, Leonardo T. Ueno, Hans Lischka, Luiz F. A. Ferrão

**Affiliations:** † Department of Chemistry, Aeronautics Institute of Technology, São José dos Campos 12228-900, Brazil; ‡ Advanced Scientific Computing and Modeling Laboratory, Aeronautics Institute of Technology, São José dos Campos 12228-900, Brazil; § Department of Chemistry and Biochemistry, 6177 Texas Tech University, Lubbock, Texas 79409, United States; ∥ Department of Chemistry, Northwestern University, Evanston, Illinois 60208, United States; ⊥ Department of Mechanical Engineering, 6177 Texas Tech University, Lubbock, Texas 79409, United States

## Abstract

Due to its charge-transfer capabilities and tunable band structure, graphitic carbon nitride (g-C_3_N_4_) stands out as a promising photocatalyst. However, its efficiency is limited by low visible-light absorption and the rapid recombination of electron–hole pairs. This computational study uses density functional theory (DFT) to investigate the influence of BH and NH substitution on g-C_3_N_4_ building blocks, which can be combined to promote charge transfer and visible-light absorption. The introduction of boron (BH substitution) creates an electron-deficient region and enhances charge transfer, thereby improving the photocatalytic efficiency, while hydrogen (NH substitution) adjusts the excitation energy levels, shifting them into the visible spectrum and placing them in the correct energetic alignment with respect to the standard hydrogen electrode (SHE) and oxygen evolution reaction (OER) potentials. The results demonstrate the interesting potential of combining different substitution strategies within a single photocatalyst model without compromising the individual physical properties of each substitution type, thereby enhancing light absorption and reducing the electron–hole recombination rate.

## Graphical abstract

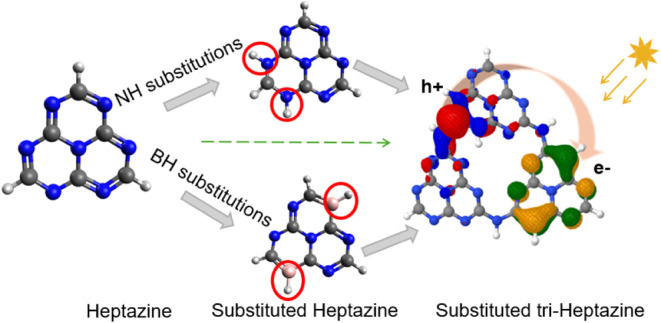

## Introduction

The progress of global industrial development has led to numerous challenges, among which climate changes are top-ranked,^1^ emphasizing the urgent need for the development of sustainable energy resources.^2^ In this context, research on electrocatalytic^3^ and photocatalytic^4^ water splitting has received considerable attention.^5^ Among the available approaches, photocatalysis stands out for using inexhaustible solar energy to produce fuels, making it both safe and environmentally friendly.^6,7^ However, the development of materials that meet photocatalytic criteria, whether they are biologically inspired, environmentally sustainable, or efficient under solar irradiation, remains a significant challenge.^8,9^ The graphitic carbon nitride semiconductor material, g-C_3_N_4_, shows significant advantages such as a large interfacial contact area,^10^ excellent charge transfer (CT) capability,^11^ and easily tunable band gaps.^12,13^ This material contains basic building blocks such as triazine rings and tri-s-triazine/heptazine rings, which can be extended into various 2D structure sizes.^14,15^

The general features of a semiconductor photocatalyst in terms of its energy band scheme are listed in Figure 1. Upon photon excitation, pairs of an electron located in the conduction band (CB) and a hole in the valence band (VB) are created.^16,17^ The photogenerated electrons and holes that reach the particle surfaces will participate in redox reactions involving the target molecules of the oxygen evolution reaction (OER) and the hydrogen evolution reaction (HER)^18,19^ (Figure 1). Therefore, it is critical that this energy scheme is aligned with appropriate molecular energy levels to meet simultaneously the criteria for light absorption and transfer of charge carriers.^20,21^

**Figure 1:**
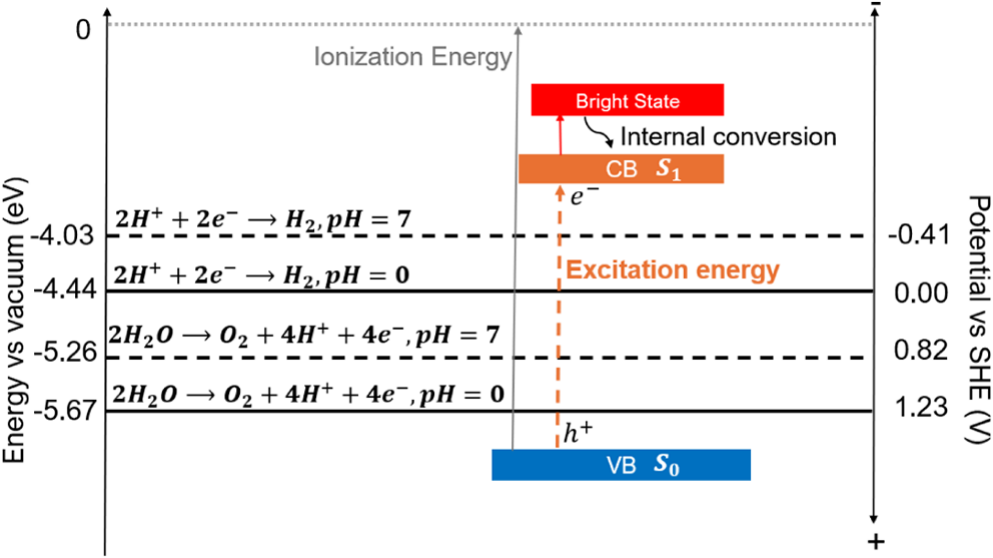
Absolute energy scale versus vacuum (left) is shown in comparison to the potentials vs HER and OER reactions (right scale). The conduction band (CB) is shown in orange, the valence band (VB) is shown in blue, and the level of the bright state is shown in red. The ionization is shown to position the energy levels for the molecular systems (work function for solids).

To relate the thermodynamic properties of the HER and OER reactions to molecular energy levels,^18,22^ they are shown in Figure 1 relative to the vacuum as well as to the standard hydrogen electrode (SHE) potential, based on the vacuum value of −4.44 eV for the latter.^23,24^ The thermodynamic criteria for the OER and the HER are essential to ensure the minimum requirements for water electrolysis and maximum efficiency. The theoretical potential of 1.23 V for the OER at pH 0 at 25 °C defines the thermodynamic limit. Efficient catalysts should operate close to the equilibrium potential, with the OER at +1.23 V and HER at 0.00 V versus SHE at pH 0. These values correspond to −5.67 eV (OER) and −4.44 eV (HER) on the energy vs vacuum scale (eV). At pH 7, these values shift to 0.82 V for OER and −0.41 V for HER vs SHE, corresponding to −5.26 eV (OER) and −4.03 eV (HER).^18,25^

To introduce molecular energy levels into the scheme of Figure 1, for the valence band of a molecular catalyst, its ionization energy is adopted motivated by Koopmans’ theorem.^26^ The energy level of the conduction band is represented by the first excited state level with respect to the vacuum, given by the ionization potential minus the excitation energy. The bright state, shown in red in Figure 1, characterizes the lowest electronic state with a significant oscillator strength, indicating the energy levels at which the structure will absorb light most effectively. According to Kasha’s rule,^27^ upon excitation to the bright state, electronic relaxation to S_1_ will occur via internal conversion in a process, which is usually much faster than fluorescence.^27,28^ Therefore, it is critical to evaluate both the bright state to determine absorption efficiency and the first excited singlet state to determine long-lived exciton and CT states, which are characterized by a nonlocal displacement of charge between distinct regions of a molecule. Identifying these regions allows one to highlight cases in which charge recombination (of the generated electron and hole) is slow. In this work, we adopt the definition of CT as the transfer of charge between distinct heptazine fragments. The comparison of the S_0_ and S_1_ energy levels to the OER and HER potentials will henceforth be referred to as the thermodynamic criteria (for water splitting).

The electronic structure of g-C_3_N_4_ is characterized by the lone pairs of electrons present on the nitrogen atoms that are part of the heptazine units.^19^ These nanostructures with their conjugated π system exhibit a band gap of 2.7 eV in their polymeric conformers.^29,30^ Such a band gap allows absorption of visible light up to about 460 nm; however, the absorption coefficient in this region remains relatively low, resulting in poor light-harvesting efficiency. Moreover, the photogenerated charge carriers tend to undergo rapid electron–hole recombination, which severely limits the overall photocatalytic activity.^31^ In 2016, Liu^32^ stated that the quantum efficiency of g-C_3_N_4_ under light irradiation with a wavelength range of 420–460 nm is approximately 0.1%,^32^ highlighting the intrinsic limitations of its electronic structure and the need for structural or compositional modifications to improve its performance.

To overcome these efficiency barriers and tailor the energy levels, several modifications, substitution strategies, and heterojunctions have been proposed.^33–36^ According to Lin et al.,^21^ to achieve improved photocatalytic performance, particularly for efficient water splitting, a material must exhibit enhanced visible-light absorption, rapid photoinduced separation of electrons and holes, efficient charge migration, and an increased number of unsaturated active sites.^21^ The incorporation of borohydride (BH) into g-C_3_N_4_-based structures can facilitate the CT of electrons from the π-electron system into the unoccupied 2p orbital of B. In addition, the electron-deficient nature of boron improves the carrier migration of the π electrons.^20^ Furthermore, the presence of electron-donating properties of edge-graphitic NH defects^37^ introduced into g-C_3_N_4_, especially when combined with boron defects, should induce the formation of CT states within the π-conjugated lattice.

Considering the many available structures of g-C_3_N_4_ reported in the literature^30^ and their current capabilities as photocatalysts, our work focuses on one of the most popular cases, the heptazine. For comparison, structures of triazine-based (single-ring) and heptazine (HZ)-based (three-ring) compounds (Figure 2a) are shown in Figure 2b. Experimentally, two pathways have been proposed for thermal decomposition from melamine (1). The first involves the direct formation of melem (7) in the initial condensation step, while the second suggests the intermediate formation of melam (2) prior to melem. Further loss of ammonia can lead to melem-based polymers (6), ultimately yielding graphitic carbon nitride (8).^28,36,38,39^ These different molecular structures depicted in Figure 2b provide a basis for studying their photocatalytic properties.

**Figure 2:**
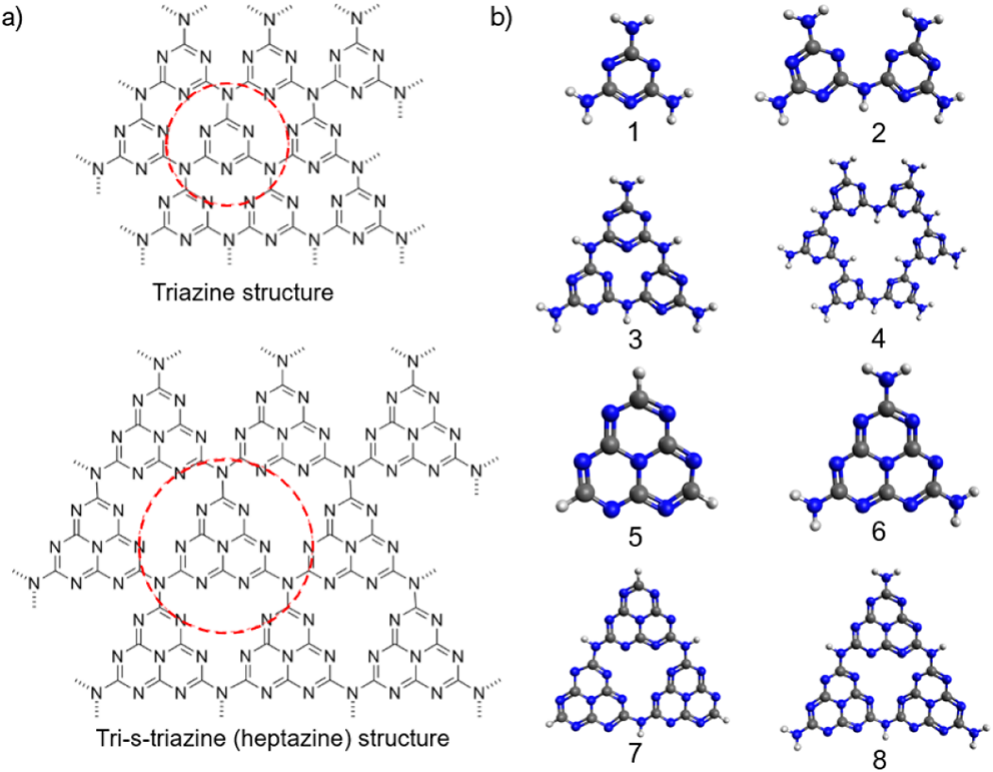
(a) Building blocks of graphitic carbon nitride (g-C_3_N_4_), showing triazine (single ring) and heptazine (three rings) units encircled. (b) Commonly reported g-C_3_N_4_ components, including melamine (1), melam (2), trimelamine (3), poly triazine-imide (PTI) (4), heptazine (5), melem (6), triheptazine (7), and melon (8), leading to the synthesis of g-C_3_N_4_.

The present study is organized as follows: first, the electronic structure of g-C_3_N_4_ precursors is analyzed. Then, substitution strategies for heptazine are proposed to tailor its properties and enhance its photocatalytic potential. Two different substitution approaches are considered (Figure 3): electron-accepting boron–hydrogen (BH) and electron-donating nitrogen–hydrogen (NH) (edge graphitic nitrogen).^37^ BH substitution introduces an electron hole into the π-conjugated system. Graphitic NH substitution is achieved by attaching a hydrogen atom to the pyridinic N atom of the heptazine. This process moves an electron from the initial lone pair to the π orbital of N, so that N now adds two electrons to the conjugated π system.^40^ After studying these substituted heptazine structures separately, a connection of the two substituted fragment types to combine their effects on the electronic structure is proposed. A third pristine fragment was also connected to the other two fragments, forming triheptazine (7) substituted structures. These substituted triheptazines (THZ) were used as a model for a g-C_3_N_4_ catalyst.

**Figure 3:**
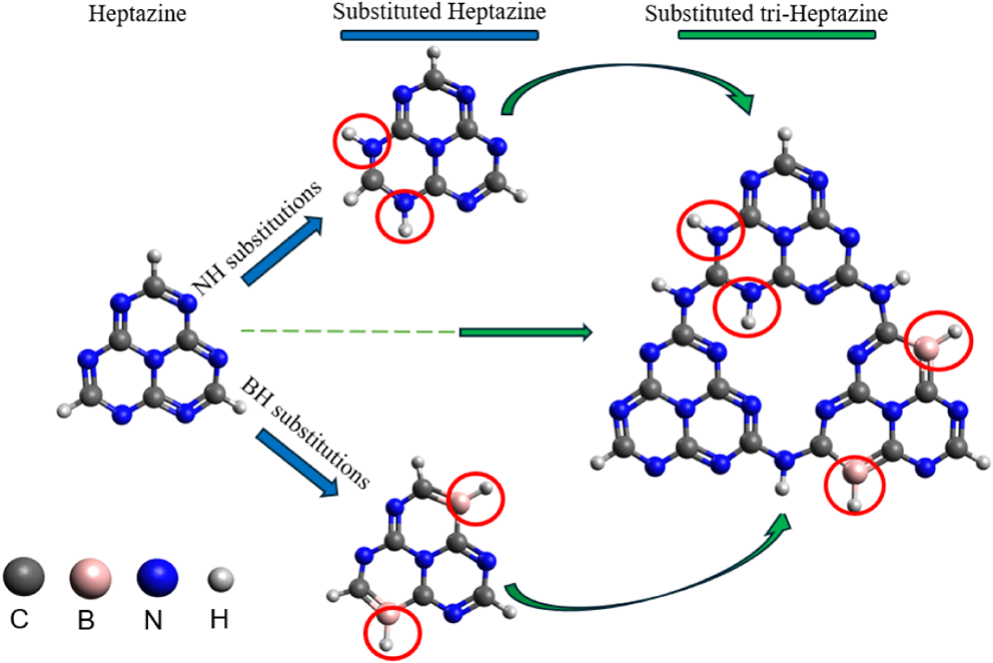
Schematic representation of the substitution schemes and the structure of the final THZ photocatalyst candidate.

The analysis of these structures focuses on thermodynamic criteria^41^ (Figure 1). CT properties are evaluated by analyzing the electron–hole creation in different spatial regions^42^ of the THZ structure, with the aim of reducing the recombination process. Photon absorption is discussed by calculating the spectrum in the visible region.

## Methodology

The geometry optimizations and vibrational frequency analyses of all structures in the ground state were performed using density functional theory (DFT) with the ωB97X-D^43–45^ functional and the def2-SV­(P)^46^ basis set, without any geometric constraints. Vertical excitations were calculated using time-dependent DFT (TD-DFT),^47,48^ with the same functional and basis set as described above. The ωB97X-D functional was employed for all DFT and TD-DFT calculations due to its long-range corrected hybrid formulation, which effectively describes both local and charge-transfer excitations in conjugated organic frameworks. This functional combines 100% Hartree–Fock exchange at long range with a generalized gradient approximation (GGA) at short range, along with an empirical dispersion correction, and usually provides an accurate treatment of noncovalent and delocalized electronic interactions. Studies have demonstrated that ωB97X-D provides quite reliable vertical excitation energies and oscillator strengths for π-conjugated systems^49,50^ and for charge-transfer states,^51,52^ making it suitable for exploring the photochemical properties of g-C_3_N_4_ fragments.

The ionization energy, defined as the energy required to remove the weakest-bound electron, was calculated vertically in all cases using the ground-state geometry of the neutral structure. The electron affinity, defined as the energy change of a neutral atom when an electron is added, has also been calculated by using ground-state geometry. All electronic structure calculations were performed using the Gaussian 16 software package.^53^

Charge transfer in the excited states of THZ was analyzed using the Theodore program,^54,55,57^ by performing a Löwdin-type population analysis of the squared transition density matrix elements using partial summations defining the matrix $\Omega_{AB}^{n\alpha }$ :

1 $$\Omega_{AB}^{n\alpha }=\frac{1}{2}\underset{a\epsilon A}{\sum }\underset{b\epsilon B}{\sum }{\left(D^{n\alpha ,\left[\mathrm{AO}\right]}S^{\left[\mathrm{AO}\right]}\right)}_{ab}{\left(S^{\left[\mathrm{AO}\right]}D^{n\alpha ,\left[\mathrm{AO}\right]}\right)}_{ab}$$

*D* ^*n*α,[AO]^ represents the one-particle transition density matrix, from state *n* to state α. *A* and *B* denote general definitions of molecular fragments; *S* ^[AO]^ is the overlap matrix in the atomic orbital (AO) basis, and the labels *a* and *b* refer to atomic orbitals.^56^ In the present case, the following fragments are defined (Figure 3): fragment I corresponds to the NH-substituted heptazine (always shown as the upper fragment), fragment II corresponds to the BH-substituted heptazine (always shown as the fragment on the right), and fragment III corresponds to the heptazine (always shown as the fragment on the left) (Figure S1 in the Supporting Information SI).

The selected bright states are those with oscillator strengths greater than 0.2. This approximate threshold was chosen to maintain consistency across the analyses. Natural transition orbitals (NTOs)^57^ are presented alongside the thermodynamic criteria for the first excited state.

The stability and reactivity of all systems studied were analyzed using two key metrics: the global stability index (ε^3^)^58^ and the electrophilicity reactivity index Δω ^±^.^59^ The ε^3^ stability function includes both kinetic and thermodynamic aspects of stability in terms of three properties. The properties that indicate reactivity and serve as kinetic descriptors include the ionization energy (IP) and the singlet–triplet (*E*_S–T_) energy, which represents a transition to the first excited state without spin restrictions. The thermodynamic descriptor corresponds to the Gibbs energy of atomization $\left(\Delta G_{\mathrm{atom},298K}^{{}^{\circ}}\right)$ . Based on these definitions, ε^3^ is given in eq 2 as

2 $$\varepsilon^{3}=\left|\mathrm{IP}\right|\cdot \left|E_{S-T}\right|\cdot \left|\Delta G_{\mathrm{atom},298K}^{{}^{\circ}}\right|$$

The reactivity of the studied molecules was evaluated using the net electrophilicity index Δω^±^, which quantifies the electrophilicity of a molecule relative to its own nucleophilicity.

3 $$\Delta \omega^{\pm }=\omega^{+}-\frac{1}{\omega^{-}}$$

4 $$\omega^{+}=\frac{{\left(\mathrm{IP}+3\mathrm{EA}\right)}^{2}}{16\left(\mathrm{IP}-\mathrm{EA}\right)}$$

5 $$\omega^{-}=\frac{{\left(3\mathrm{IP}+\mathrm{EA}\right)}^{2}}{16\left(\mathrm{IP}-\mathrm{EA}\right)}$$

In this metric, ω^+^ and ω^–^ represent the electron-accepting and electron-donating powers, respectively. IP and EA represent the ionization potential and electron affinity, respectively. Higher Δω^±^ values indicate a more reactive molecule with electrophilic character, while lower Δω^±^ values also indicate high reactivity but with nucleophilic character.^60,61^

Regarding the calibration of the methodology used in the present study, excited-state calculations were performed using the MS-CASPT2 ^62^ method to assess the reliability of the ωB97X-D results, as well as those obtained with other functionals that have shown accurate performance for reaction energies and barrier heights across large and chemically diverse datasets,^68^ namely CAM-B3LYP-D3 ^63–65^ and ωB97M-V.^50^ ADC(2)^69^ and SOS-ADC(2)^70^ were also considered; however, their D1 diagnostics were found to be too high to be considered reliable for these systems.^71^ The MS-CASPT2 calculations were carried out in MOLCAS^66^ with a complete active space of (8 e^–^, 9 o), and def2-SV­(P) basis set was used for all these calibration methods. The complete analysis is presented in the SI and includes comparisons for all substituted and pristine heptazine structures (Tables S1–S2 and Figure S2). For the THZ structures, the ωB97X-D results were compared with CAM-B3LYP-D3 (using Gaussian 16) and ωB97M-V (using ORCA 6.0 ^67^) (Tables S1–S3 and Figures S2–S3).

The effect of the implicit solvent (water) was analyzed and can be found in SI, Tables S5–S6 and Figures S8–S10. For the heptazine structures, geometry optimizations were performed using the Polarizable Continuum Model (PCM),^72^ followed by TD-DFT excitation calculations, including water as the solvent. For the THZ structures, single-point PCM calculations were carried out using the gas-phase structures.^73^

## Results and Discussion

### Calibration of the Methodology

A methodological calibration analysis was performed using MS-CASPT2 to describe the vertical excitation energies of both substituted and pristine heptazines, yielding reasonable agreement with the ωB97X-D results. Additional functionals, including ωB97M-V and CAM-B3LYP-D3, were also employed, confirming that the general trends discussed in this work remain consistent across different methods. Previous high-level calculations by Loos et al.^74^ provide a more accurate estimate of the lowest singlet excitation energy of pristine heptazine using a CC3/aug-cc-pVTZ + [CCSDT/6-31+G­(d) – CC3/6-31+G­(d)] protocol, reporting an S_1_ value of 2.69 eV. The vertical excitation energy obtained with MS-CASPT2 (2.56 eV) in the present study is consistent with this reference value, whereas the ωB97X-D functional predicts a slightly higher energy of 3.06 eV. Despite this 0.37 eV deviation, ωB97X-D captures the correct excitation ordering and offers a favorable balance between computational cost and accuracy, supporting its use in the subsequent analyses. The remaining functionals varied the S_1_ values relative to that of ωB97X-D by approximately 0.1 eV. A similar behavior is observed for the THZ structures, for which the three tested functionals (ωB97X-D, CAM-B3LYP-D3, and ωB97M-V) yield consistent trends in both excitation energies and oscillator strengths.

Concerning the solvent effects on heptazine structures, only minor variations were found in the oscillator strength and excitation energy values for most structures, with the exception of the BH-substituted heptazines, in which the first excited state presented a higher oscillator strength (structure 13), in some cases even surpassing the threshold used for a bright state (structures 14 and 15). For the THZ structures, the changes were mostly quantitative. Most bright states were slightly blue-shifted, while the S_1_ states were slightly red-shifted. It is worth noting that the oscillator strength of the S_1_ state also presented higher values for some structures (structures 18 and 19).

### Analysis of CN-Based Precursors for g-C_3_N_4_

In the first step, the thermodynamic criteria to evaluate the potential of the CN-based molecules and precursors for the formation of g-C_3_N_4_ (Figure 2b) were investigated. This approach aims to characterize these structures prior to studying the electronic structure changes introduced by BH and NH substitution. The thermodynamic criteria are presented in Figure 4.

**Figure 4:**
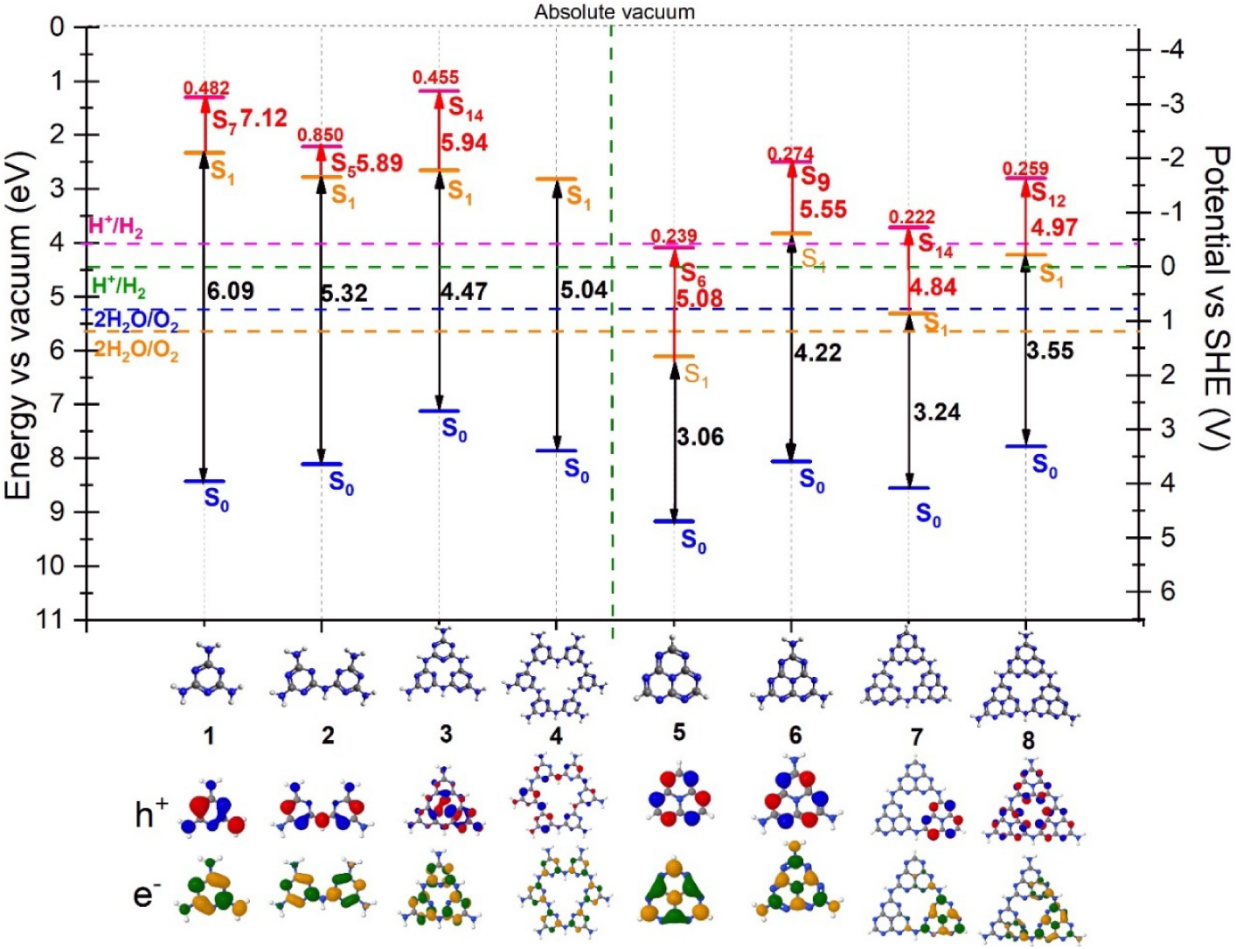
Thermodynamic criteria for g-C_3_N_4_ structures. The blue level represents the ionization energy level, the orange level corresponds to the first excited-state energy level, and the red level represents the excited state with the highest oscillator strength (bright state), with the actual oscillator strength on top of the red level. Below the graph, the natural transition orbitals (NTOs) for the S_1_ state are shown. To verify the complete list of energy and property values, as well as the associated orbitals, refer to Tables S7 and S8 in the SI.

The largest singlet energy gap corresponds to melamine (1) with 6.09 eV, followed by melam (2) with 5.32 eV. The single-ring-based structures (3 and 4) derived from s-triazine exhibit larger excitation energies of 4.47 eV for trimelamine (3) and 5.04 eV for poly­(triazine imide) (PTI) (4) compared to their heptazine-based counterparts, indicating greater thermodynamic stability with light absorption located above the visible light energy range. The structures (5, 6, 7, and 8) possess similar properties. Note that heptazine (5) has an excitation energy of 3.06 eV, while triheptazine (7) has an excitation energy of 3.24 eV, suggesting that they can function as building blocks for photocatalysts, allowing for the design of structures with larger ring sizes while maintaining similar electronic properties as the HZ monomer. Although these heptazine-based structures have an S_1_ excitation energy in the visible region, their efficiency remains low because their bright states are found at higher energies, outside the visible region.^75^

Poly­(triazine imide) (PTI) does not exhibit a bright state with an oscillator strength greater than 0.2 (as defined in this study) up to the S_20_ state. Therefore, it appears on the graph without an associated oscillator strength.

### Substitution Strategies and Their Effects on the Photocatalytic Properties of Heptazine

To study the substitutions and their effects on the photocatalytic properties of heptazine, three different substitution strategies are proposed in this study (Figure 5). The first is graphitic NH substitution of two N atoms (structures 9–12), where their positions have been varied, including all possible nonequivalent permutations. To maintain an even number of electrons, two NH substitution positions have been inserted always. It was observed previously that the graphitic substitution was more effective, leading to substantial red shifts in the case of substituted pyrene structures.^37^ The second substitution strategy is to remove two nitrogen atoms and replace them with two BH groups, permuting the BH positions through all possible permutations (structures 13–16). Again, two BH substitution events have always been added to maintain an even number of electrons. The final substitution involves graphitic substitution with two boron atoms, where two carbon atoms are removed and two boron atoms are added in their place (structure 17).

**Figure 5:**
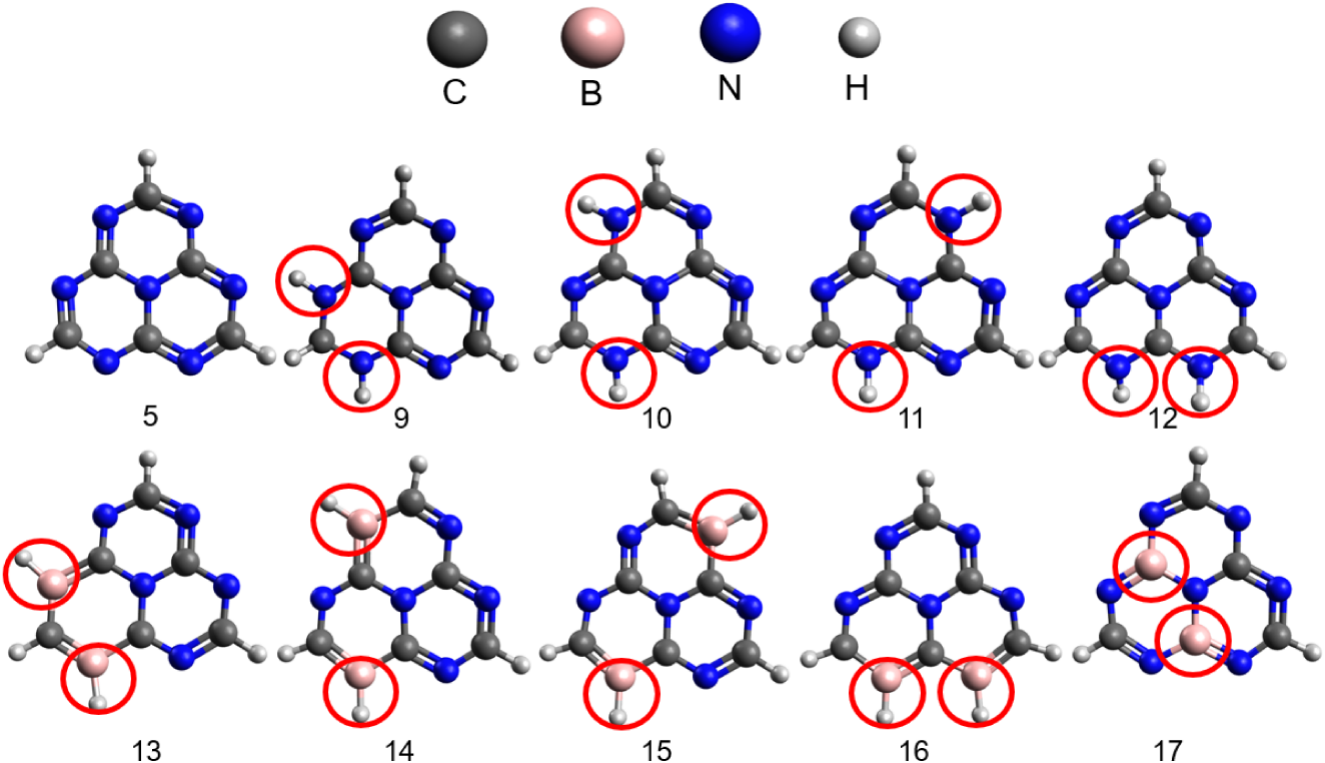
Proposed substitution strategies for heptazine (structure 5) are also shown as reference. Structures 9 to 12 represent NH substitution, in which two hydrogen atoms are added to nitrogen atoms at various positions. Structures 13 to 16 involve the substitution of a pyridinic nitrogen atom by a BH group, with all possible BH position permutations considered. Structure 17 illustrates a graphitic substitution, where two carbon atoms are replaced by two boron atoms. All substitution positions are highlighted by red circles.

The original heptazine (structure 5) adopts a planar configuration,^76,77^ where the lateral carbons attached to hydrogen atoms exhibit sp^2^ hybridization. However, when NH substitution is introduced within the same ring (structure 9), the hydrogen bonded to the carbon moves out of the plane and adopts a tetrahedral configuration (Table S9). Meanwhile, the dopant hydrogens bonded to the nitrogen atoms remain in a planar configuration. A similar deviation from planarity is observed when hydrogen dopants are added to the nitrogen atoms in different rings (structures 10 and 11), except for adjacent nitrogen dopants (structure 12). In the first two cases, the central nitrogen atom undergoes a structural shift and moves out of the plane. For structure 12, which involves substitution with two hydrogen atoms on the adjacent nitrogen atoms on different rings, these inserted hydrogens move out of the plane.

In contrast to the NH substitution, the structures substituted with two BHs (13–16) maintain planarity, as in the original heptazine. The B–H bond distances in structures 14, 15, and 16 are 1.20 Å, while in structure 13, where the substitution occurs within the same ring, a slight variation is observed, with a bond distance of 1.21 Å. All of these structures are shown in Table S9.

Analyzing the thermodynamic criteria of the substituted heptazine structures (Figure 6), structures 9, 10, and 11 exhibit smaller excitation energies and no bright states compared to unsubstituted heptazine (5), indicating lower stability. The group of NH- substituted structures has smaller ionization energies (located above the values for the pristine triazene (5) (Figure 6) and vanishing oscillator strength (structures 9, 10, and 11). In contrast, BH structures (structures 13–16) have a larger ionization energy, indicating greater stability compared with the NH-substituted counterparts. Consequently, they present a wider energy gap between the S_0_ and S_1_ states, along with considerable oscillator strength. These properties suggest their potential application as photosensitizers in photocatalytic mechanisms. Structure 17 belongs to a different class of substitution and represents a graphite-like substitution of the heptazine in which two boron atoms replace internal (graphitic) carbon atoms. Due to the symmetry of the heptazine, there is only one nonequivalent configuration for this disubstitution. Moreover, it results in unstable electronic properties and an absorption region outside the visible spectrum. Therefore, it indicates that for this class of molecules, boron substitutions should be performed on nitrogen atoms to enhance visible-light absorption rather than on carbon atoms.

**Figure 6:**
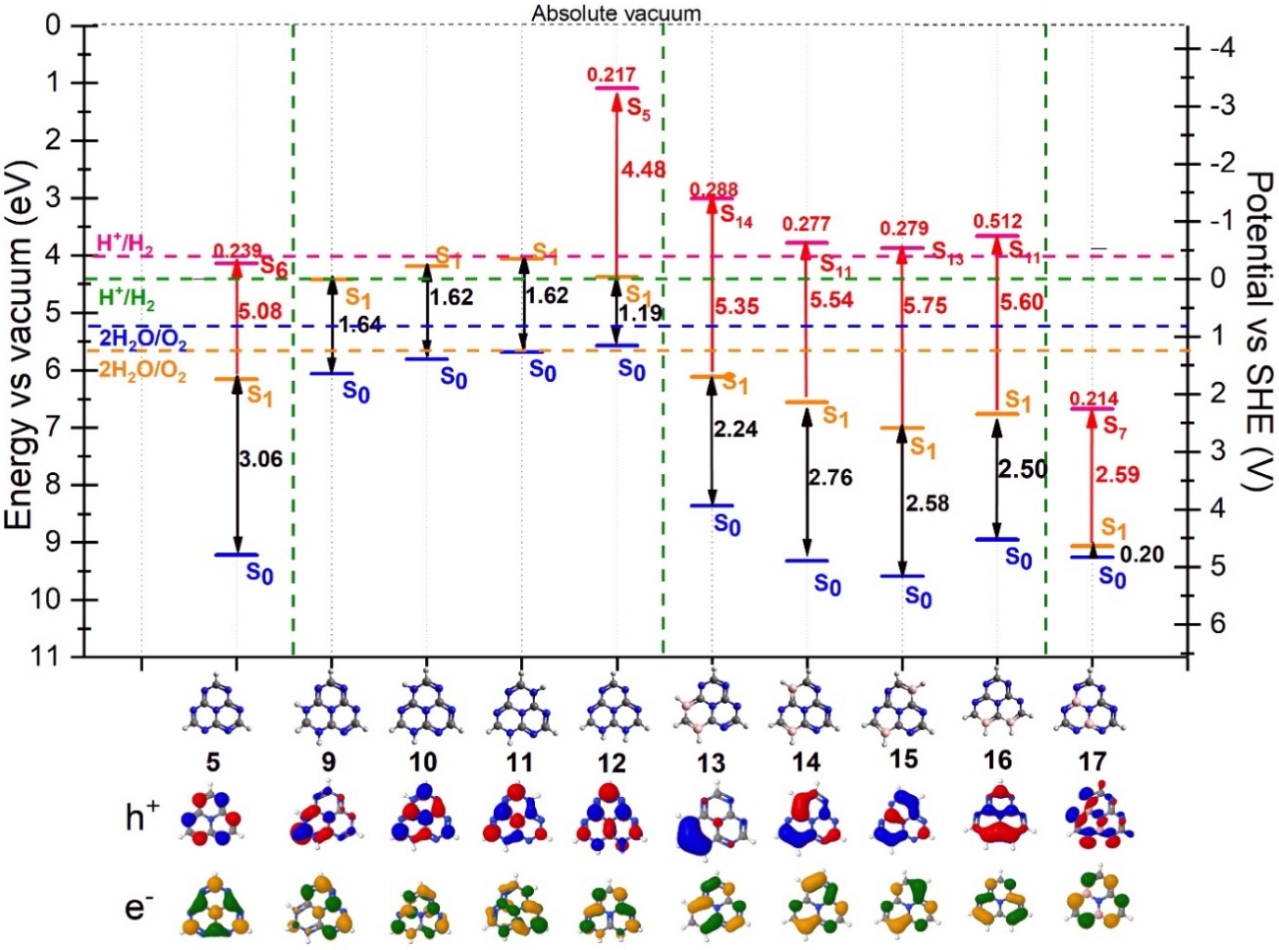
Thermodynamic criteria for the NH and BH substituted heptazine structures. The blue levels represent the ionization energy, the orange levels correspond to the first excited state, and the red levels represent the excited state with the highest oscillator strength (bright state), with the oscillator strength on top of the red level. Below the graph, the NTOs for the S_1_ state are also shown. To verify the complete list of energy and property values, refer to Table S10 through Table S20 in the SI.

Given these different characteristics, an interesting approach is to explore a structural model that integrates the properties of both types of substitutions to optimize the photocatalytic performance (see below).

### Stability ε^3^ and Reactivity Δω^±^ Analysis of Hydrogen- and BH-Substituted Heptazines

The stability and reactivity of NH- and BH-substituted heptazines and unsubstituted heptazines calculated using the ε^3^ index are presented in Figure 7. The NH-substituted structures 9–12 exhibit low stability, indicating significantly higher reactivity. In addition, their electrophilicity indices (Δω^±^), also presented in Figure 7, are below 0.6, classifying these structures as nucleophiles. In contrast, BH-substituted heptazines show significantly higher stability, with ε^3^ values comparable to the pristine heptazine, and their Δω^±^ values range from 0.6 to 1.3, classifying them as moderate electrophiles. Note that these analyses are in agreement with the ones discussed above, based on the S_0_ and S_1_ energy levels (Figure 6), which show comparable S_1_ energy between the BH-substituted and pristine heptazines, even though S_1_ is lower in energy in the BH-substituted structures (by around 0.5 eV).

**Figure 7:**
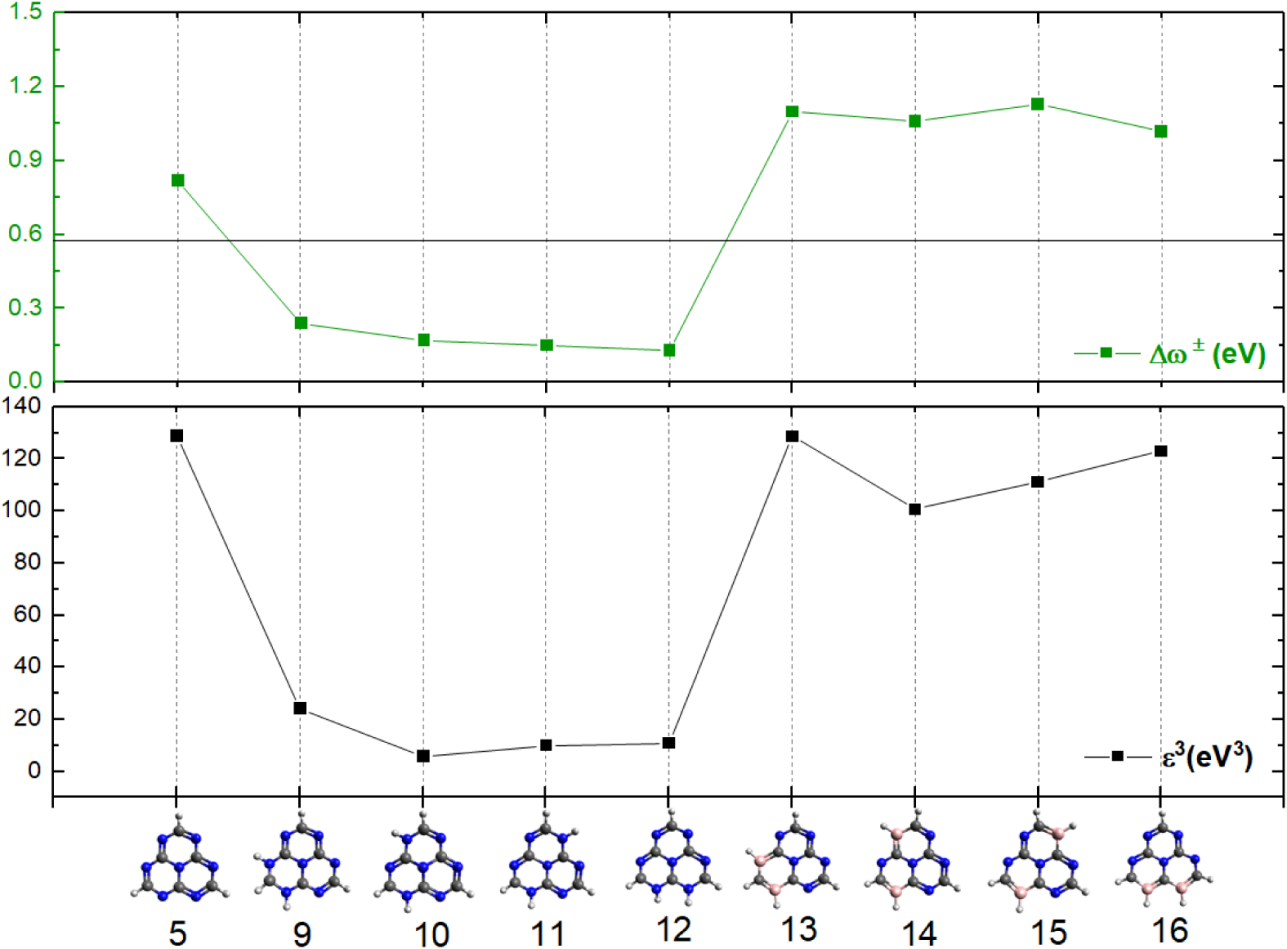
Schematic representation of the global stability index (ε^3^) and the net electrophilicity index (Δω^±^) used to evaluate the stability and reactivity of substituted and unsubstituted heptazines.

It is noted that, while substitution with NH results in a relatively unstable system (as indicated by the low Δω^±^ and ε^3^ values), other substituents can be used to stabilize these characteristics. We performed exploratory calculations on structure 9, changing its substituents (NH substituted) to high-electronegativity atoms, such as fluorine, chlorine, and the cyanide group. Some of these substitutions significantly improved stability, as evidenced by increases in Δω^±^ and ε^3^ values (see Figure S11). However, due to this increased stability, these substitutions also increase the excitation energies and shift the absorption spectrum toward the ultraviolet region. Therefore, a balance must be found between the stability and absorption of visible light for applications in photocatalysis.

### Structural Model for Optimized Photocatalysis: Combination of NH and BH Substitution in Tri-heptazine

The two primary substituent groups (NH and BH) do not individually fulfill the photocatalytic criteria formulated above. In particular, the creation of CT states requires the two features at the same time: an electron donor and an electron acceptor. Therefore, we propose a combination of BH substitution acting as the electron acceptor with the electron donor properties of the NH substitution. As described in section, these features were included into heptazine building blocks. The combination of these fragments within the THZ is shown in Figure 8. For comparison, the unsubstituted THZ (structure 7) is also shown.

**Figure 8:**
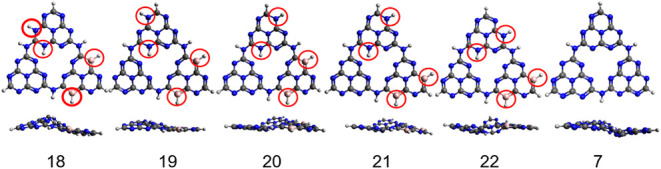
Combinations of NH and BH substituted THZ structures. Red circles highlight the positions of the NH and BH dopants.

From this point on, the main discussion focuses on excited-state charge transfer between different fragments, evaluation of the light absorption potential (with elimination of structures that do not show sufficient oscillator strength efficiency), and fulfillment of thermodynamic reduction criteria. The following structural principles were adopted: THZ configurations with BH in the central position were excluded due to strong deviations from planarity and possible proton transfers that would contaminate other previously defined fragments. Structures initiating proton transfer among different fragments were also discarded. Five structures fulfilled all these criteria and are presented in Figure 8.

Figure 9 shows the UV–Vis absorption spectra of the substituted THZ structures 18–22, in comparison to those of the pristine THZ structure 7. The complete list of excitation energies and oscillator strengths for structures 7 and 18–22 is given in Tables S21–S27.

**Figure 9:**
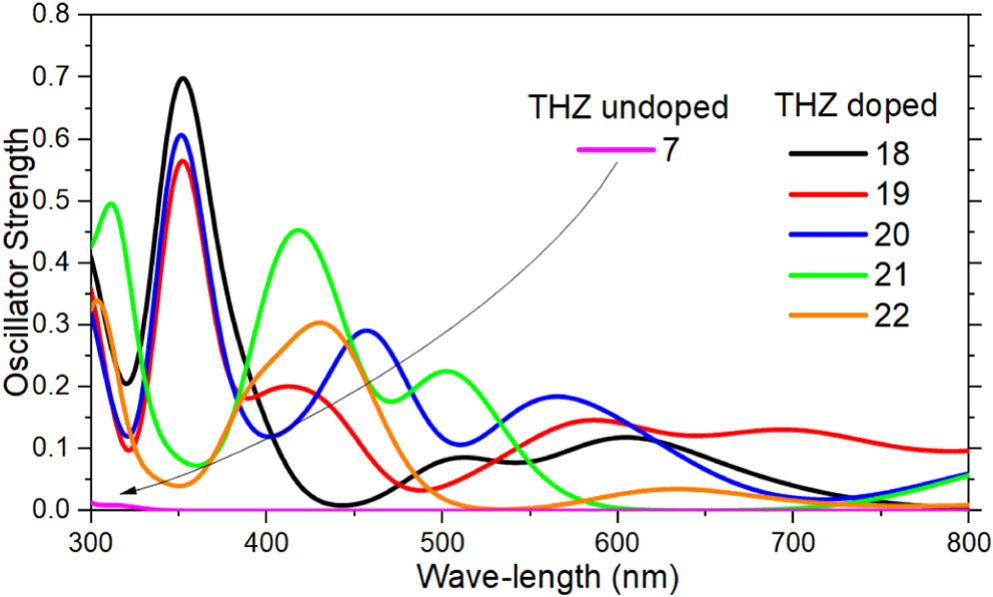
UV–Vis absorption spectra of the substituted THZ structures (18–22) in comparison to those of the pristine THZ (7).

The unsubstituted THZ (structure 7) presents by far the lowest absorption in the visible spectrum, while all substituted THZ structures possess enhanced absorption in this region. The 18–20 structures exhibit the strongest absorption in UV with peaks around 300 nm. On the other hand, structures 21 and 22 exhibit significant absorption near 420 nm, which is relevant for visible-light photocatalysis. The bright states are not located in the S_1_ state but rather at higher excited states. THZ 18 shows a bright state at S_10_; THZ 19 at S_12_; THZ 20 at S_6_; THZ 21 at S_4_; and THZ 22 at S_6_.

In addition to analyzing the absorption spectrum, the character of the CT in the S_1_ state (Figure 10) is of fundamental interest. Figure 10a shows the NTOs, and Figure 10b characterizes the extent of CT and local excitation (LE) character by means of a bar graph. The associated orbitals in each state and charge transfer between fragments are also given in Tables S21–S27 in the SI.

**Figure 10:**
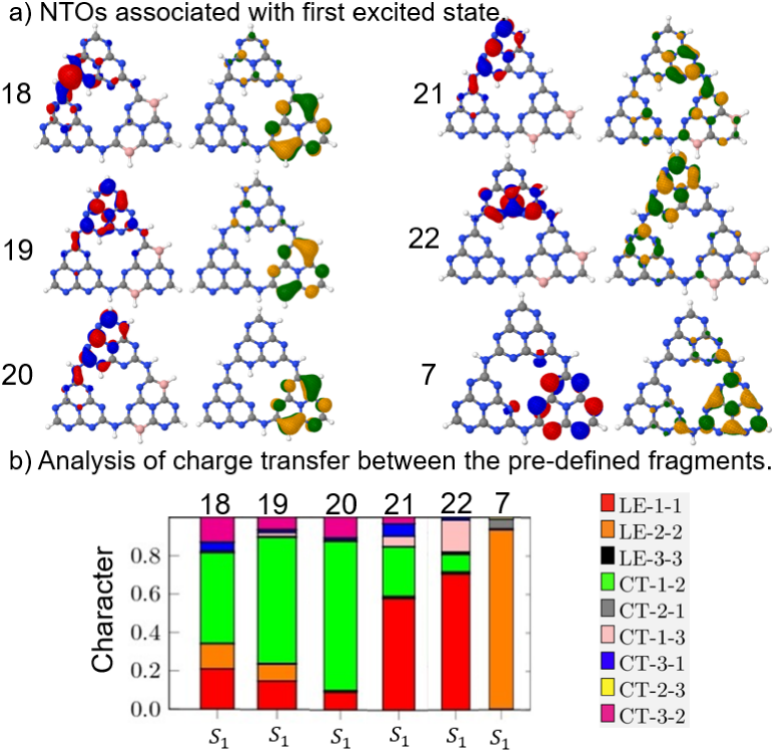
(a) NTOs for the S_1_ state of the substituted THZ structures. Hole distributions are represented by blue and red, and electron distributions by yellow and green colors, respectively. (b) Charge transfer analysis based on a bar graph for the first excited state. LE refers to local excitation within the same fragment, while CT presents interfragment excitations. Fragment 1 corresponds to NH substitution, fragment 2 corresponds to BH substitution, and fragment 3 corresponds to the unsubstituted THZ structure.

The substituted THZ structures 18, 19, and 20 exhibit pronounced charge transfer between fragments I and II, while THZ 21 and THZ 22 are dominated by LE processes and show only a reduced CT character. In the case of I–II transfer, the electron density moves from fragment I (substituted with NH) to fragment II (substituted with BH), indicating electron–hole pairs distributed across different fragments. For THZ 22, the charge transfer occurs from fragment I to fragment III.

Figure 11 shows the energy levels of NH (structure 9)- and BH (structure 15)-substituted heptazine structures and the entire THZ 18 structure, which is selected here because it fulfills the thermodynamic criteria for both the OER and HER processes. The ionization energy of THZ 18 is mainly determined by fragment I, which corresponds to the NH-substituted heptazine. All of the substituted THZ structures (THZ 18 to THZ 22) satisfy the thermodynamic requirements for the OER and/or HER half-reactions (Figure S12 in the SI). This means that the VB is located below the OER limit, and the CB­(S_1_) energy is above the HER limit. They also satisfy the other photocatalytic criteria discussed above, such as efficient visible light absorption and slow electron–hole recombination due to the large hole–electron separation in different HZ units. These findings suggest their potential as efficient and promising photocatalysts with great structural flexibility to induce photocatalytic reactions in dedicated regions of the differently substituted compounds.

**Figure 11:**
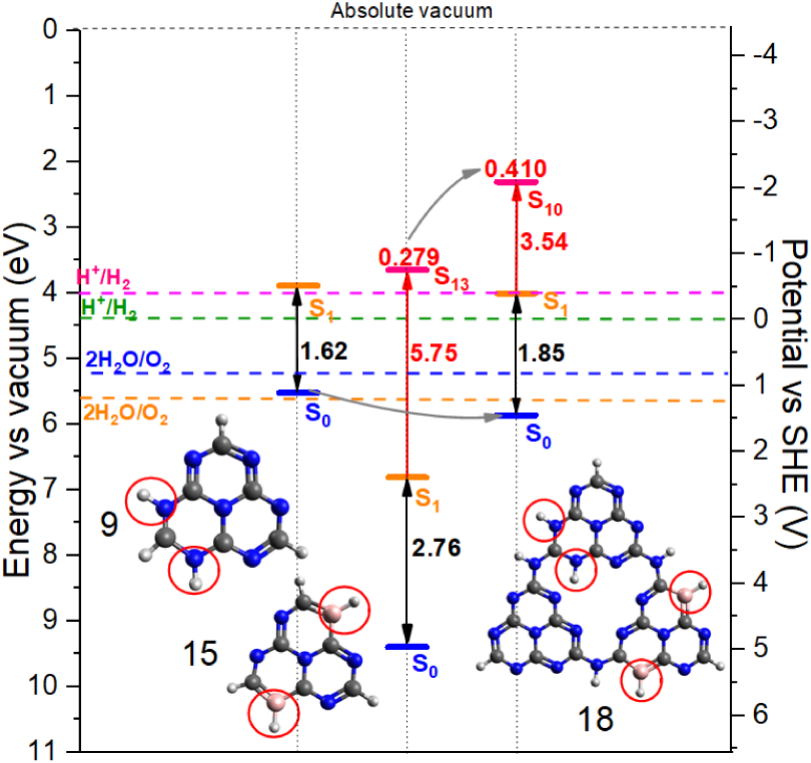
Thermodynamic criteria analysis for the THZ structure 18, in comparison to the energy levels of fragment I (NH substituted, structure 9) and fragment II (BH substituted, structure 15).

## Conclusion

Our investigations show the possibility of systematic construction of photocatalytic candidates based on g-C_3_N_4_ polymers by utilizing a B/N-substituted THZ model to create well-separated hole and electron CT states available for oxidation and reduction reactions. Besides the important properties of the CT states, descriptors for the chemical stability of the photocatalyst and the excitation energies have been given. The investigation of the thermodynamic and electronic properties of NH- and BH-substituted heptazine structures has provided detailed insights into their potential for photocatalytic applications. The NH-substituted heptazines exhibit enhanced reactivity due to their smaller energy gap, while the BH-substituted structures show significant oscillator strength, positioning them as effective photosensitizers. By combining these two substitution strategies, we propose a THZ model that synergistically integrates the advantages of both: lower ionization potential due to hydrogen substitution and robust light absorption due to BH substitution. These results corroborate the idea that NH substitution influences ionization energy, while BH substitution plays a critical role in enhancing light absorption, thereby contributing to the overall photocatalytic potential of the THZ model. Further investigation of the charge transfer properties and photocatalytic performance of these structures is essential to fully evaluate their suitability for water splitting, CO_2_ reduction, and other photocatalytic processes.

## Supplementary Material



## Data Availability

The data underlying this study are available in the published article and its Supporting Information.

## Notes

Comprehensive computational and spectroscopic data for the heptazine (HZ) and triheptazine (THZ) systems investigated in this work; vertical excitation energies, identification of the first excited (S_1_) and bright states, and corresponding oscillator strengths computed using multireference (MS-CASPT2) and several density functional theory approaches (ωB97X-D, ωB97M-V, and CAM-B3LYP-D3), both in the gas phase and in solvent; detailed comparisons across different heptazine derivatives and doping patterns, along with ionization energies and excited-state energetics; in-depth excited-state analyses based on natural transition orbitals (NTOs), including electron–hole pair character, orbital contributions, and charge-transfer features for selected structures; optimized geometries with bond lengths and angles, thermodynamic and reactivity descriptors, schematic representations of doped systems, and graphical summaries of excitation trends; finally, complete Cartesian coordinates of all optimized structures (PDF)
