# Supplementary material for: Tuning Heptazine-Based g‑C3N4 Structures for Photocatalysis by Enhancing Chemical Stability and Electron–Hole Pair Separation: A Computational Study
Source: ACS Omega. 2026 Jan 21;11(4):5170–80. doi: 10.1021/acsomega.5c07366 (PMC12878344; doi:10.1021/acsomega.5c07366)
Supplement: Supplementary file 1 [file ao5c07366_si_001.pdf]

Supporting Information:

Tuning Heptazine-Based g-C_3N_4 Structures for Photocatalysis by Enhancing Chemical Stability and Electron-Hole Pair Separation: A Computational Study

Leticia C. S. Faria[1,2]; Aditya N. Raju[3]; Julio, C. V. Chagas[1,4]; Adelia J. A. Aquino[5]; Reed Nieman[3]; Francisco B. C. Machado[1,2]; Leonardo T. Ueno[1,2]; Hans Lischka[3];* Luiz F. A. Ferrão[1,2].*

[1] Department of Chemistry, Aeronautics Institute of Technology, São José dos Campos 12228-900, Brazil.

[2] Advanced Scientific Computing and Modeling Laboratory, Aeronautics Institute of Technology, São José dos Campos 12228-900, Brazil.

[3] Department of Chemistry and Biochemistry, Texas Tech University, Lubbock TX 79409, United States of America.

[4] Department of Chemistry, Northwestern University, Evanston IL 60208, United States of America.

[5] Department of Mechanical Engineering, Texas Tech University, Lubbock TX 79409, United States of America.

Table Index

Table S1. Excitation energies (S_1 and S_N), bright states (S_N) and corresponding oscillator strengths for all heptazine (5, 9-16) structures calculated at the MS-CASPT(2) and ωB97X-D levels of theory using the def2-SV(P) basis set. 4
Table S2. Excitation energies (S_1 and S_N), bright states (S_N) and corresponding oscillator strengths for all heptazine (5, 9-16) structures calculated at the ωB97X-D, ωB97M-V, and CAM-B3LYP-D3 levels of theory using the def2-SV(P) basis set....................4
Table S3. Excitation energies (in eV) for the S_1 state and the corresponding bright state of each THZ structure, along with their respective oscillator strengths. The ωB97X-D, ωB97M-V and CAM-B3LYP-D3....................6
Table S4. Vertical excitation energies and oscillator strengths for the HZ system in solvent. S_1 energies, bright-state energies, and their corresponding oscillator strengths are reported to bright states S_N. 8
Table S5. Vertical excitation energies and oscillator strengths for the THZ system in solvent. S_1 energies, bright-state energies, and their corresponding oscillator strengths are reported to bright states S_N. 9
Table S6. Ionization energy (IE, eV), first excited state (S_1, eV), bright state (eV), oscillator strength of the bright state, and the corresponding bright state (1 to 8). 10

TABLE S7. EXCITED STATES (EV), ELECTRON/HOLE PAIR (NTOS), OSCILLATOR STRENGTHS, AND ORBITAL CONTRIBUTIONS (%) FOR EACH ANALYZED STATE. 11
TABLE S8. THE GEOMETRY OPTIMIZATION OF THE HEPTAZINE STRUCTURE IS PRESENTED, INCLUDING THE BOND LENGTHS (IN ANGSTROMS) AND ANGLES. 17
TABLE S9. IONIZATION ENERGY (IE, EV), FIRST EXCITED STATE (S_1, EV), BRIGHT STATE (EV), OSCILLATOR STRENGTH OF THE BRIGHT STATE, AND THE CORRESPONDING BRIGHT STATE (9 TO 19). 20
TABLE S10. EXCITED STATE ANALYSIS FOR HEPTAZINE STRUCTURE 5. EXCITED STATES (EV), ELECTRON/HOLE PAIR (NTOS), OSCILLATOR STRENGTHS, AND ORBITAL CONTRIBUTIONS (%) FOR EACH ANALYZED STATE. 20
TABLE S11. EXCITED STATE ANALYSIS FOR HEPTAZINE STRUCTURE 9. EXCITED STATES (EV), ELECTRON/HOLE PAIR (NTOS), OSCILLATOR STRENGTHS, AND ORBITAL CONTRIBUTIONS (%) FOR EACH ANALYZED STATE. 21
TABLE S12. EXCITED STATE ANALYSIS FOR HEPTAZINE STRUCTURE 10. EXCITED STATES (EV), ELECTRON/HOLE PAIR (NTOS), OSCILLATOR STRENGTHS, AND ORBITAL CONTRIBUTIONS (%) FOR EACH ANALYZED STATE. 22
TABLE S13. EXCITED STATE ANALYSIS FOR HEPTAZINE STRUCTURE 11. EXCITED STATES (EV), ELECTRON/HOLE PAIR (NTOS), OSCILLATOR STRENGTHS, AND ORBITAL CONTRIBUTIONS (%) FOR EACH ANALYZED STATE. 24
TABLE S14. EXCITED STATE ANALYSIS FOR HEPTAZINE STRUCTURE 12. EXCITED STATES (EV), ELECTRON/HOLE PAIR (NTOS), OSCILLATOR STRENGTHS, AND ORBITAL CONTRIBUTIONS (%) FOR EACH ANALYZED STATE. 25
TABLE S15. EXCITED STATE ANALYSIS FOR HEPTAZINE STRUCTURE 14. EXCITED STATES (EV), ELECTRON/HOLE PAIR (NTOS), OSCILLATOR STRENGTHS, AND ORBITAL CONTRIBUTIONS (%) FOR EACH ANALYZED STATE. 25
TABLE S16. EXCITED STATE ANALYSIS FOR HEPTAZINE STRUCTURE 14. EXCITED STATES (EV), ELECTRON/HOLE PAIR (NTOS), OSCILLATOR STRENGTHS, AND ORBITAL CONTRIBUTIONS (%) FOR EACH ANALYZED STATE. 28
TABLE S17. EXCITED STATE ANALYSIS FOR HEPTAZINE STRUCTURE 15. EXCITED STATES (EV), ELECTRON/HOLE PAIR (NTOS), OSCILLATOR STRENGTHS, AND ORBITAL CONTRIBUTIONS (%) FOR EACH ANALYZED STATE. 29
TABLE S18. EXCITED STATE ANALYSIS FOR HEPTAZINE STRUCTURE 16. EXCITED STATES (EV), ELECTRON/HOLE PAIR (NTOS), OSCILLATOR STRENGTHS, AND ORBITAL CONTRIBUTIONS (%) FOR EACH ANALYZED STATE.. 31
TABLE S19. EXCITED STATE ANALYSIS FOR HEPTAZINE STRUCTURE 17. EXCITED STATES (EV), ELECTRON/HOLE PAIR (NTOS), OSCILLATOR STRENGTHS, AND ORBITAL CONTRIBUTIONS (%) FOR EACH ANALYZED STATE. 33
TABLE S20. FIRST EXCITED STATE (S_1, EV), BRIGHT STATE (EV), OSCILLATOR STRENGTH OF THE BRIGHT STATE, AND THE CORRESPONDING BRIGHT STATE (18 TO 22 AND 7). 34
TABLE S21. EXCITED STATE ANALYSIS FOR HEPTAZINE STRUCTURE 7. EXCITED STATES (EV), ELECTRON/HOLE PAIR (NTOS), OSCILLATOR STRENGTHS, ORBITAL CONTRIBUTIONS (%) FOR EACH ANALYZED STATE AND CT (CHARGE TRANSFER). 35
TABLE S22. EXCITED STATE ANALYSIS FOR HEPTAZINE STRUCTURE 18. EXCITED STATES (EV), ELECTRON/HOLE PAIR (NTOS), OSCILLATOR STRENGTHS, ORBITAL CONTRIBUTIONS (%) FOR EACH ANALYZED STATE AND CT (CHARGE TRANSFER). 37
TABLE S23. EXCITED STATE ANALYSIS FOR HEPTAZINE STRUCTURE 19. EXCITED STATES (EV), ELECTRON/HOLE PAIR (NTOS), OSCILLATOR STRENGTHS, ORBITAL CONTRIBUTIONS (%) FOR EACH ANALYZED STATE AND CT (CHARGE TRANSFER). 39
TABLE S24. EXCITED STATE ANALYSIS FOR HEPTAZINE STRUCTURE 20. EXCITED STATES (EV), ELECTRON/HOLE PAIR (NTOS), OSCILLATOR STRENGTHS, ORBITAL CONTRIBUTIONS (%) FOR EACH ANALYZED STATE AND CT (CHARGE TRANSFER). 41

TABLE S25. EXCITED STATE ANALYSIS FOR HEPTAZINE STRUCTURE 21. EXCITED STATES (EV), ELECTRON/HOLE PAIR CHARACTERISTICS FROM NATURAL TRANSITION ORBITALS (NTOS), OSCILLATOR STRENGTHS, AND ORBITAL CONTRIBUTIONS (%) FOR EACH ANALYZED STATE. 43
TABLE S26. EXCITED STATE ANALYSIS FOR HEPTAZINE STRUCTURE 22. EXCITED STATES (EV), ELECTRON/HOLE PAIR CHARACTERISTICS FROM NATURAL TRANSITION ORBITALS (NTOS), OSCILLATOR STRENGTHS, AND ORBITAL CONTRIBUTIONS (%) FOR EACH ANALYZED STATE. 45

Figure Index

FIGURE S1. STRUCTURAL REPRESENTATION OF A DOPED TRI-HEPTAZINE (THZ) MOLECULE. FRAGMENT 1 (RED) CORRESPONDS TO THE HYDROGEN-DOPED UNIT, FRAGMENT 2 (GREEN) TO THE BORON-DOPED UNIT, AND FRAGMENT 3 (BLACK) TO THE UNDOPED UNIT. 4
FIGURE S2. EXCITATION ENERGIES (IN EV) FOR THE S_1 STATE AND THE CORRESPONDING BRIGHT STATE OF EACH HEPTAZINE STRUCTURE, ALONG WITH THEIR RESPECTIVE OSCILLATOR STRENGTHS. ΩB97X-D RESULTS ARE SHOWN IN BLACK, ΩB97M-V SHOW IN GREEN, CAM-B3LYP-D3 SHOW IN MAGENTA AND MS-CASPT2 IN RED. 5
FIGURE S3. EXCITATION ENERGIES (IN EV) FOR THE S_1 STATE AND THE CORRESPONDING BRIGHT STATE OF EACH THZ STRUCTURE, ALONG WITH THEIR RESPECTIVE OSCILLATOR STRENGTHS. THE ΩB97X-D RESULTS ARE SHOWN IN BLACK (S_1 STATE) AND GRAY (BRIGHT STATE), WHILE ΩB97M-V ARE REPRESENTED IN RED (S_1 STATE) AND LIGHT RED (BRIGHT STATE), AND CAM-B3LYP-D3 ARE REPRESENTED IN GREEN (S_1 STATE) AND LIGHT GREEN (BRIGHT STATE). 7
FIGURE S4. VERTICAL EXCITATION ENERGIES AND OSCILLATOR STRENGTHS FOR THE HEPTAZINE DERIVATIVES. S_1 ENERGIES IN THE GAS PHASE ARE SHOWN IN BLACK, AND THE CORRESPONDING BRIGHT STATES IN GRAY. S_1 ENERGIES IN SOLVENT (WATER) ARE SHOWN IN BLUE, AND THE BRIGHT STATES IN LIGHT BLUE. 8
FIGURE S5. VERTICAL EXCITATION ENERGIES AND OSCILLATOR STRENGTHS FOR THE THZ DERIVATIVES. S_1 ENERGIES IN THE GAS PHASE ARE SHOWN IN BLACK, AND THE CORRESPONDING BRIGHT STATES IN GRAY. S_1 ENERGIES IN SOLVENT (WATER) ARE SHOWN IN BLUE, AND THE BRIGHT STATES IN LIGHT BLUE. 10
FIGURE S6. SCHEMATIC REPRESENTATION OF THE GLOBAL STABILITY INDEX ($\varepsilon 3$) AND THE NET ELECTROPHILICITY INDEX ($\Delta\omega \pm$) USED TO EVALUATE THE STABILITY AND REACTIVITY OF DOPED AND UNDOPED HEPTAZINES. 34
FIGURE S7. THERMODYNAMIC CRITERIA FOR THZ DOPED STRUCTURES. THE BLUE LEVEL REPRESENTS THE IONIZATION ENERGY, THE ORANGE LEVEL CORRESPONDS TO THE FIRST EXCITED STATE, AND THE RED LEVEL REPRESENTS THE EXCITED STATE WITH THE HIGHEST OSCILLATOR STRENGTH (BRIGHT STATE), WITH THIS OSCILLATOR STRENGTH EXCEEDING THE RED LEVEL. THE LEFT VERTICAL AXES REPRESENT ENERGY VERSUS VACUUM IN EV AND THE STANDARD ELECTROCHEMICAL POTENTIAL (SHE). 47

Cartesian Coordinates 48

Figure S1. Structural representation of a doped tri-heptazine (THz) molecule. Fragment 1 (red) corresponds to the hydrogen-doped unit, Fragment 2 (green) to the boron-doped unit, and Fragment 3 (black) to the undoped unit.

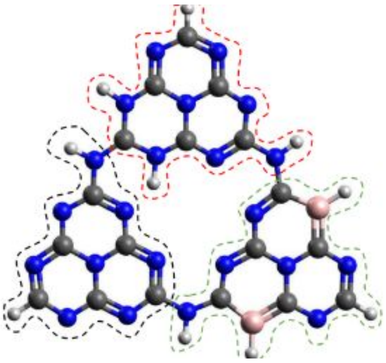

Table S1. Excitation energies (S_1 and S_n), bright states (S_n) and corresponding oscillator strengths for all heptazine (5, 9-16) structures calculated at the MS-CASPT(2) and ωB97X-D levels of theory using the def2-SV(P) basis set.

	S_1		Bright State		Oscillator Strengh		S_n	
HZ|	*MS	*ω	*MS	*ω	*MS	*ω	*MS	*ω
5	2.47	3.06	4.25	5.08	0.330	0.239	S_3	S_6
9	1.74	1.64	*	*	*	*	*	*
10	1.87	1.62	*	*	*	*	*	*
11	1.91	1.62	*	*	*	*	*	*
12	1.09	1.19	*	4.48	*	0.217	*	S_5
13	2.23	2.24	5.63	5.35	0.450	0.288	S_9	S_{14}
14	2.66	2.76	4.25	5.54	0.467	0.277	S_5	S_{11}
15	2.48	2.58	5.12	5.75	0.469	0.279	S_9	S_{13}
16	2.61	2.70	4.10	5.60	0.494	0.512	S_4	S_{11}

*MS stands for MS-CASPT2

*ω stands for ωB97X-D

Table S2. Excitation energies (S_1 and S_n), bright states (S_n) and corresponding oscillator strengths for all heptazine (5, 9-16) structures calculated at the ωB97X-D, ωB97M-V, and CAM-B3LYP-D3 levels of theory using the def2-SV(P) basis set.

	S_1			Bright State			Oscillator Strengh			S_n		
HZ	C*	v*	ω*	C*	v*	ω*	C*	v*	ω *	C*	v*	ω *
5	3.01	3.10	3.06	5.06	5.18	5.08	0.239	0.290	0.239	S_6	S_6	S_6

9	1.62	1.67	1.64	*	*	*	*	*	*	*	*	*
10	1.64	1.55	1.62	*	*	*	*	*	*	*	*	*
11	1.63	1.59	1.62	*	*	*	*	*	*	*	*	*
12	1.21	1.31	1.19	4.32	4.47	4.48	0.165	0.202	0.217	S_5	S_5	S_5
13	2.25	2.49	2.24	5.34	5.49	5.35	0.287	0.323	0.288	S_{14}	S_{13}	S_{14}
14	2.79	2.93	2.76	5.52	5.68	5.54	0.274	0.329	0.277	S_{11}	S_{11}	S_{11}
15	2.62	2.60	2.58	5.08	5.94	5.75	0.210	0.280	0.279	S_{10}	S_{14}	S_{13}
16	2.73	2.81	2.70	5.57	5.76	5.60	0.506	0.586	0.512	S_{11}	S_{11}	S_{11}

* C stands for CAM-B3LYP-D3

* v stands for ωB97M-V

*ω stands for ωB97X-D

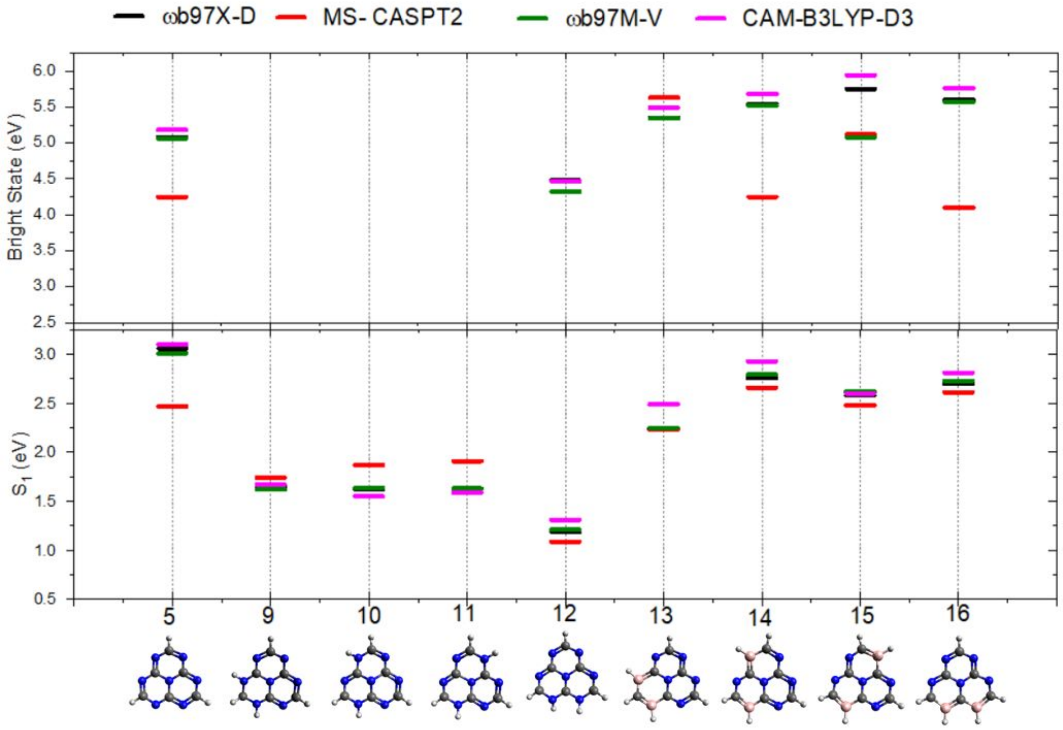

Figure S2. Excitation energies (in eV) for the S_1 state and the corresponding bright state of each heptazine structure, along with their respective oscillator strengths. ωB97X-D results are shown in black, ωB97M-V show in green, CAM-B3LYP-D3 show in magenta and MS-CASPT2 in red.

Although absolute excitation energies and oscillator strengths differ between ωB97X-D and MS-CASPT2 results, the overall trends across heptazine derivatives are consistent. For unsubstituted heptazine (Structure 5), the S_1 state is dark in both methods, with good agreement in excitation energies. The bright state appears at similar energies,

except for NH-12, which lacks a bright state in MS-CASPT2, supporting the view that photoluminescence is more likely associated with BH-substituted structures. Overall, ωB97X-D provides an adequate description of the excitation spectrum, with similar trends observed using ωB97M-V and CAM-B3LYP-D3.

Complementing the calibration analysis, additional investigations were carried out for the THZs. Due to the computational cost of MS-CASPT2 for these systems, excitation energies (S1 and the bright state) were evaluated using the same two additional functionals used for heptazine structures, CAM-B3LYP-D3 and ωB97M-V. The results are presented alongside those obtained with the primary functional employed, ωB97X-D (see Figure S3 and Table S3).

Table S3. Excitation energies (in eV) for the S_1 state and the corresponding bright state of each THZ structure, along with their respective oscillator strengths. The ωB97X-D, ωB97M-V and CAM-B3LYP-D3.

	S_1			Bright State			Oscillator Strengh			S_n		
THZ	C*	v*	ω*	C*	v*	ω*	C*	v*	ω *	C*	v*	ω *
18	1.47	2.04	1.85	3.54	3.62	3.54	0.400	0.320	0.410	S_{11}	S_9	S_{10}
19	0.77	1.09	0.55	3.49	3.64	3.51	0.524	0.379	0.579	S_{12}	S_{11}	S_{12}
20	0.44	1.07	0.72	2.47	2.56	2.62	0.227	0.208	0.293	S6	S_5	S_6
21	0.93	1.48	1.21	2.24	2.71	2.39	0.150	0.211	0.251	S4	S_4	S_4
22	1.23	1.57	1.37	2.83	2.89	3.09	0.279	0.266	0.165	S6	S_4	S_6
7	3.19	3.24	3.24	4.81	5.09	4.84	0.300	0.901	0.222	S_{16}	S_{14}	S_{14}

* C stands for CAM-B3LYP-D3

* v stands for ωB97M-V

*ω stands for ωB97X-D

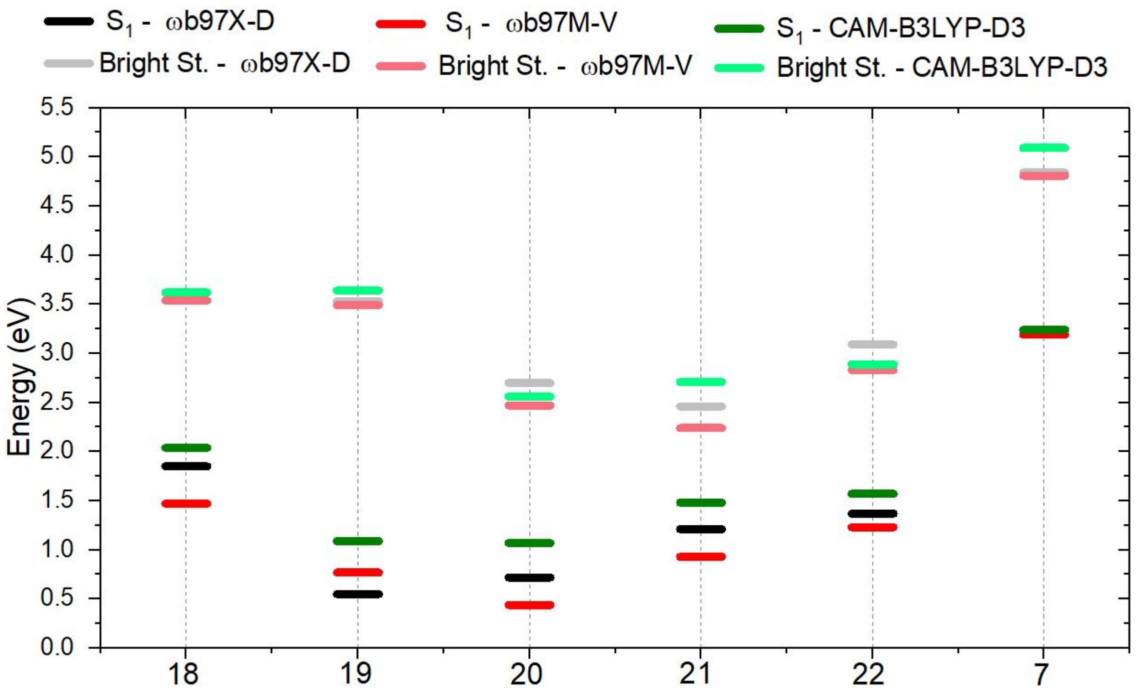

Figure S3. Excitation energies (in eV) for the S_1 state and the corresponding bright state of each THZ structure, along with their respective oscillator strengths. The ωB97X-D results are shown in black (S_1 state) and gray (bright state), while ωB97M-V are represented in red (S_1 state) and light red (bright state), and CAM-B3LYP-D3 are represented in green (S_1 state) and light green (bright state).

Figure S3 shows good agreement in excitation energies across the DFT functionals. The ωB97X-D functional reliably characterizes heptazine excited states, capturing both the charge-transfer (CT) and the brightest singlet states relevant for photocatalysis.

Additionally, we analyzed the stability of the closed-shell ωB97X-D solutions for all heptazine and THZ structures studied. All heptazine structures, as well as THZ18 and THZ22, exhibited stable solutions. Structures THZ20 and THZ21 showed minor relaxation of 0.01 eV after initially displaying slight closed-shell instability, whereas THZ19 exhibited a somewhat larger relaxation of 0.15 eV. These closed-shell instabilities were corrected via relaxation and the relaxed solutions were used for all subsequent analyses, such as excitation energies and oscillator strengths.

Analysis of Solvent Effects (Water PCM) on HZ and THZ Systems.

Table S4. Vertical excitation energies and oscillator strengths for the HZ system in solvent. S_1 energies, bright-state energies, and their corresponding oscillator strengths are reported to bright states S_n.

	S_1		Bright State		Oscillator Strengh		S_n	
HZ	Gas	Water solvent	Gas	Water solvent	Gas	Water solvent	Gas	Water solvent
5	3.06	3.20	5.08	5.09	0.239	0.312	S_6	S_5
9	1.64	1.70	*	*	*	*	*	*
10	1.62	1.56	*	*	*	*	*	*
11	1.62	1.35	*	*	*	*	*	*
12	1.19	1.21	4.48	4.66	0.217	0.249	S_5	S_4
13	2.24	2.62	5.35	5.35	0.288	0.642	S_{14}	S_{13}
14	2.76	2.89	5.54	2.89	0.277	0.276	S_{11}	S_1
15	2.58	2.49	5.75	2.49	0.279	0.260	S_{13}	S_1
16	2.70	2.81	5.60	3.34	0.512	0.221	S_{11}	S_2

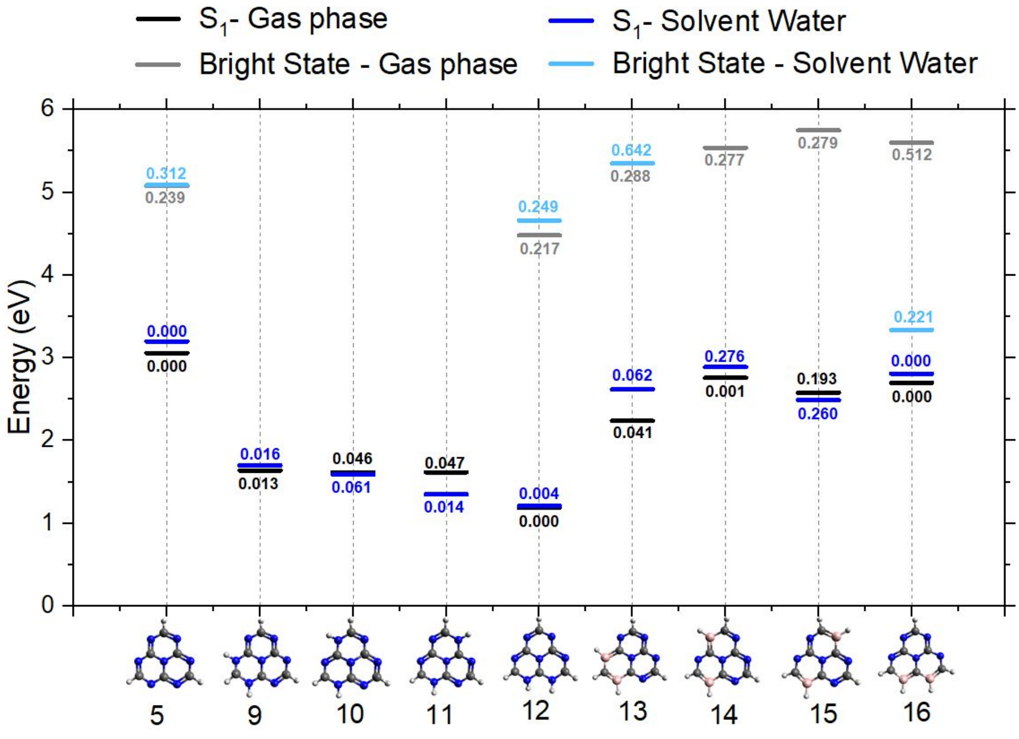

Figure S4. Vertical excitation energies and oscillator strengths for the heptazine derivatives. S_1 energies in the gas phase are shown in black, and the corresponding bright

states in gray. S_1 energies in solvent (water) are shown in blue, and the bright states in light blue.

Energy and oscillator strength values remain within the same region, with only small deviations (~0.2 eV). NH-substituted heptazines show no bright states in the region of interest, even when including the implicit solvent, whereas BH-substituted heptazines exhibit increased S_1 absorption intensities, confirming them as bright states. This trend indicates that the implicit solvent effect does not significantly alter the overall photophysical behavior.

Similarly, the investigation of the THZ system in the solvent environment is presented below, along with the S_1 values, the bright state, the oscillator strength associated with the bright state, and the S_n energies. The NTO orbitals are also shown.

Table S5. Vertical excitation energies and oscillator strengths for the THZ system in solvent. S_1 energies, bright-state energies, and their corresponding oscillator strengths are reported to bright states S_n.

	S_1		Bright State		Oscillator Strengh		S_n	
THZ	Gas	Water-PCM	Gas	Water-PCM	Gas	Water-PCM	Gas	Water-PCM
18	1.85	1.95	3.54	3.37	0.410	0.585	S_{10}	S_8
19	0.55	0.98	3.51	3.53	0.579	0.539	S_{12}	S_{12}
20	0.72	0.81	2.62	2.10	0.293	0.470	S_6	S_5
21	1.21	1.54	2.39	2.82	0.251	0.563	S_4	S_5
22	1.37	1.52	3.09	2.76	0.165	0.366	S_6	S_4
7	3.24	3.36	4.84	4.91	0.222	0.798	S_{14}	S_{14}

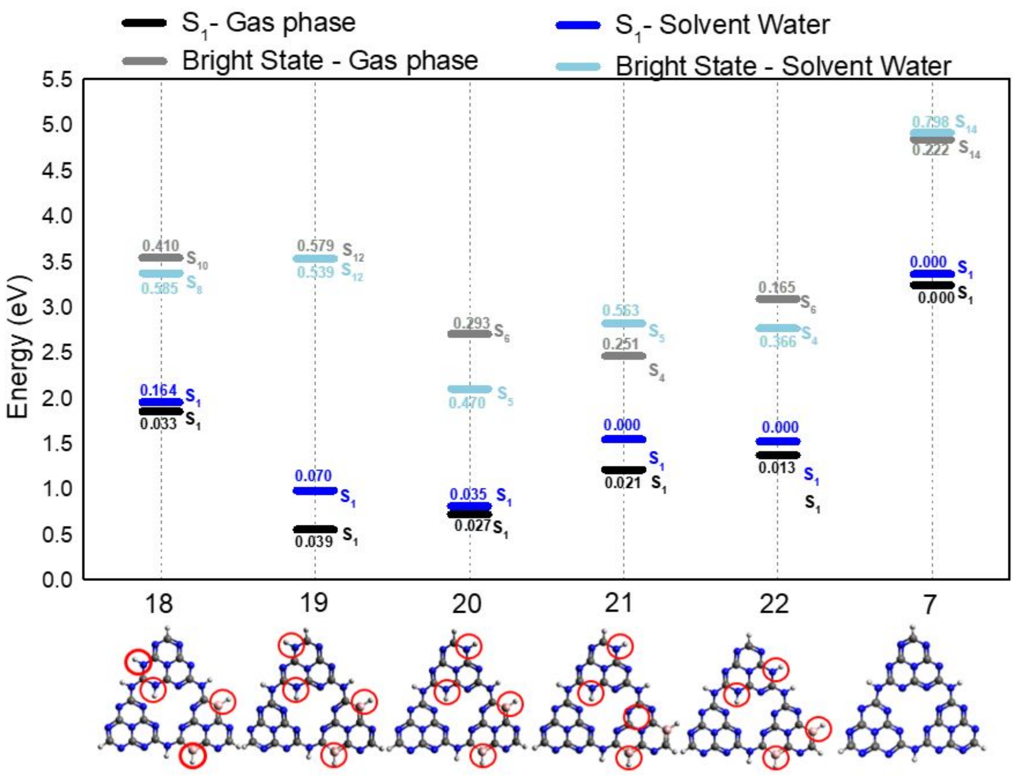

Figure S5. Vertical excitation energies and oscillator strengths for the THZ derivatives. S_1 energies in the gas phase are shown in black, and the corresponding bright states in gray. S_1 energies in solvent (water) are shown in blue, and the bright states in light blue.

For the THZ derivatives, we observe a trend similar to that of HZ: the water PCM environment increases the oscillator strength of the bright state while keeping it in the same spectral region as in the gas phase.

Table S6. Ionization energy (IE, eV), first excited state (S_1, eV), bright state (eV), oscillator strength of the bright state, and the corresponding bright state (1 to 8).

	IE (eV)	S_1 (eV)	Bright state (eV)	Oscillator strengh	State
1- Melamine	8.42	6.09	7.12	0.482	7th
2- Melam	8.10	5.32	5.89	0.850	5th
3- Tri-melamine	7.12	4.47	5.67	0.126	8th
4- PTI	7.86	5.04	5.59	0.111	12th
5- Heptazine	9.17	3.06	5.08	0.239	6th
6- Tri-heptazine	8.22	3.09	4.21	0.188	15th
7- Melem	8.05	4.22	5.55	0.274	5th

8- Melon	7.77	3.55	4.21	0.104	12th

Table S7. Excited states (eV), electron/hole pair (NTOs), oscillator strengths, and orbital contributions (%) for each analyzed state.

Excited State-Melamine	Electron	Hole	Orbital	E(eV)	Oscillator strength
1st	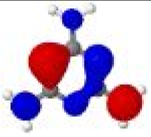	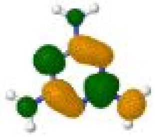	50% $(\pi\rightarrow\pi^*)$	6.09	0.000
2nd	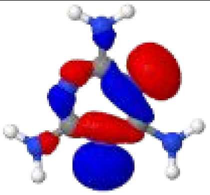	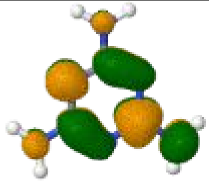	51% $(n\rightarrow\pi^*)$	6.22	0.000
3rd	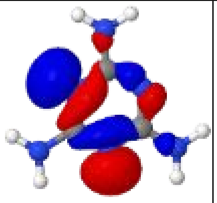	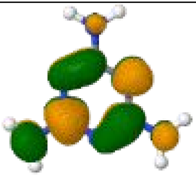	49% $(n\rightarrow\pi^*)$	6.44	0.000
4th	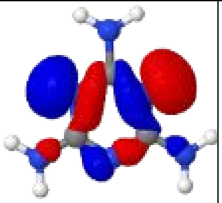	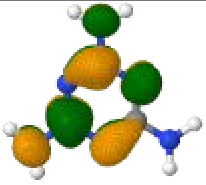	51% $(n\rightarrow\pi^*)$	6.44	0.000
5th	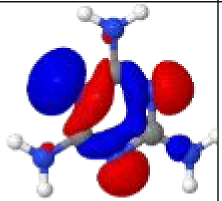	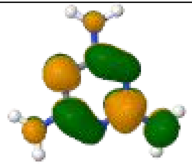	50% $(n\rightarrow\pi^*)$	6.61	0.008
6th	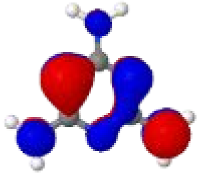	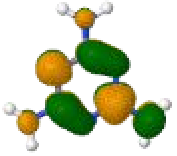	49% $(\pi\rightarrow\pi^*)$	7.00	0.000
7th	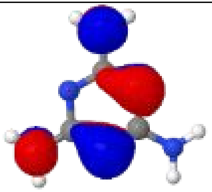	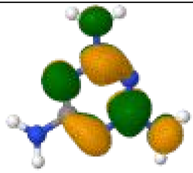	48% $(\pi\rightarrow\pi^*)$	7.12	0.482

8th	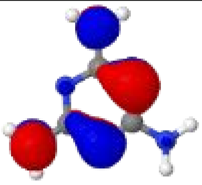	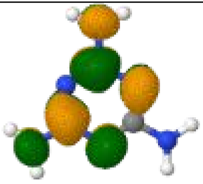	48% (π→π*)	7.12	0.482
9th	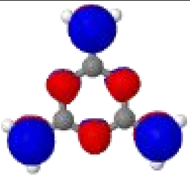	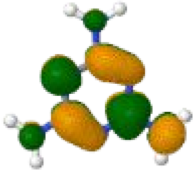	98% (π→π*)	7.69	0.175
Excited State-Melam	Electron	Hole	Orbital	E(eV)	Oscillator strength
1st	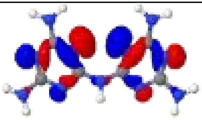	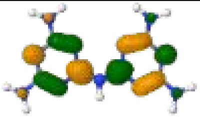	50% (n→π*)	5.32	0.002
2nd	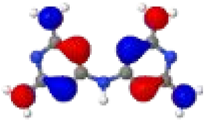	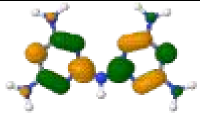	51% (π →π*)	5.77	0.039
3rd	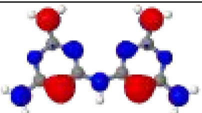	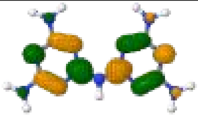	49% (π→π*)	5.83	0.111
4th	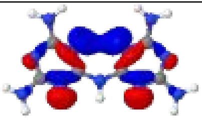	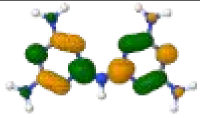	51% (n→π*)	5.83	0.004
5th	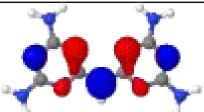	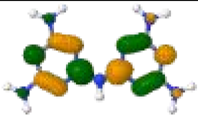	50% (π→π*)	5.89	0.851
6th	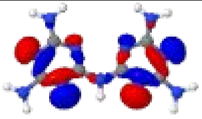	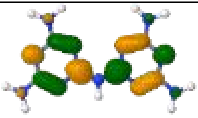	49% (n→π*)	5.99	0.005
Excited State-Tri-melamine	Electron	Hole	Orbital	E(eV)	Oscillator strength
1st	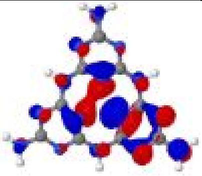	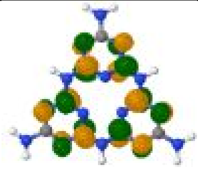	82% (n→π*)	4.47	0.001
2nd	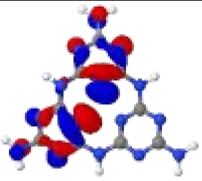	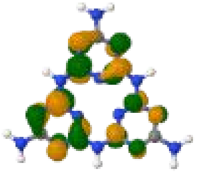	85% (n →π*)	4.49	0.011

3rd	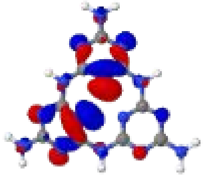	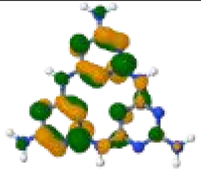	59% (*n*→*π**)	5.03	0.012
4th	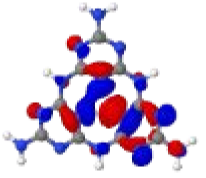	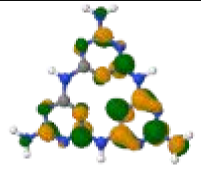	65% (*n*→*π**)	5.09	0.002
5th	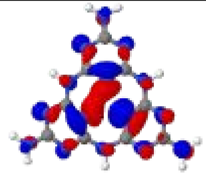	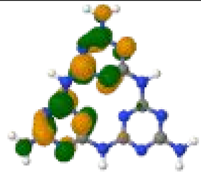	56% (*n*→*π**)	5.32	0.000
6th	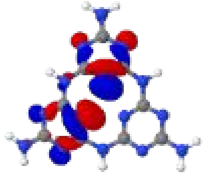	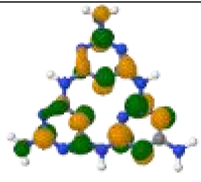	61% (*n*→*π**)	5.40	0.002
7th	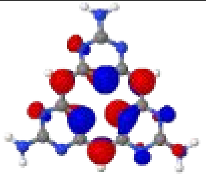	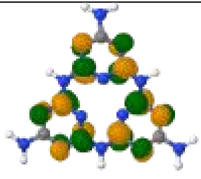	40% (*n*→*π**)	5.51	0.007
8th	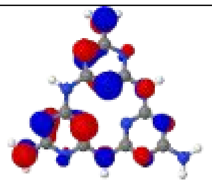	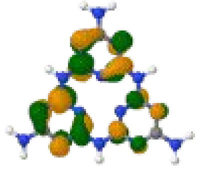	62% (*n* →*π**)	5.67	0.126
9th	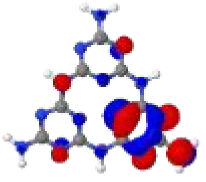	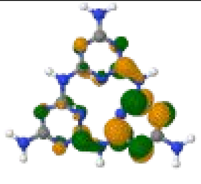	74% (*π*→*π**)	5.68	0.069
10th	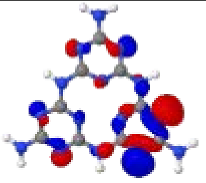	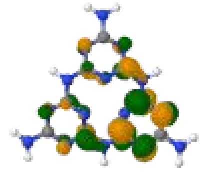	71% (*n*→*π**)	5.79	0.052
11th	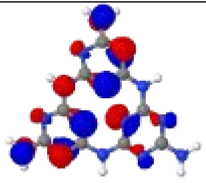	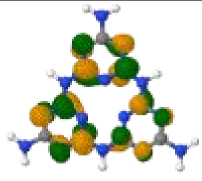	42% (*π*→*π**)	5.79	0.057
12th	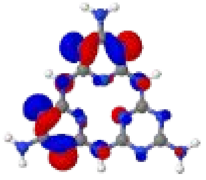	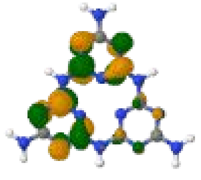	58% (*n*→*π**)	5.90	0.044

13th	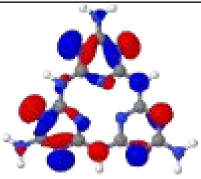	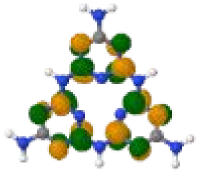	58% ($n\rightarrow\pi^*$)	5.90	0.160
14th	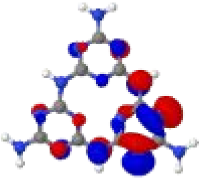	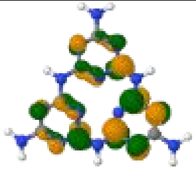	69% ($n\rightarrow\pi^*$)	5.94	0.455
15th	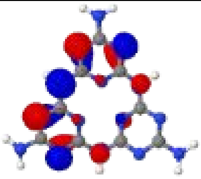	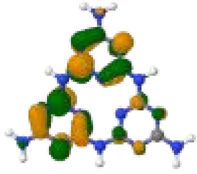	67% ($n\rightarrow\pi^*$)	6.05	0.543
16th	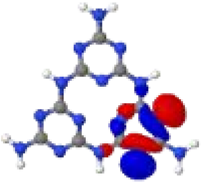	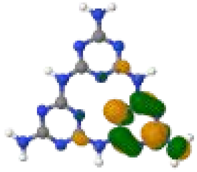	82% ($n\rightarrow\pi^*$)	6.14	0.029
Excited State-PTI	Electron	Hole	Orbital	E(eV)	Oscillator strength
1st	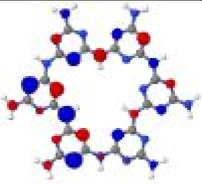	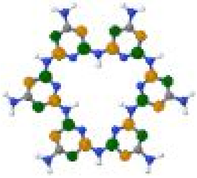	45% ($\pi\rightarrow\pi^*$)	5.04	0.000
Excited State-Melem	Electron	Hole	Orbital	E(eV)	Oscillator strength
1st	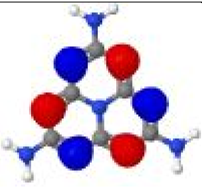	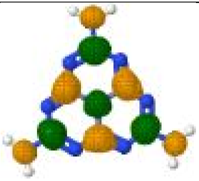	97% ($\pi\rightarrow\pi^*$)	4.08	0.000
2nd	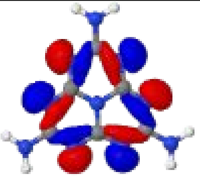	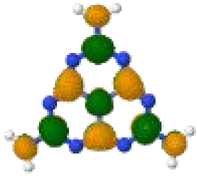	88% ($n\rightarrow\pi^*$)	4.76	0.000
3rd	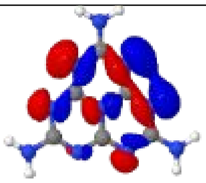	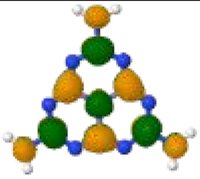	88% ($n\rightarrow\pi^*$)	4.81	0.000
4th	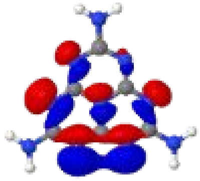	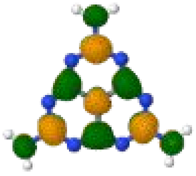	87% ($n\rightarrow\pi^*$)	4.81	0.000

5th	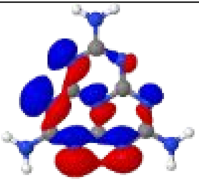	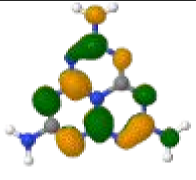	49% (n→π*)	5.34	0.007
Excited State-Melom	Electron	Hole	Orbital	E(eV)	Oscillator strength
1st	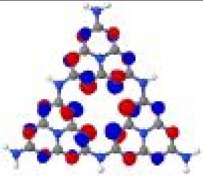	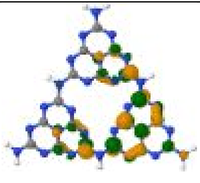	73% (n→π*)	3.55	0.001
2nd	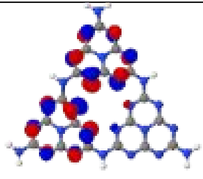	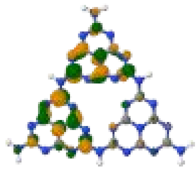	65% (π →π*)	3.59	0.013
3rd	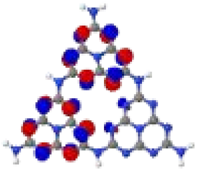	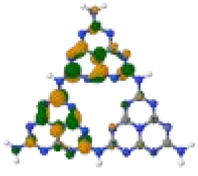	39% (π→π*)	3.72	0.009
4th	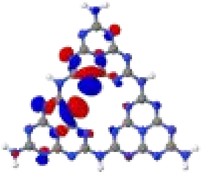	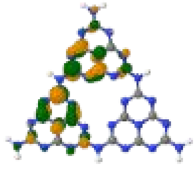	65% (n→π*)	4.08	0.008
5th	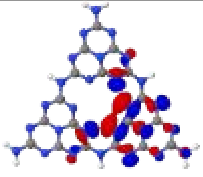	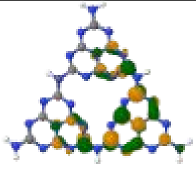	44% (n→π*)	4.31	0.001
6th	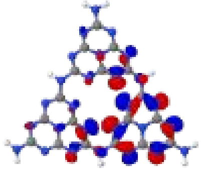	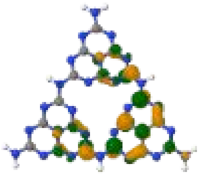	46% (n→π*)	4.34	0.000
7th	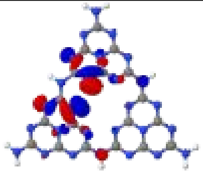	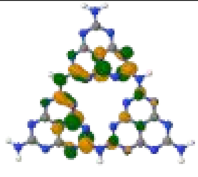	49% (n→π*)	4.50	0.004
8th	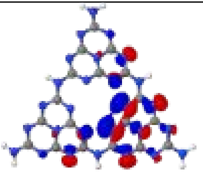	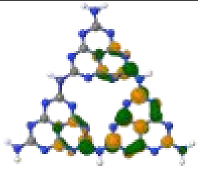	43% (n →π*)	4.59	0.002

9th	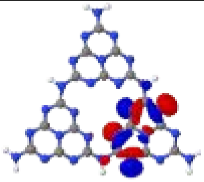	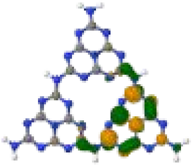	59% $(n\rightarrow\pi^*)$	4.59	0.000
10th	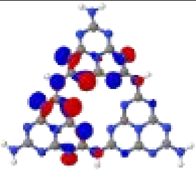	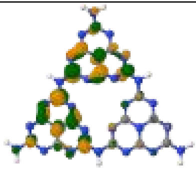	41% $(n\rightarrow\pi^*)$	4.62	0.008
11th	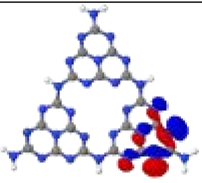	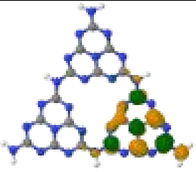	84% $(n\rightarrow\pi^*)$	4.66	0.005
12th	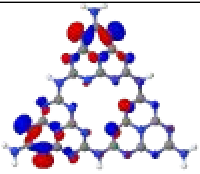	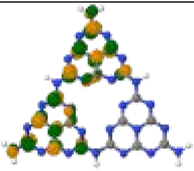	50% $(n\rightarrow\pi^*)$	4.93	0.004
13th	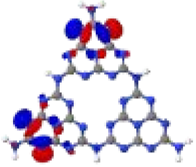	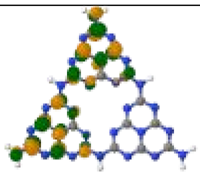	50% $(n\rightarrow\pi^*)$	4.95	0.003
14th	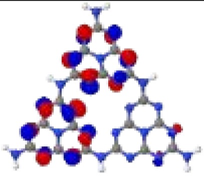	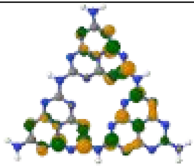	46% $(\pi\rightarrow\pi^*)$	4.97	0.259
15th	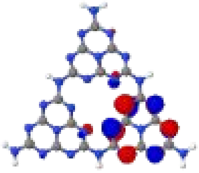	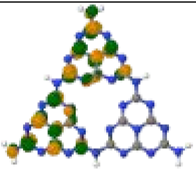	70% $(n\rightarrow\pi^*)$	4.99	0.182

Table S8. The geometry optimization of the heptazine structure is presented, including the bond lengths (in angstroms) and angles.

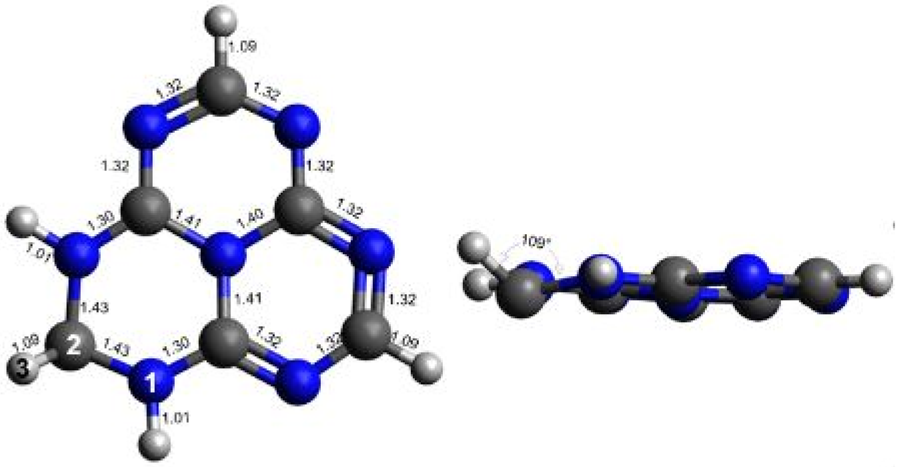
9- The angle between N1, C2 and H3 is 109°
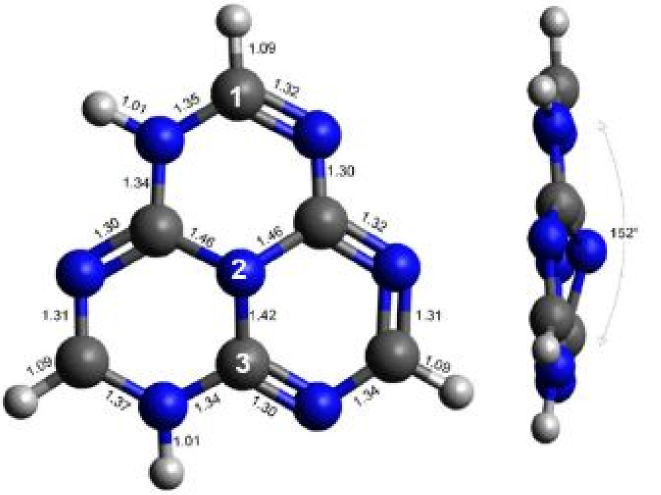
10- The angle between C1, N2 and C3 is 152°
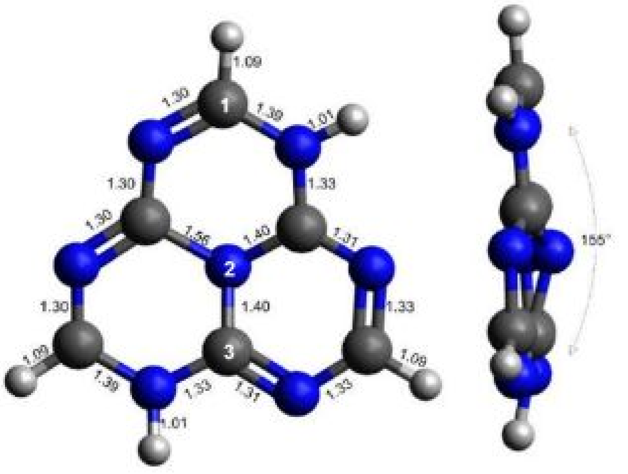
11- The angle between C1, N2 and C3 is 155°

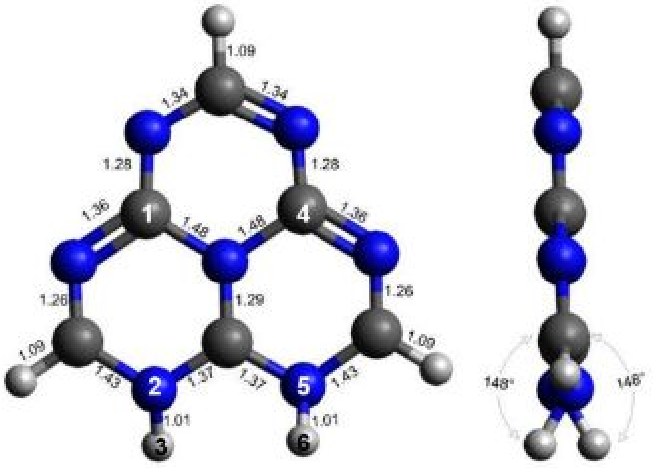

12- The angle between C1, N2, and H3 is 148°, and C4, N5, and H6 is also 148°.

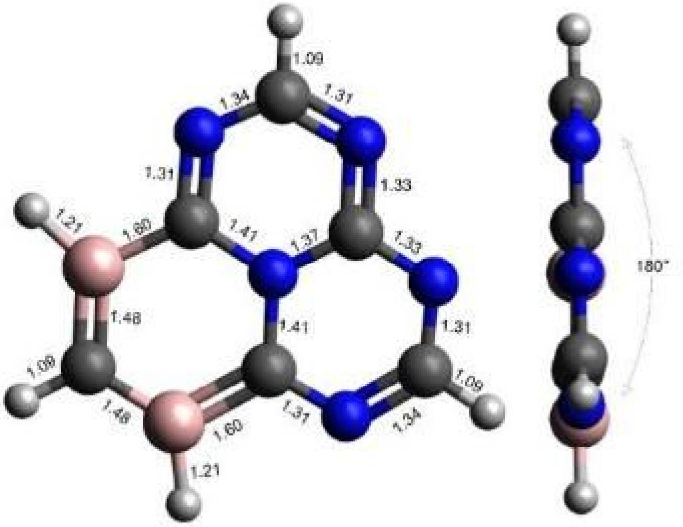

13

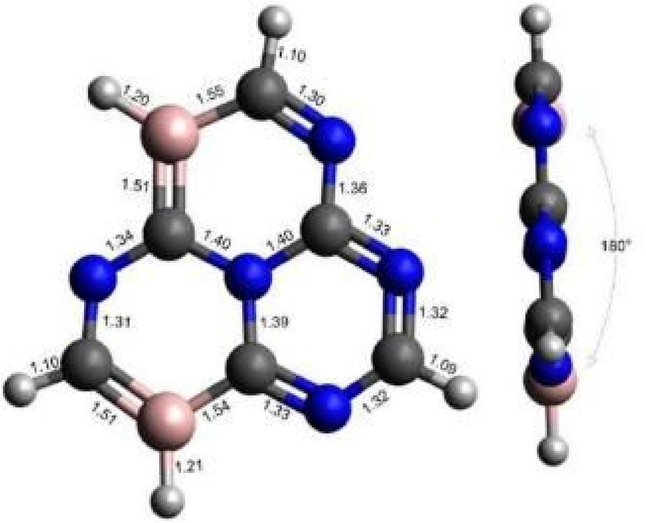

14

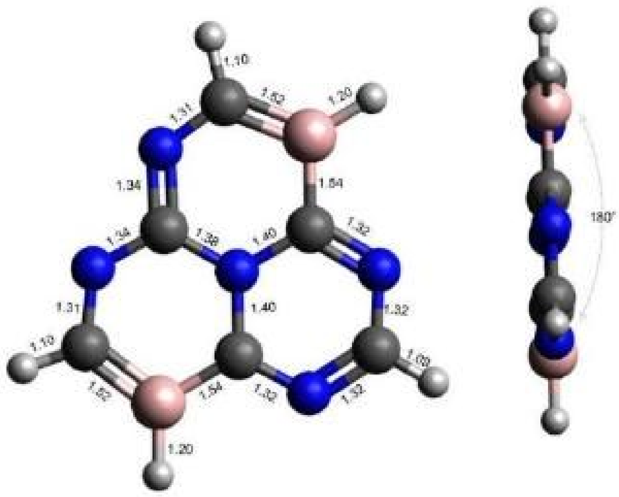
15
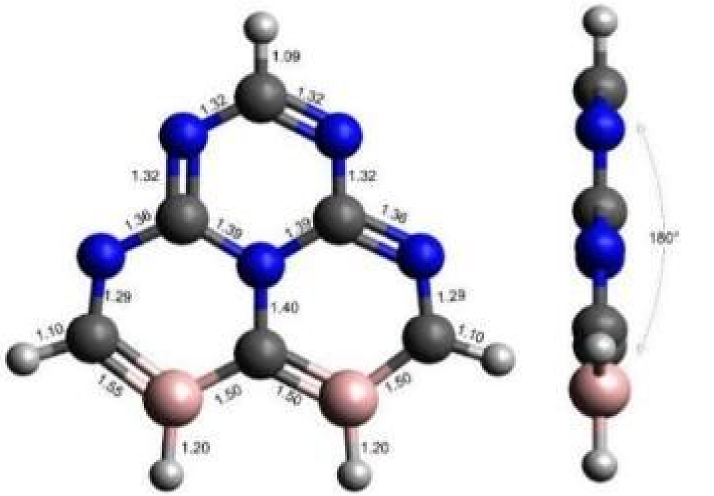
16
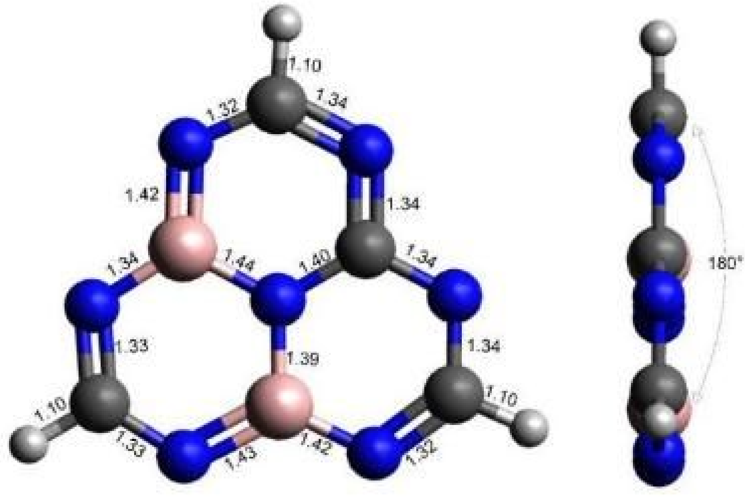
17

Table S9. Ionization energy (IE, eV), first excited state (S_1, eV), bright state (eV), oscillator strength of the bright state, and the corresponding bright state (9 to 19).

	IE (eV)	S_1 (eV)	Bright state (eV)	Oscillator strengh	State
Hep 5	9.22	3.06	5.08	0.239	6th
Hep 9	6.06	1.64	*	*	*
Hep 10	5.81	1.62	*	*	*
Hep 11	5.68	1.62	*	*	*
Hep 12	5.57	1.19	4.48	0.217	5th
Hep 13	8.36	2.24	5.35	0.288	14th
Hep 14	9.32	2.76	5.54	0.277	11th
Hep 15	9.59	2.58	5.75	0.279	13th
Hep 16	9.72	2.70	5.60	0.512	11th
Hep 17	9.25	0.20	2.59	0.214	7th

* It does not assume values.

Table S10. Excited State Analysis for Heptazine Structure 5. Excited states (eV), electron/hole pair (NTOs), oscillator strengths, and orbital contributions (%) for each analyzed state.

Excited State	Electron	Hole	Orbital	E(eV)	Oscillator strength
1st	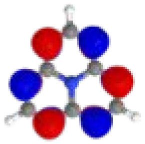	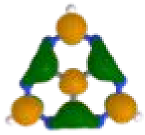	98% ($\pi \rightarrow \pi^*$)	3.06	0.000
2nd	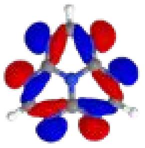	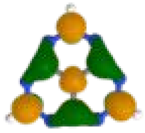	85% ($n \rightarrow \pi^*$)	4.01	0.000
3rd	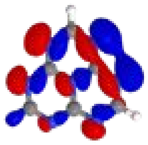	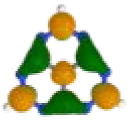	87% ($n \rightarrow \pi^*$)	4.05	0.000

4th	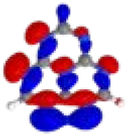	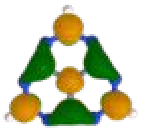	88% ($n\rightarrow\pi^*$)	4.05	0.000
5th	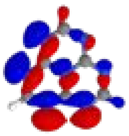	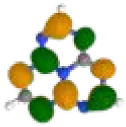	39% ($n\rightarrow\pi^*$)	5.00	0.001
6th	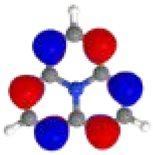	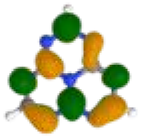	97% ($\pi\rightarrow\pi^*$)	5.08	0.239
7th	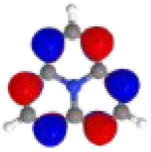	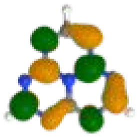	97% ($\pi\rightarrow\pi^*$)	5.08	0.239
8th	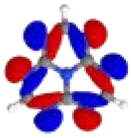	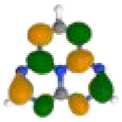	66% ($n\rightarrow\pi^*$)	5.12	0.000

Table S11. Excited State Analysis for Heptazine Structure 9. Excited states (eV), electron/hole pair (NTOs), oscillator strengths, and orbital contributions (%) for each analyzed state.

Excited State	Electron	Hole	Orbital	E(eV)	Oscillator strength
1st	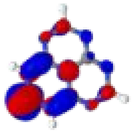	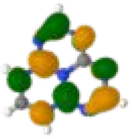	99% ($\pi\rightarrow\pi^*$)	1.644	0.013
2nd	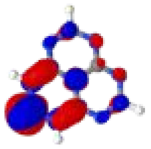	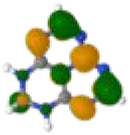	100% ($\pi\rightarrow\pi^*$)	2.177	0.039

3rd	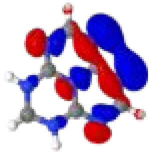	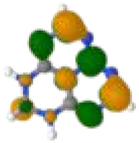	82% (*n*→*π**)	4.576	0.001
4th	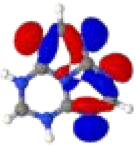	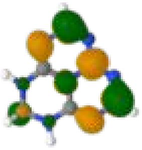	76% (*n*→*π**)	4.660	0.001
5th	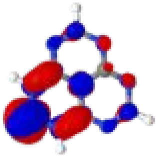	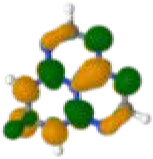	64% (*π*→*π**)	4.677	0.053
6th	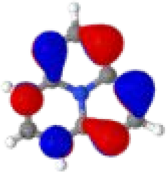	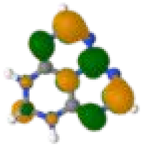	91% (*π*→*π**)	4.745	0.187
7th	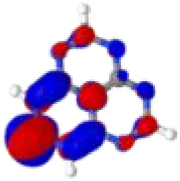	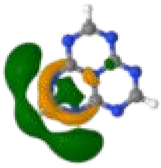	97% (*π*→*R**)	4.824	0.005
8th	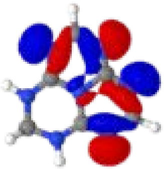	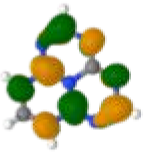	80% (*n*→*π**)	5.188	0.002

Table S12. Excited State Analysis for Heptazine Structure 10. Excited states (eV), electron/hole pair (NTOs), oscillator strengths, and orbital contributions (%) for each analyzed state.

Excited State	Electron	Hole	Orbital	E(eV)	Oscillator strength

1st	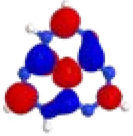	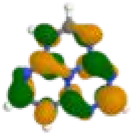	100% ($\pi\rightarrow\pi^*$)	1.62	0.046
2nd	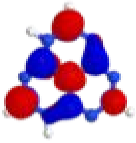	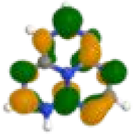	99% ($\pi\rightarrow\pi^*$)	2.01	0.055
3rd	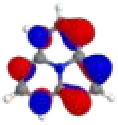	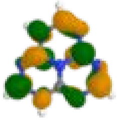	66% ($\pi\rightarrow\pi^*$)	4.53	0.019
4th	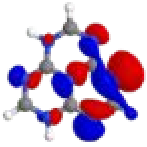	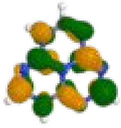	97% ($n\rightarrow\pi^*$)	4.59	0.002
5th	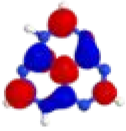	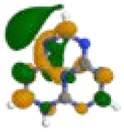	68% ($\pi\rightarrow R^*$)	4.92	0.008
6th	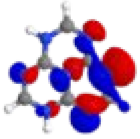	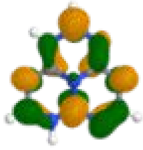	63% ($n\rightarrow\pi^*$)	5.01	0.003
7th	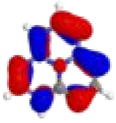	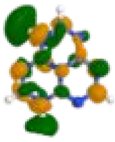	56% ($\pi\rightarrow R^*$)	5.11	0.003
8th	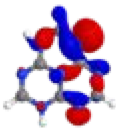	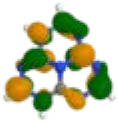	74% ($n\rightarrow\pi^*$)	5.27	0.027

Table S13. Excited State Analysis for Heptazine Structure 11. Excited states (eV), electron/hole pair (NTOs), oscillator strengths, and orbital contributions (%) for each analyzed state.

Excited State	Electron	Hole	Orbital	E(eV)	Oscillator strength
1st	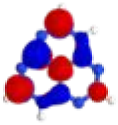	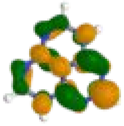	100%($\pi\rightarrow\pi^*$)	1.62	0.047
2nd	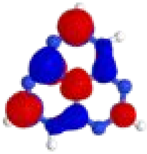	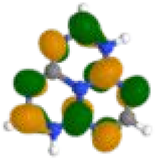	99% ($\pi\rightarrow\pi^*$)	1.89	0.034
3rd	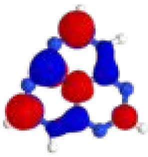	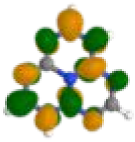	59% ($\pi\rightarrow\pi^*$)	4.49	0.028
4th	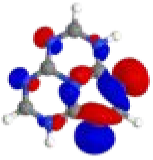	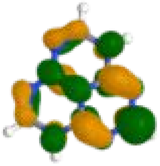	98% ($n\rightarrow\pi^*$)	4.99	0.003
5th	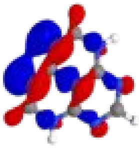	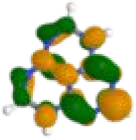	73% ($n\rightarrow\pi^*$)	5.07	0.002
6th	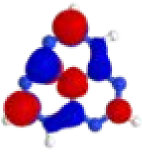	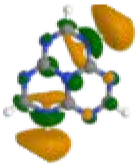	79% ($\pi\rightarrow R^*$)	5.12	0.001

Table S14. Excited State Analysis for Heptazine Structure 12. Excited states (eV), electron/hole pair (NTOs), oscillator strengths, and orbital contributions (%) for each analyzed state.

Excited State	Electron	Hole	Orbital	E(eV)	Oscillator strength
1st	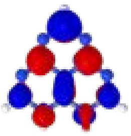	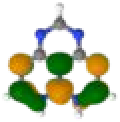	100% (*π→π**)	1.19	0.000
2nd	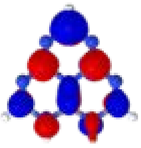	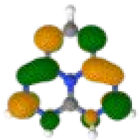	100% (*π→π**)	1.67	0.079
3rd	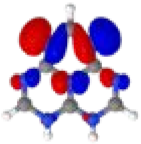	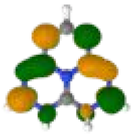	98% (*n→π**)	4.22	0.002
4th	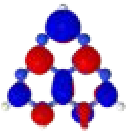	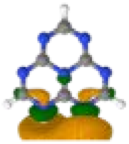	99% (*π→R**)	4.33	0.003
5th	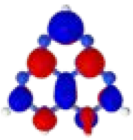	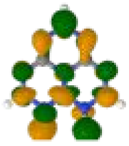	91% (*π→π**)	4.48	0.217
6th	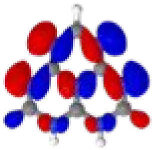	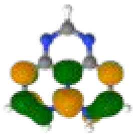	88%(*n→π**)	4.78	0.002

Table S15. Excited State Analysis for Heptazine Structure 14. Excited states (eV), electron/hole pair (NTOs), oscillator strengths, and orbital contributions (%) for each analyzed state.

Excited State	Electron	Hole	Orbital	E(eV)	Oscillator strength
1st	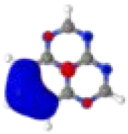	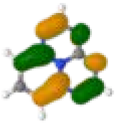	99% ($\pi\rightarrow\pi^*$)	2.244	0.041
2nd	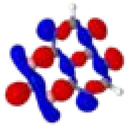	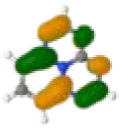	85% ($n\rightarrow\pi^*$)	3.242	0.000
3rd	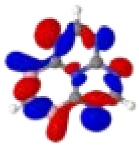	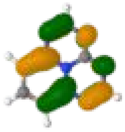	88% ($n\rightarrow\pi^*$)	3.345	0.003
4th	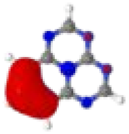	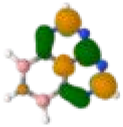	100% ($\pi\rightarrow\pi^*$)	3.392	0.042
5th	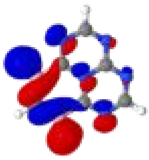	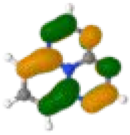	39% ($n\rightarrow\pi^*$)	3.637	0.000
6th	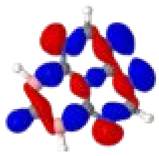	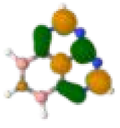	87% ($n\rightarrow\pi^*$)	4.032	0.001
7th	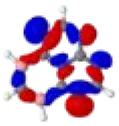	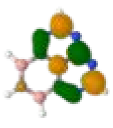	87% ($n\rightarrow\pi^*$)	4.153	0.000
8th	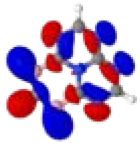	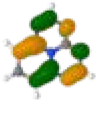	68% ($n\rightarrow\pi^*$)	4.755	0.000

9th	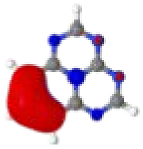	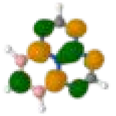	88% (*π*→*π**)	4.879	0.123
10th	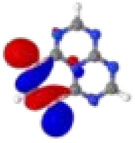	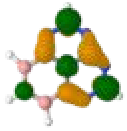	82% (*n*→*π**)	4.887	0.000
11th	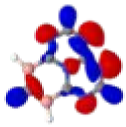	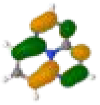	39% (*n*→*π**)	5.209	0.000
12th	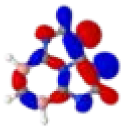	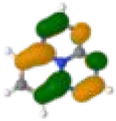	88% (*n*→*π**)	5.247	0.005
13th	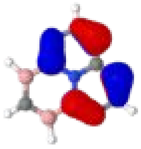	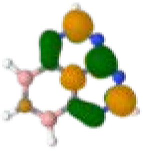	68% (*π*→*π**)	5.258	0.036
14th	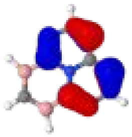	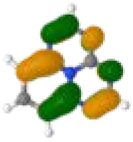	86% (*π*→*π**)	5.350	0.288
15th	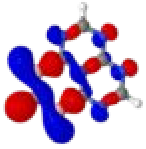	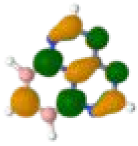	55% (*n*→*π**)	5.495	0.002

Table S16. Excited State Analysis for Heptazine Structure 14. Excited states (eV), electron/hole pair (NTOs), oscillator strengths, and orbital contributions (%) for each analyzed state.

Excited State	Electron	Hole	Orbital	E(eV)	Oscillator strength
1st	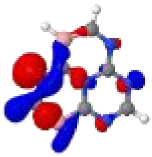	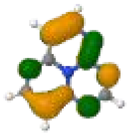	98% ($n\rightarrow\pi^*$)	2.76	0.001
2nd	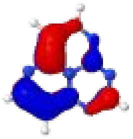	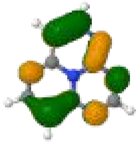	98% ($\pi\rightarrow\pi^*$)	2.97	0.213
3rd	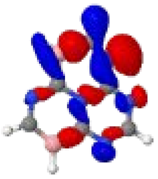	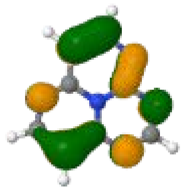	97% ($n\rightarrow\pi^*$)	2.98	0.000
4th	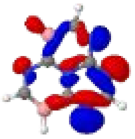	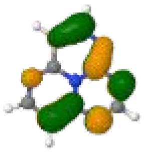	94% ($n\rightarrow\pi^*$)	3.58	0.002
5th	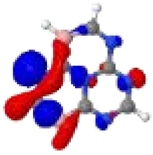	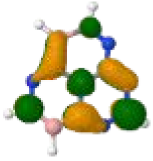	97%($n\rightarrow\pi^*$)	3.98	0.000
6th	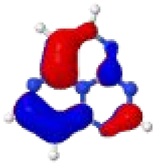	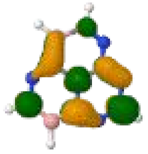	91%($\pi\rightarrow\pi^*$)	4.15	0.169
7th	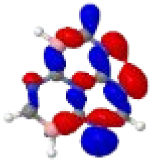	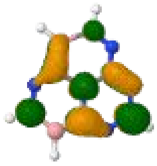	95% ($n\rightarrow\pi^*$)	4.42	0.000

8th	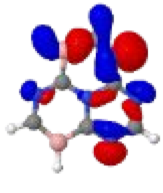	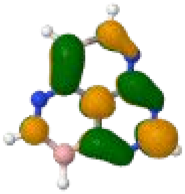	92% (*n*→*π**)	4.77	0.000
9th	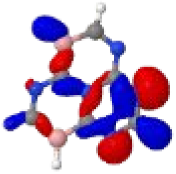	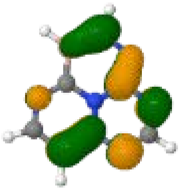	79% (*n*→*π**)	5.03	0.000
10th	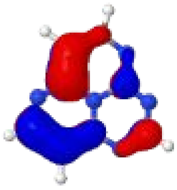	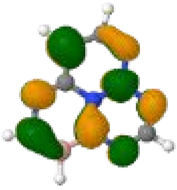	68%(*π*→*π**)	5.31	0.071
11th	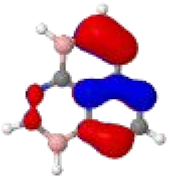	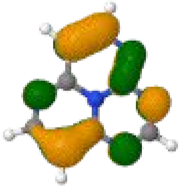	67% (*π*→*π**)	5.54	0.277
12th	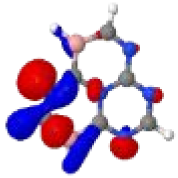	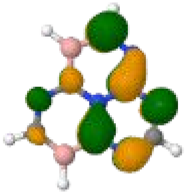	70% (*n*→*π**)	5.57	0.001

Table S17. Excited State Analysis for Heptazine Structure 15. Excited states (eV), electron/hole pair (NTOs), oscillator strengths, and orbital contributions (%) for each analyzed state.

Excited State	Electron	Hole	Orbital	E(eV)	Oscillator strength
1st	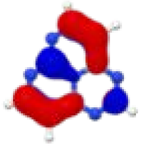	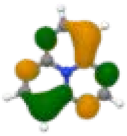	99% (*π*→*π**)	2.58	0.193

2nd	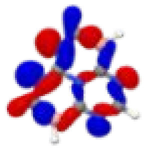	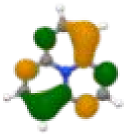	95%($n\rightarrow\pi^*$)	2.70	0.002
3rd	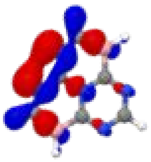	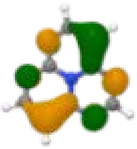	96%($n\rightarrow\pi^*$)	2.90	0.000
4th	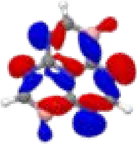	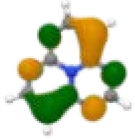	87% ($n\rightarrow\pi^*$)	3.42	0.000
5th	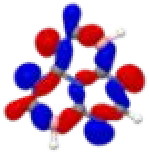	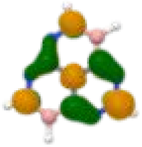	93% ($n\rightarrow\pi^*$)	3.91	0.000
6th	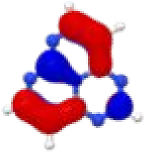	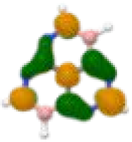	94%($\pi\rightarrow\pi^*$)	3.91	0.189
7th	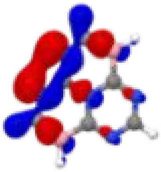	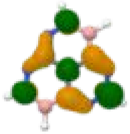	89% ($n\rightarrow\pi^*$)	4.19	0.000
8th	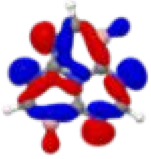	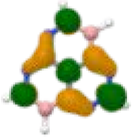	85% ($n\rightarrow\pi^*$)	4.62	0.000
9th	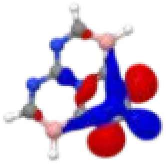	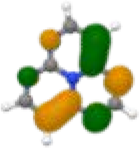	91% ($n\rightarrow\pi^*$)	4.93	0.000

10th	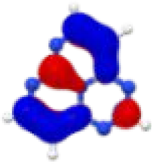	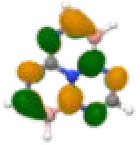	93%(π→π*)	5.07	0.200
11th	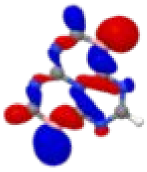	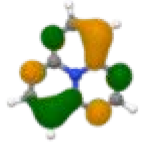	70% (n→π*)	5.41	0.003

Table S18. Excited State Analysis for Heptazine Structure 16. Excited states (eV), electron/hole pair (NTOs), oscillator strengths, and orbital contributions (%) for each analyzed state..

Excited State	Electron	Hole	Orbital	E(eV)	Oscillator strength
1st	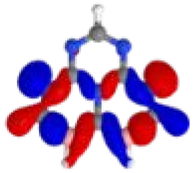	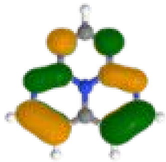	93% (n→π*)	2.70	0.000
2nd	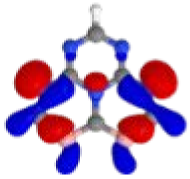	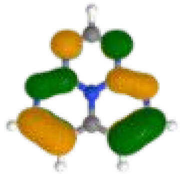	71%(n→π*)	3.31	0.000
3rd	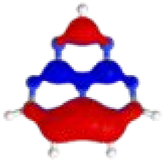	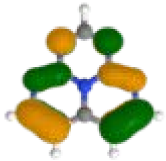	96% (π→π*)	3.43	0.171
4th	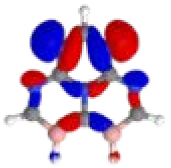	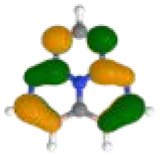	98% (n→π*)	3.83	0.003

5th	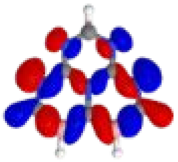	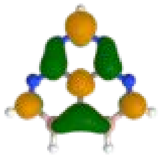	81% ($n\rightarrow\pi^*$)	4.20	0.048
6th	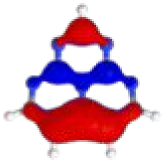	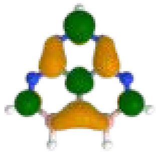	82% ($\pi\rightarrow\pi^*$)	4.45	0.000
7th	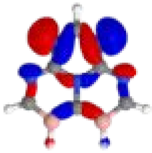	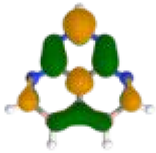	95% ($n\rightarrow\pi^*$)	4.64	0.000
8th	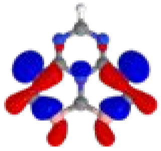	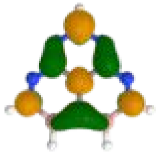	69% ($n\rightarrow\pi^*$)	4.80	0.000
9th	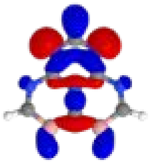	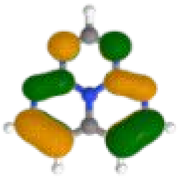	87% ($n\rightarrow\pi^*$)	5.21	0.000
10th	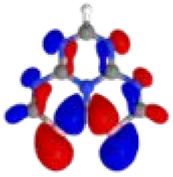	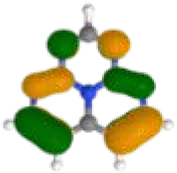	82% ($n\rightarrow\pi^*$)	5.29	0.001
11th	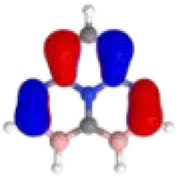	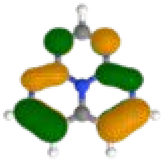	78% ($\pi\rightarrow\pi^*$)	5.60	0.512
12th	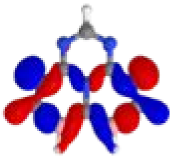	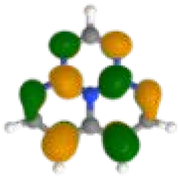	60% ($n\rightarrow\pi^*$)	5.66	0.003

Table S19. Excited State Analysis for Heptazine Structure 17. Excited states (eV), electron/hole pair (NTOs), oscillator strengths, and orbital contributions (%) for each analyzed state.

Excited State	Electron	Hole	Orbital	E(eV)	Oscillator strength
1st	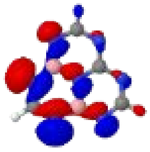	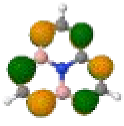	100% ($n \rightarrow \pi^*$)	0.20	0.000
2nd	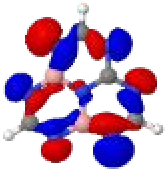	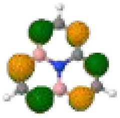	100% (n$\rightarrow \pi^*$)	0.82	0.001
3rd	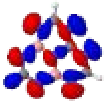	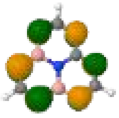	100% ($n \rightarrow \pi^*$)	0.83	0.000
4th	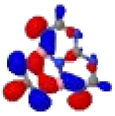	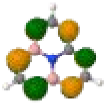	98% ($n \rightarrow \pi^*$)	1.83	0.000
5th	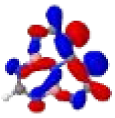	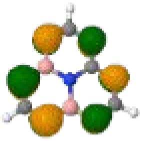	99% ($n \rightarrow \pi^*$)	2.38	0.000
6th	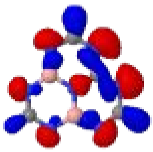	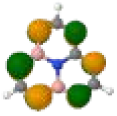	98% (n$\rightarrow \pi^*$)	2.42	0.000
7th	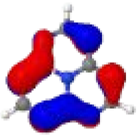	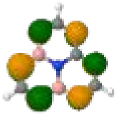	100% ($\pi \rightarrow \pi^*$)	2.59	0.214

8th	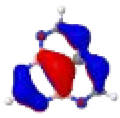	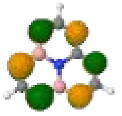	100% ($\pi \rightarrow \pi^*$)	2.62	0.042

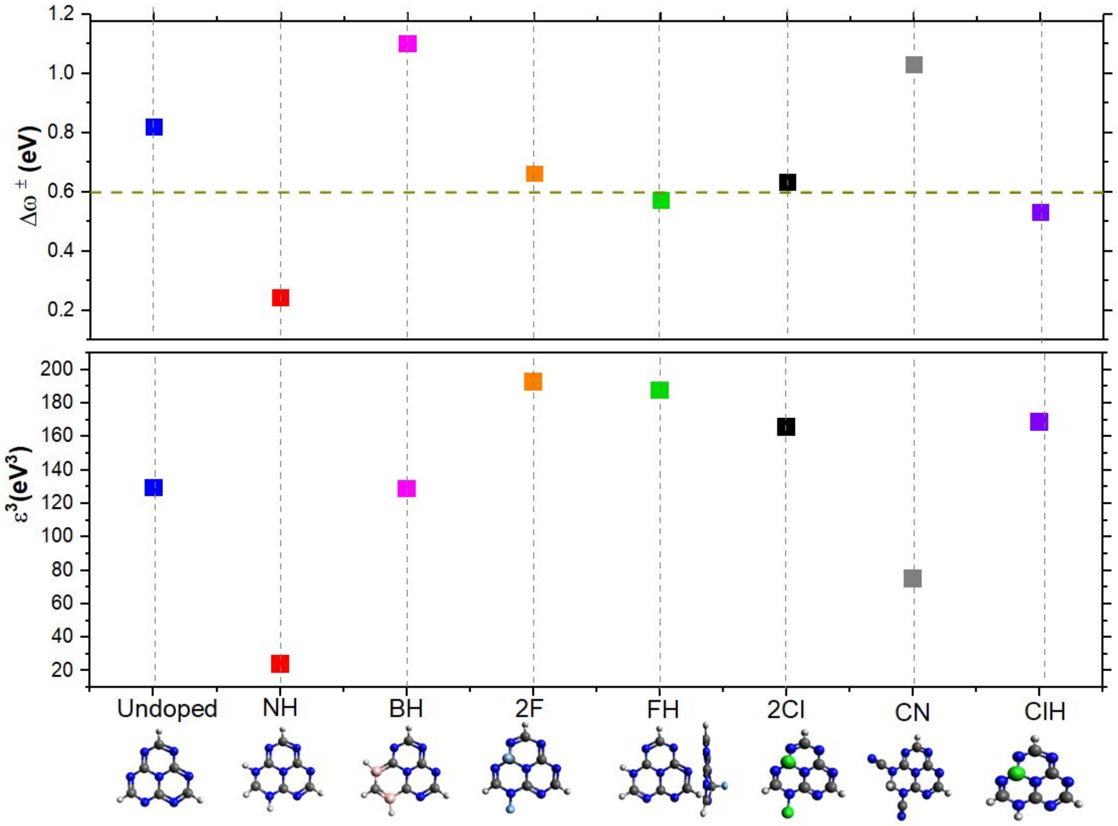

Figure S6. Schematic representation of the global stability index (ε^3) and the net electrophilicity index ($\Delta\omega^{\pm}$) used to evaluate the stability and reactivity of doped and undoped heptazines.

Table S20. First excited state (S_1, eV), bright state (eV), oscillator strength of the bright state, and the corresponding bright state (18 to 22 and 7).

	S_1 (eV)	Bright state (eV)	Oscillator strengh in Bright state	State	CT em S_1
18	1.85	3.54	0.410	10th	0.658
19	0.55	3.51	0.579	12th	0.468
20	0.72	2.62	0.293	6th	0.858
21	1.21	2.39	0.251	4th	0.553
22	1.37	3.09	0.165	6th	0.287
7	3.24	4.84	0.222	14th	0.141

Table S21. Excited State Analysis for Heptazine Structure 7. Excited states (eV), electron/hole pair (NTOs), oscillator strengths, orbital contributions (%) for each analyzed state and CT (charge transfer).

Excited State	Electron	Hole	Orbital	E (eV)	Oscillator strengh	CT
1st	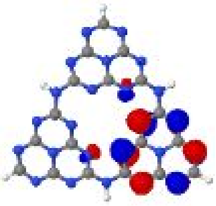	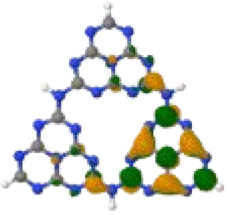	97% (π→π*)	3.24	0.000	0.141
2nd	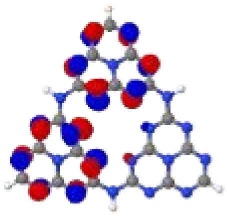	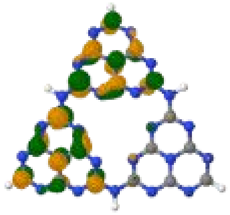	92% (n→π*)	3.26	0.000	0.138
3rd	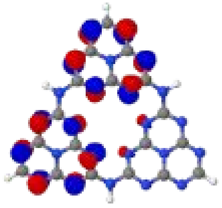	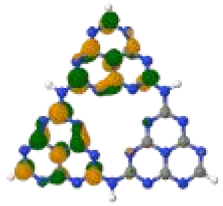	90% (n→π*)	3.36	0.001	0.096
4th	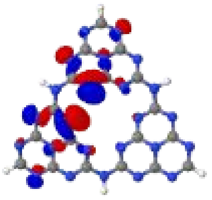	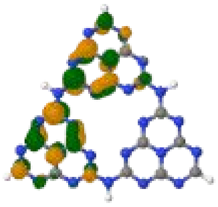	89% (π→π*)	3.92	0.007	0.254
5th	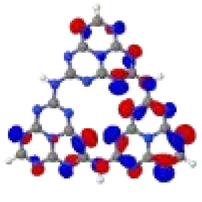	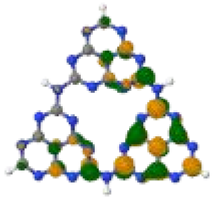	90% (n→π*)	4.09	0.001	0.210

6th	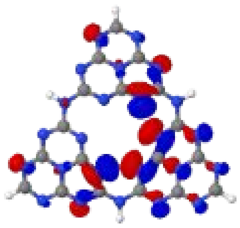	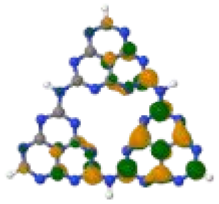	88% (π→π*)	4.11	0.005	0.198
7th	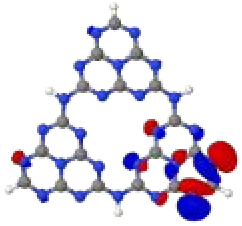	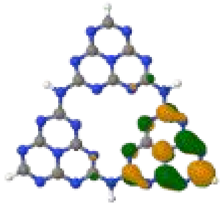	80% (n→π*)	4.23	0.000	0.039
8th	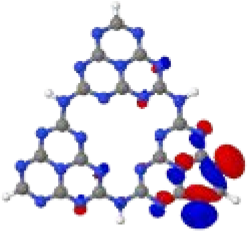	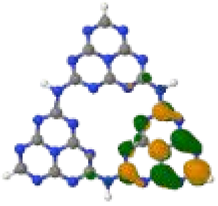	74% (n→π*)	4.26	0.000	0.070
9th	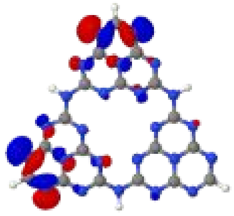	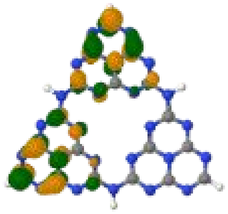	37% (n→π*)	4.36	0.000	0.062
10th	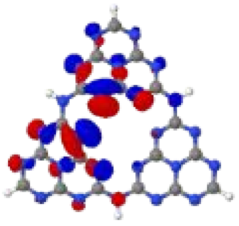	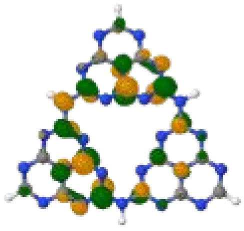	97% (n→π*)	4.42	0.000	0.179
11th	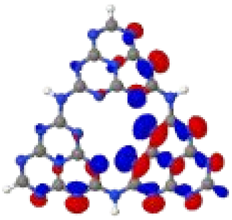	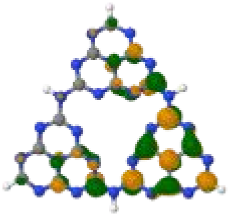	95% (n→π*)	4.47	0.003	0.141

12th	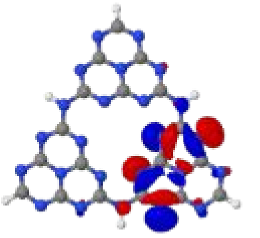	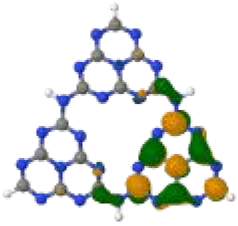	57% (π→π*)	4.52	0.000	0.205
13th	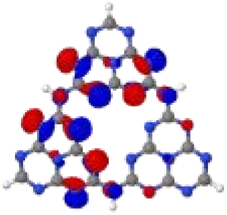	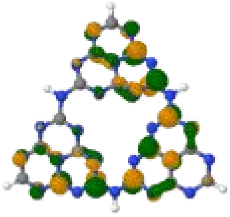	77% (n→π*)	4.84	0.005	0.392
14th	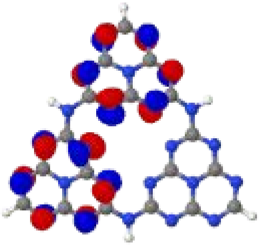	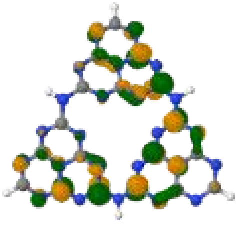	86% (n→π*)	4.84	0.222	0.402
15th	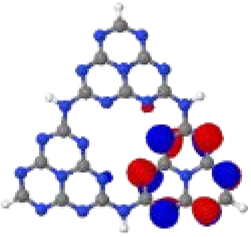	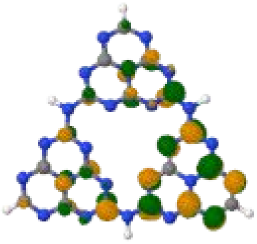	86% (n→π*)	4.87	0.039	0.270

Table S22. Excited State Analysis for Heptazine Structure 18. Excited states (eV), electron/hole pair (NTOs), oscillator strengths, orbital contributions (%) for each analyzed state and CT (charge transfer).

Excited State	Electron	Hole	Orbital	E (eV)	Oscillator strengh	CT
1st	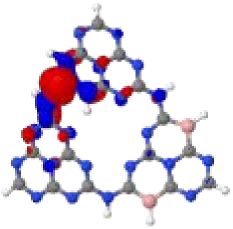	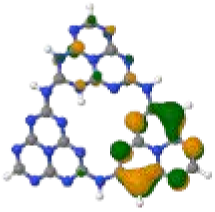	96% (*π*→*π**)	1.85	0.033	0.658

2nd	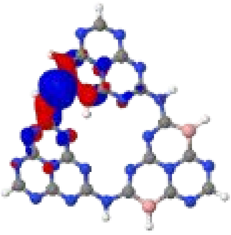	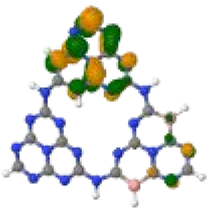	87% ($\pi \rightarrow \pi^*$)	1.98	0.004	0.249
3rd	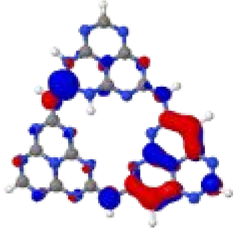	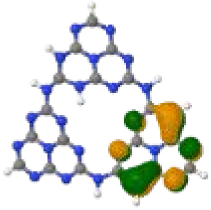	96% ($\pi \rightarrow \pi^*$)	2.07	0.102	0.326
4th	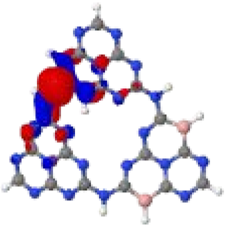	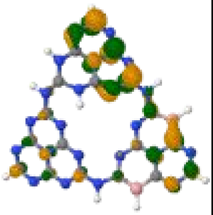	98% ($\pi \rightarrow \pi^*$)	2.44	0.082	0.429
5th	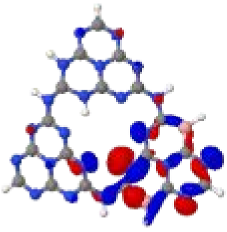	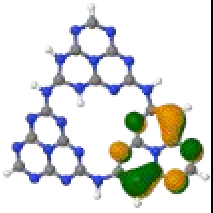	95% ($n \rightarrow \pi^*$)	2.75	0.002	0.109
6th	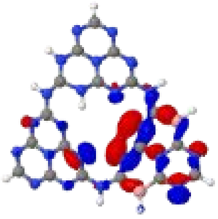	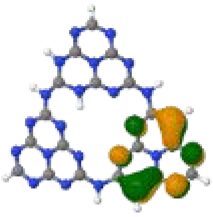	93% ($n \rightarrow \pi^*$)	3.05	0.002	0.162
7th	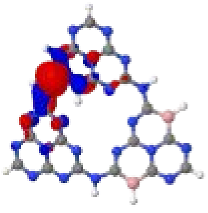	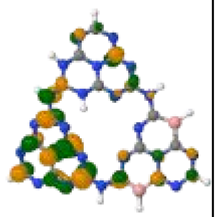	95% (n$\rightarrow \pi^*$)	3.19	0.132	0.614

8th	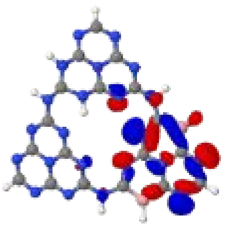	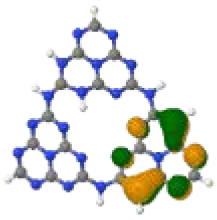	77% (*n→π**)	3.32	0.075	0.144
9th	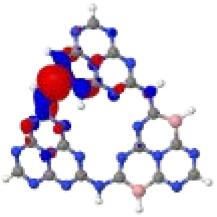	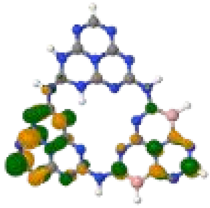	48% (*π→π**)	3.47	0.210	0.423
10th	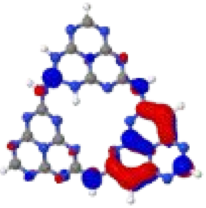	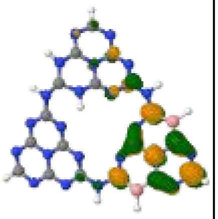	60% (*π→π**)	3.54	0.410	0.274
11th	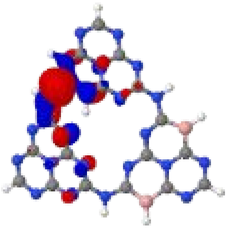	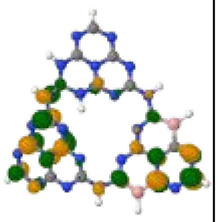	49% (*n→π**)	3.69	0.119	0.581

Table S23. Excited State Analysis for Heptazine Structure 19. Excited states (eV), electron/hole pair (NTOs), oscillator strengths, orbital contributions (%) for each analyzed state and CT (charge transfer).

Excited State	Electron	Hole	Orbital	E (eV)	Oscillator strengh	CT
1st	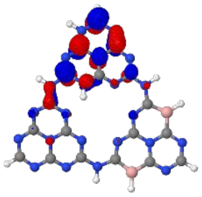	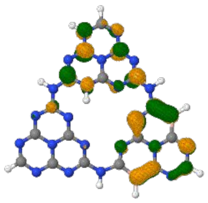	100% (*π→π**)	0.55	0.039	0.468

2nd	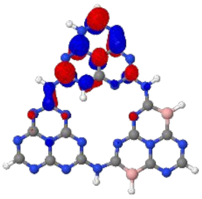	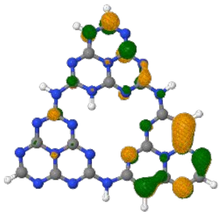	100% $(\pi\rightarrow\pi^*)$	0.88	0.084	0.635
3rd	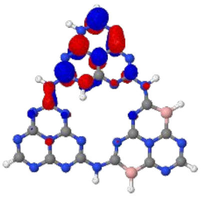	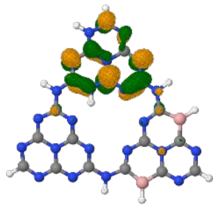	100% $(\pi\rightarrow\pi^*)$	1.26	0.049	0.247
4th	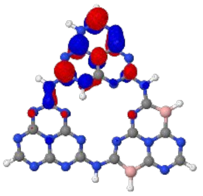	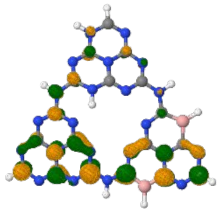	79% $(\pi\rightarrow\pi^*)$	1.95	0.004	0.620
5th	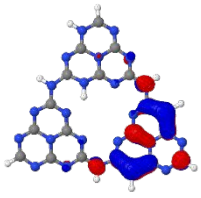	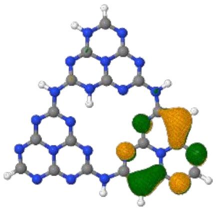	79% $(n\rightarrow\pi^*)$	2.09	0.149	0.313
6th	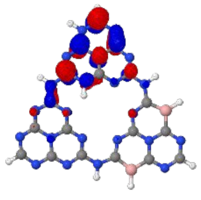	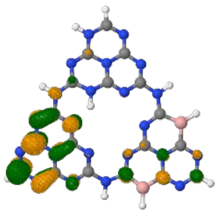	98% $(n\rightarrow\pi^*)$	2.55	0.156	0.752
7th	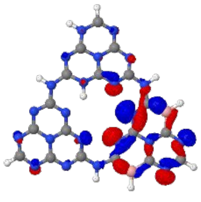	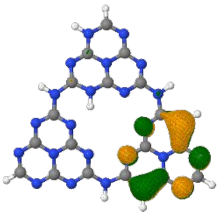	94% $(\mathrm{n}\rightarrow\pi^*)$	2.76	0.002	0.109
8th	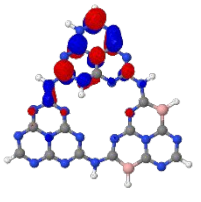	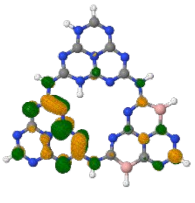	97% $(n\rightarrow\pi^*)$	2.79	0.176	0.770

9th	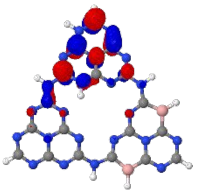	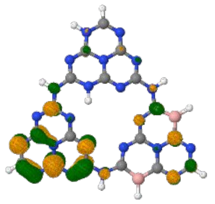	94% (*π→π**)	3.05	0.023	0.619
10th	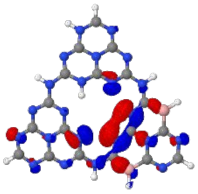	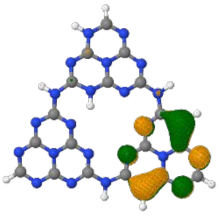	91% (*n→π**)	3.06	0.009	0.172
11th	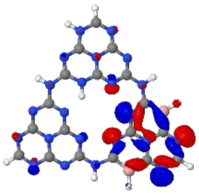	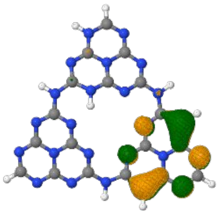	91% (n→*π**)	3.32	0.067	0.138
12th	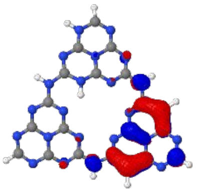	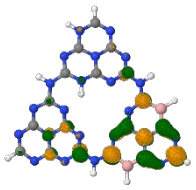	97% (*π→π**)	3.52	0.580	0.241
13th	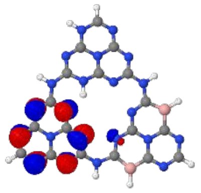	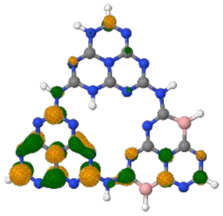	91% (*π→π**)	3.65	0.001	0.188

Table S24. Excited State Analysis for Heptazine Structure 20. Excited states (eV), electron/hole pair (NTOs), oscillator strengths, orbital contributions (%) for each analyzed state and CT (charge transfer).

Excited State	Electron	Hole	Orbital	E (eV)	Oscillator strengh	CT

1st	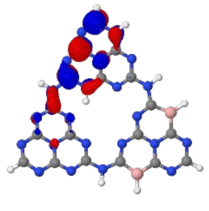	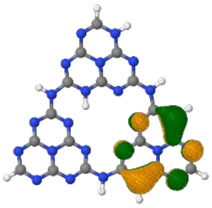	100% ($\pi \rightarrow \pi^*$)	0.72	0.027	0.858
2nd	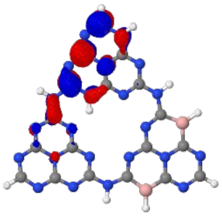	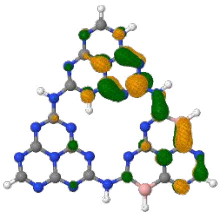	100% ($\pi \rightarrow \pi^*$)	1.29	0.009	0.479
3rd	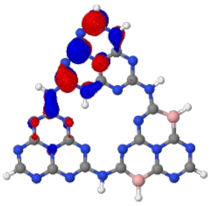	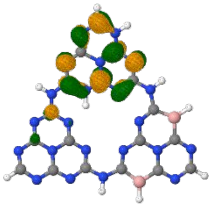	100% ($\pi \rightarrow \pi^*$)	1.35	0.088	0.301
4th	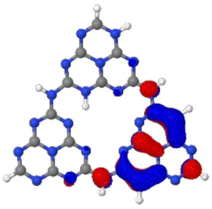	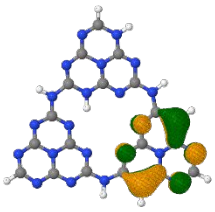	86% ($\pi \rightarrow \pi^*$)	2.02	0.045	0.204
5th	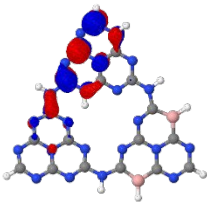	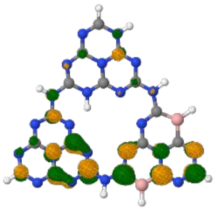	88% ($n \rightarrow \pi^*$)	2.18	0.178	0.658
6th	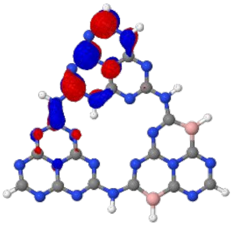	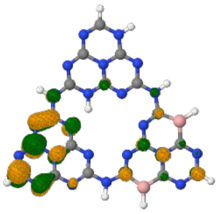	97% ($n \rightarrow \pi^*$)	2.62	0.293	0.619

7th	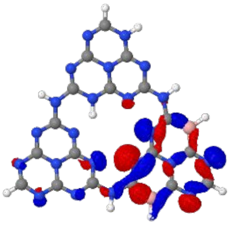	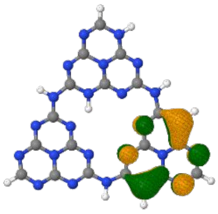	95% (n→π*)	2.73	0.002	0.097
8th	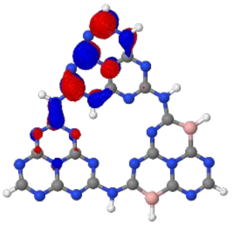	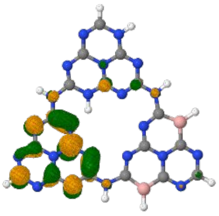	98% (*n*→π*)	2.88	0.121	0.768

Table S25. Excited State Analysis for Heptazine Structure 21. Excited states (eV), electron/hole pair characteristics from Natural Transition Orbitals (NTOs), oscillator strengths, and orbital contributions (%) for each analyzed state.

Excited State	Electron	Hole	Orbital	E (eV)	Oscillator strengh	CT
1st	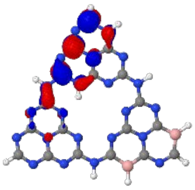	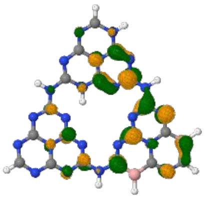	100% (π→π*)	1.21	0.021	0.553
2nd	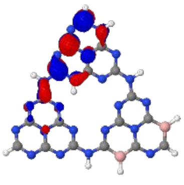	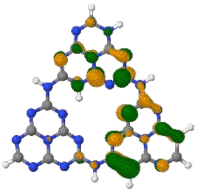	100% (π→π*)	1.29	0.048	0.601
3rd	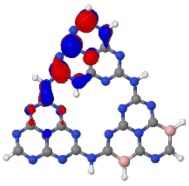	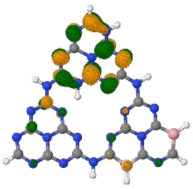	100% (π→π*)	1.34	0.059	0.262

4th	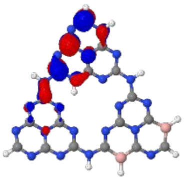	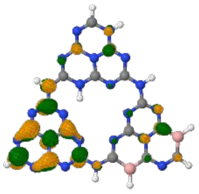	96% (*π*→*π**)	2.39	0.251	0.689
5th	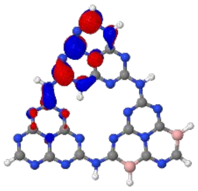	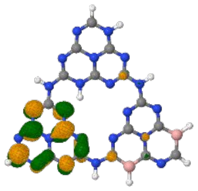	96% (*π*→*π**)	2.78	0.009	0.765
6th	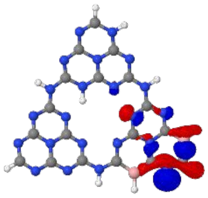	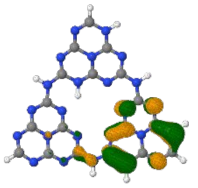	98% (*n*→*π**)	2.83	0.001	0.032
7th	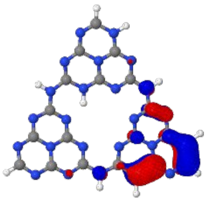	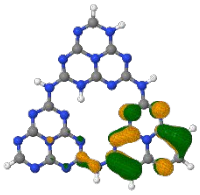	82% (n→*π**)	2.88	0.214	0.223
8th	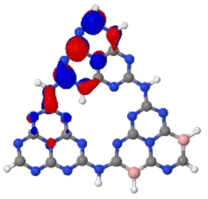	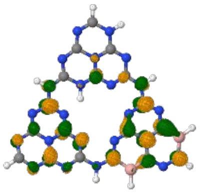	77% (*π*→*π**)	2.99	0.257	0.636
9th	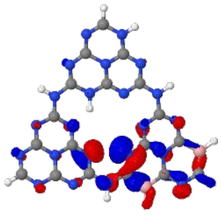	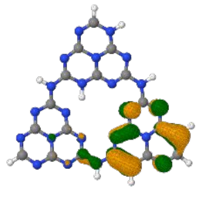	86% (n→*π**)	3.05	0.121	0.258
10th	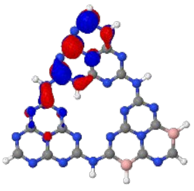	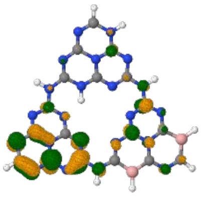	97% (*π*→*π**)	3.28	0.038	0.760

Table S26. Excited State Analysis for Heptazine Structure 22. Excited states (eV), electron/hole pair characteristics from Natural Transition Orbitals (NTOs), oscillator strengths, and orbital contributions (%) for each analyzed state.

Excited State	Electron	Hole	Orbital	E (eV)	Oscillator strengh	CT
1st	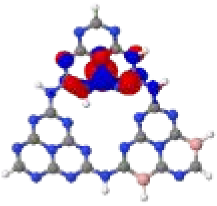	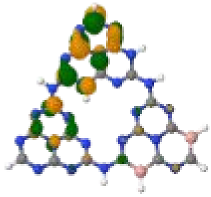	100% ($\pi \rightarrow \pi^*$)	1.37	0.013	0.287
2nd	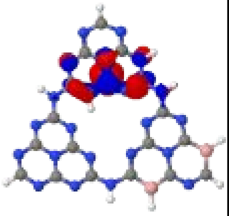	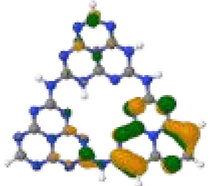	99% ($\pi \rightarrow \pi^*$)	1.63	0.003	0.810
3rd	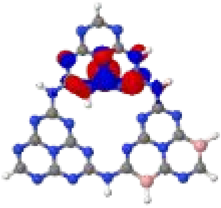	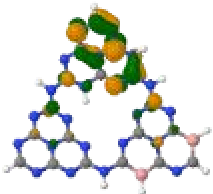	98% ($\pi \rightarrow \pi^*$)	1.96	0.035	0.234
4th	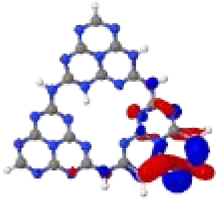	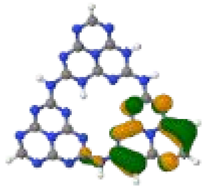	85% ($n \rightarrow \pi^*$)	2.77	0.101	0.130
5th	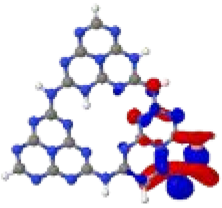	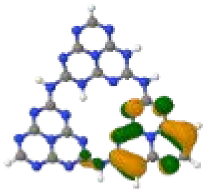	79% ($n \rightarrow \pi^*$)	2.78	0.067	0.168

6th	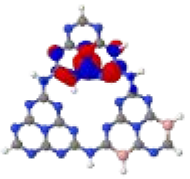	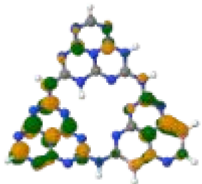	72% $(\pi\rightarrow\pi^*)$	2.93	0.165	0.583
7th	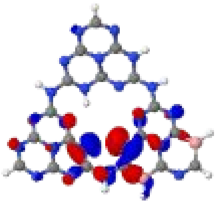	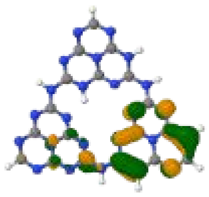	90% (n$\rightarrow\pi^*$)	3.09	0.023	0.233
8th	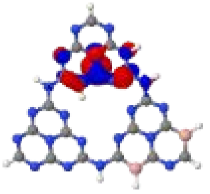	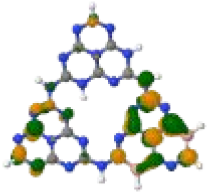	94% $(n\rightarrow\pi^*)$	3.18	0.138	0.788
9th	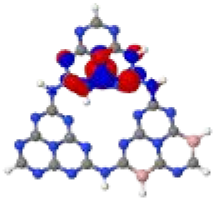	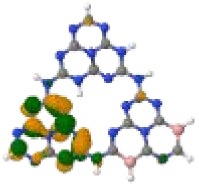	91% $(n\rightarrow\pi^*)$	3.37	0.010	0.847

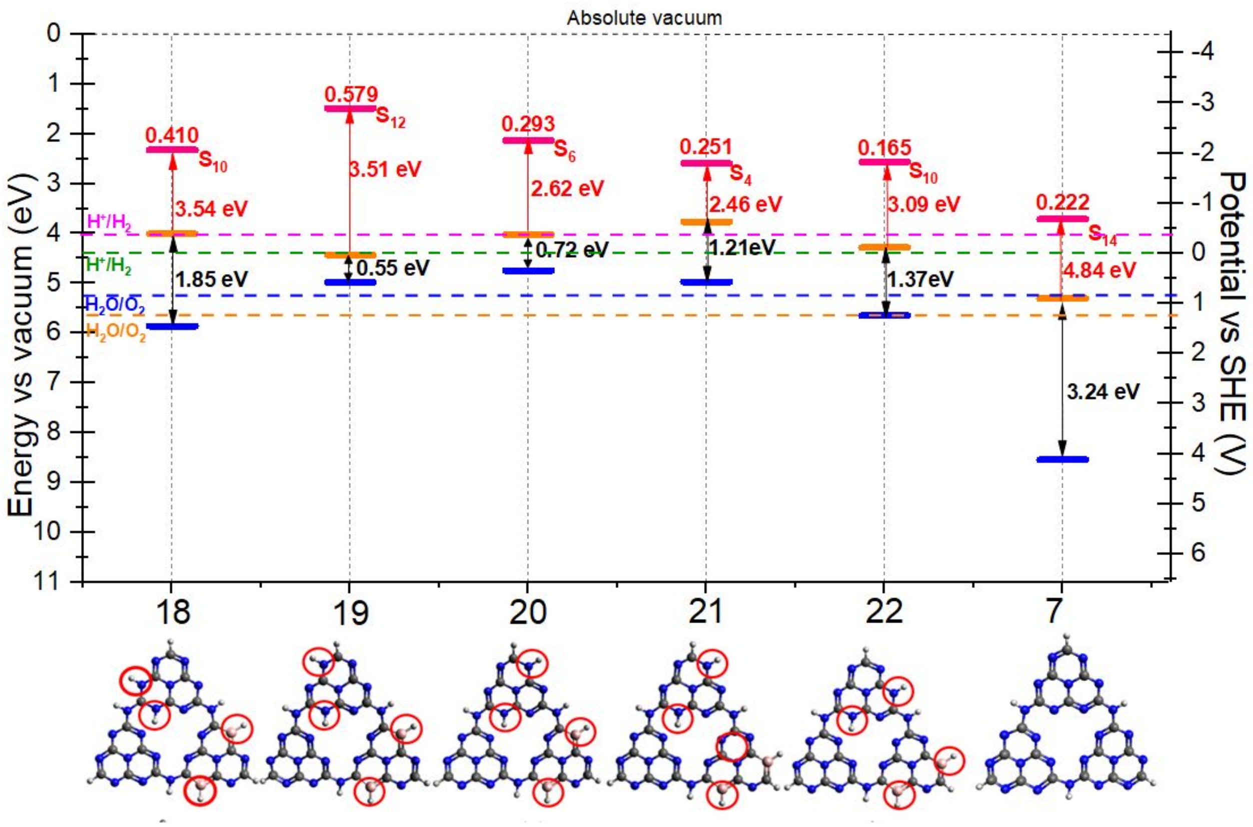

Figure S7. Thermodynamic criteria for THZ doped structures. The blue level represents the ionization energy, the orange level corresponds to the first excited state, and the red level represents the excited state with the highest oscillator strength (bright state), with this oscillator strength exceeding the red level. The left vertical axes represent energy versus vacuum in eV and the standard electrochemical potential (SHE).

Cartesian Coordinates of all studied structures (in Å), given by their image and numbering.

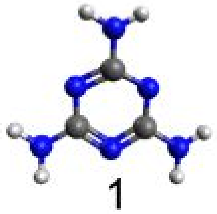

1

C -0.29895 1.25979 0.00029

N -1.31731 0.39388 0.00004

C -0.94159 -0.88899 -0.00019

C 1.24085 -0.37110 -0.00038

N 0.99977 0.94371 0.00003

H -2.88963 -1.51632 0.00086

N 0.31753 -1.33776 -0.00020

N -0.61161 2.57650 0.00029

N -1.92599 -1.81768 -0.00009

N 2.53744 -0.75857 -0.00034

H -1.68136 -2.79722 0.00064

H 2.75889 -1.74355 0.00160

H 3.26305 -0.05659 0.00186

H -1.58234 2.85417 -0.00072

H 0.13071 3.26076 -0.00072

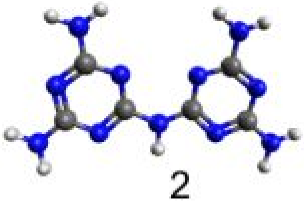

2

C -3.47232 -0.90798 0.00006

N -3.78868 0.39075 0.00004

C -2.73220 1.20621 -0.00018

C -1.26657 -0.47972 -0.00009

N -2.23213 -1.40585 -0.00001

H -3.92507 2.86969 0.00106

N -1.44507 0.83022 -0.00019

N -4.49348 -1.79440 0.00017

N -2.97207 2.53588 -0.00049

N 0.00000 -1.02666 -0.00014

H -2.18891 3.17360 0.00088

H -5.44400 -1.45349 0.00059

H -4.29015 -2.78351 0.00061

N 4.49347 -1.79441 -0.00012

H 4.29013 -2.78351 0.00012

H 5.44400 -1.45351 0.00028

C 3.47233 -0.90797 -0.00011

N 2.23212 -1.40585 -0.00013

N 3.78868 0.39074 0.00008

C 1.26658 -0.47972 -0.00003

C 2.73220 1.20622 0.00020

N 1.44507 0.83022 0.00013

N 2.97207 2.53588 0.00014

H 3.92508 2.86968 0.00057

H 2.18892 3.17361 0.00054

H 0.00001 -2.04037 -0.00015

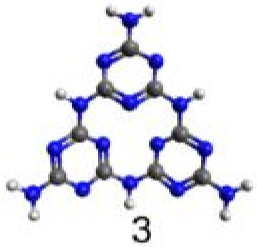

3

C -0.04938 -2.34847 0.00276

N -0.70684 -3.43651 -0.38430

C -2.03664 -3.34652 -0.20681

C -1.90969 -1.22575 0.49861

N -0.59607 -1.29318 0.58172

H -2.30461 -5.28166 -0.80438

N -2.70351 -2.23552 0.14424

N 1.32025 -2.29564 -0.20924

N -2.76645 -4.45860 -0.44343

N -2.52252 0.00018 0.74606

H -3.77467 -4.40162 -0.41062

H -3.53004 0.00025 0.62976

H 1.82109 -3.16800 -0.32459

H -3.77405 4.40196 -0.41121

C -1.90951 1.22601 0.49859

N -2.70317 2.23589 0.14419

C -2.03615 3.34680 -0.20681

N -2.76579 4.45902 -0.44337

N -0.70634 3.43660 -0.38428

N -0.59588 1.29328 0.58180

C -0.04903 2.34849 0.00283

N 1.32060 2.29548 -0.20915

C 2.05430 -1.11549 -0.21255

C 2.05448 1.11522 -0.21251

N 1.40598 -0.00008 -0.50400

N 3.35197 -1.19378 0.06733

N 3.35215 1.19330 0.06739

C 3.96596 -0.00029 0.10977

H -2.30387 5.28177 -0.80493

N 5.31065 -0.00040 0.24658

H 5.78661 -0.87884 0.39826

H 5.78674 0.87797 0.39830

H 1.82157 3.16777 -0.32446

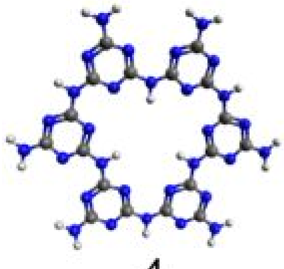

4

N -4.06194 -3.53586 0.00044

C -5.34463 -3.14604 0.00018

H -7.25662 -3.87565 0.00121

N -1.83873 -2.77399 -0.00042

C	-3.19654	-2.53859	-0.00034
N	-5.77191	-1.88203	-0.00068
C	-4.79254	-0.97163	-0.00145
N	-3.49626	-1.23115	-0.00140
H	-1.27808	-1.92816	-0.00204
N	-1.35037	-7.38708	0.00968
H	-0.74197	-8.19321	0.01056
N	0.51546	-6.04873	0.00426
C	-0.81513	-6.14794	0.00620
H	-2.35568	-7.48550	0.01091
C	0.97213	-4.79193	0.00089
N	-1.67372	-5.11846	0.00500
C	-1.09278	-3.93268	0.00144
N	0.22823	-3.69905	-0.00075
N	0.68141	3.64303	0.00193
C	-0.60068	4.03766	0.00161
N	2.91180	4.39381	0.00296
C	1.55443	4.63582	0.00250
N	-1.03089	5.28600	0.00182
C	-0.05186	6.20171	0.00242
N	1.25634	5.93928	0.00280
H	3.47193	5.23902	0.00329
N	-0.43012	7.49741	0.00279
H	-1.41563	7.71895	0.00236
H	0.27312	8.22229	0.00311
N	3.32326	-0.20542	-0.00014
N	5.27060	1.11078	-0.00002
C	3.95326	1.02022	0.00033
H	2.31046	-0.14283	0.00089
N	7.07261	2.52641	0.00038
C	5.73192	2.36954	0.00069
H	7.66077	1.70518	0.00080
N	3.09024	2.04708	0.00134
C	3.66380	3.23823	0.00202

N   4.98041   3.47201   0.00176

H   7.46587   3.45664   0.00212

N   2.34898 -4.71898 -0.00103

H   2.80075 -5.62671 -0.00082

N   4.51543 -4.05867 -0.00439

C   3.23758 -3.66481 -0.00201

N   6.70807 -3.37741 -0.00763

C   5.39683 -3.05692 -0.00528

H   6.98377 -4.34901 -0.00947

N   2.81487 -2.41217 -0.00057

C   3.79766 -1.49923 -0.00158

N   5.09383 -1.75105 -0.00393

H   7.39288 -2.63485 -0.00910

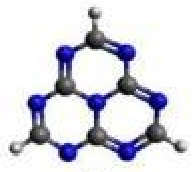

5

C 1.23453 0.66673 0.00006

C 0.07414 2.60968 -0.00001

C -1.19468 0.73569 -0.00010

N 0.00004 -0.00005 0.00003

H 0.10542 3.70632 -0.00013

N 1.24804 1.99482 0.00005

N -1.13271 2.06255 -0.00009

N -2.35160 0.08348 0.00001

C -0.03980 -1.40234 -0.00015

N -1.21989 -2.01222 -0.00018

N 1.10354 -2.07831 0.00002

N 2.35259 -0.05030 0.00003

C 2.22296 -1.36906 0.00030

C -2.29714 -1.24068 0.00019

H -3.26253 -1.76185 -0.00038

H 3.15703 -1.94445 -0.00032

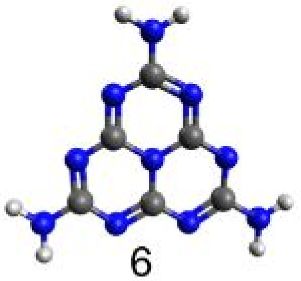

6

C	-1.18188	0.76506	0.00003
C	0.13344	2.61553	0.00040
C	1.25341	0.64065	0.00006
N	-0.00009	0.00003	0.00001
N	-1.09489	2.08300	0.00021
N	1.30108	1.96054	0.00024
N	2.35147	-0.09324	0.00004
C	-0.07164	-1.40568	-0.00012
N	1.04737	-2.10720	-0.00015
N	-1.25631	-1.98958	-0.00018
N	-2.34888	0.14658	-0.00002
C	-2.33195	-1.19236	-0.00010
C	2.19851	-1.42344	-0.00004
N	3.32677	-2.15351	-0.00014
N	0.20193	3.95775	0.00018
N	-3.52849	-1.80418	-0.00026
H	-4.36970	-1.24350	-0.00025
H	-3.56651	-2.81446	-0.00037
H	4.22064	-1.68120	-0.00011
H	3.26213	-3.16240	-0.00026
H	1.10811	4.40595	0.00007
H	-0.65393	4.49587	0.00004

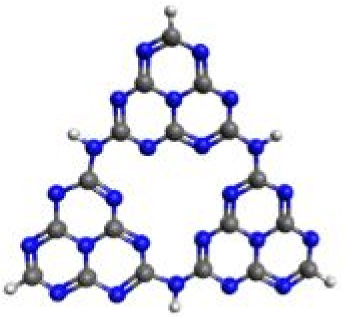

7

C 1.28495 -2.39207 -0.58028

C 3.20524 -1.22323 -0.68997

C 3.41527 -3.36087 0.03636

N 2.01668 -3.45208 -0.03108

N 4.09875 -4.43751 0.41722

C 1.34720 -4.60721 0.39783

N 2.07352 -5.65739 0.76663

C -0.58694 -3.50442 -0.00421

C 3.39272 -5.51413 0.73353

N 3.82015 0.01327 -0.77915

N -1.95271 -3.43542 0.18742

C 3.35377 5.53710 0.73525

C 3.39137 3.38436 0.03721

C 1.31462 4.61649 0.39789

C -0.61165 3.50058 -0.00545

C 3.19646 1.24562 -0.68985

C 1.26812 2.40126 -0.58104

N 4.06727 4.46543 0.41905

N 2.03355 5.67138 0.76758

N 1.99228 3.46607 -0.03110

N 3.98213 2.23741 -0.25371

N -0.04524 2.48398 -0.63757

N -0.00981 4.59947 0.44826

N 1.91406 1.32644 -0.98949

N -1.97718 3.42204 0.18537

C -2.69247 2.24117 0.23616

C -2.63843 -0.00902 0.41145

C -4.67942 1.20343 -0.07856

C -6.56141 -0.02305 -0.35381

C -2.67658 -2.25956 0.23742

C -4.67087 -1.23606 -0.07785

N -2.02373 1.14702 0.56914

N -3.99612 2.33648 -0.02534

N -3.99606 -0.01389 0.05941

N -2.01565 -1.16054 0.57007

N -5.98507 -1.21505 -0.27915

N -5.99356 1.17292 -0.28007

N -3.97941 -2.36435 -0.02400

H -2.46040 -4.30581 0.31012

H -2.49093 4.28880 0.30855

H 4.80296 0.01686 -0.51941

N 0.02280 -4.59934 0.44901

H 3.92987 6.42748 1.01700

H 3.97515 -6.40061 1.01464

H -7.64877 -0.02700 -0.50108

N -0.02775 -2.48402 -0.63660

N 1.92315 -1.31263 -0.98859

N 3.99801 -2.20998 -0.25510

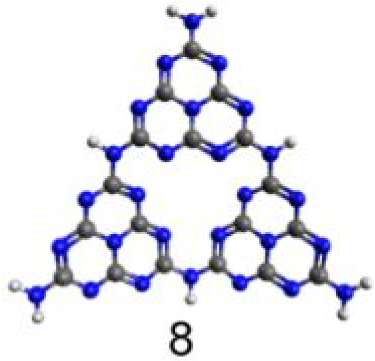

8

N 4.00131 -0.00011 -0.13794

N 2.01345 1.15273 -0.60340

N 3.99414 2.35260 -0.05326

N 5.99597 1.20197 0.14998

C	4.68956	-1.22047	-0.01853
N	5.99590	-1.20231	0.14996
N	3.99401	-2.35282	-0.05331
N	2.01339	-1.15283	-0.60343
C	2.68726	-2.24902	-0.28430
C	6.58911	-0.00018	0.21239
N	7.92159	-0.00022	0.36023
N	1.96631	3.42708	-0.21658
N	1.96611	-3.42718	-0.21665
H	8.41187	0.88202	0.42712
H	8.41182	-0.88250	0.42710
C	-3.39815	5.52607	-0.59560
C	-1.33950	4.60980	-0.32871
C	-3.39700	3.37096	0.11668
C	-3.16205	1.23635	0.85390
C	0.60622	3.50210	0.01775
C	-1.24618	2.40021	0.66830
N	-2.06330	5.65259	-0.67905
N	-4.09108	4.43217	-0.24387
N	-1.99339	3.45457	0.13352
N	-0.01297	4.59197	-0.42937
N	-1.86992	1.32539	1.11025
N	-3.96895	2.21599	0.43538
N	0.06819	2.48917	0.68159
N	-3.77577	0.00010	0.97789
N	-4.11818	6.61163	-0.91304
H	-5.12797	6.56258	-0.88078
H	-3.64038	7.45546	-1.20085
C	-3.16212	-1.23618	0.85391
C	-1.24631	-2.40013	0.66827
C	-3.39719	-3.37078	0.11670
C	-3.39846	-5.52589	-0.59556
C	0.60602	-3.50213	0.01771
C	-1.33976	-4.60972	-0.32871

N -1.86998 -1.32528 1.11021

N -3.96908 -2.21578 0.43541

N -1.99358 -3.45446 0.13352

N 0.06806 -2.48917 0.68154

N -2.06362 -5.65249 -0.67904

N -4.09133 -4.43196 -0.24382

N -0.01323 -4.59197 -0.42940

N -4.11856 -6.61142 -0.91297

H -3.64080 -7.45526 -1.20084

H -5.12834 -6.56230 -0.88074

H 2.47104 -4.29518 -0.35932

H -4.76375 0.00013 0.74145

H 2.47128 4.29506 -0.35926

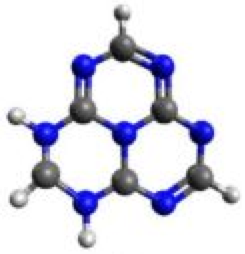

9

C 1.46802 -0.00007 0.04474

C 1.36765 2.26360 -0.02494

C -0.63406 1.22525 -0.01443

N 0.06641 0.00001 0.10590

H 1.91801 3.21197 -0.04585

N 2.11018 1.15869 0.01783

N 0.04633 2.36301 -0.06174

N -1.94134 1.16394 -0.03314

C -0.63418 -1.22519 -0.01443

N -1.94145 -1.16377 -0.03315

N 0.04612 -2.36301 -0.06174

N 2.11007 -1.15887 0.01781

C 1.36744 -2.26373 -0.02494

C -2.74801 0.00012 0.20125

H -3.60758 0.00016 -0.48375

H 1.91772 -3.21214 -0.04587

H -2.41168 2.06366 -0.04522

H -2.41187 -2.06345 -0.04515

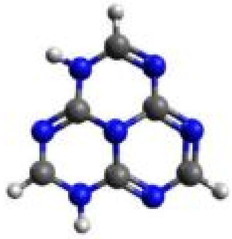

10

C -0.94357 -1.10603 -0.05539
C 0.84525 -2.50353 0.07446
C 1.33127 -0.32139 -0.10018
N -0.00441 -0.04098 -0.50883
H 1.21746 -3.53222 0.12868
N -0.45978 -2.32654 0.10739
N 1.77613 -1.52916 0.08121
N 2.15789 0.73186 0.01945
C -0.41359 1.28772 -0.03839
N 0.42023 2.27984 0.11482
N -1.74496 1.45626 0.05676
N -2.21546 -0.82949 0.06606
C -2.62314 0.42893 0.02431
C 1.70928 2.03481 0.04516
H 2.45334 2.82738 0.11837
H -3.68496 0.67753 0.06527
H 3.13145 0.50445 0.19604
H -2.05772 2.40726 0.23367

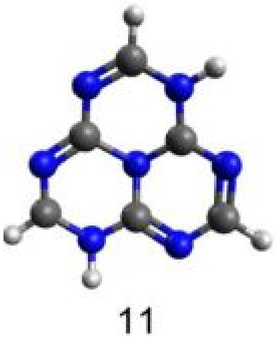

11

C -1.16480 0.72655 -0.08932
C -0.00001 2.63828 0.08245
C 1.16480 0.72655 -0.08932
N -0.00000 0.02359 -0.43860
H -0.00001 3.73134 0.14800
N -1.18568 2.03200 0.08417
N 1.18568 2.03200 0.08417
N 2.28246 0.01065 0.00564
C 0.00000 -1.48381 -0.00868
N 1.17122 -2.05797 0.10138
N -1.17122 -2.05797 0.10138
N -2.28246 0.01064 0.00564

C -2.28257 -1.38798 0.02776
C 2.28258 -1.38798 0.02778
H 3.25867 -1.87170 0.08011
H -3.25866 -1.87171 0.08009
H 3.13089 0.53595 0.19062
H -3.13089 0.53593 0.19064

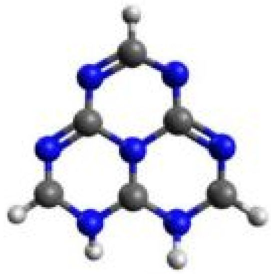

12

C 0.79517 -1.24791 0.00295

C -1.20868 -2.37579 -0.01100

C -1.28941 0.00009 0.00027

N 0.00025 -0.00002 0.00018

H -1.79981 -3.29773 -0.00006

N 0.05853 -2.40194 0.01627

N -1.99822 -1.17547 -0.08563

N -1.99798 1.17588 0.08632

C 0.79542 1.24783 -0.00298

N 0.05892 2.40193 -0.01713

N 2.07847 1.18017 -0.00690

N 2.07827 -1.18051 0.00706

C 2.71712 -0.00026 0.00025

C -1.20828 2.37595 0.01034

H -1.79927 3.29798 -0.00146

H 3.80782 -0.00033 0.00035

H -2.88217 1.18228 -0.41973

H -2.88219 -1.18194 0.42078

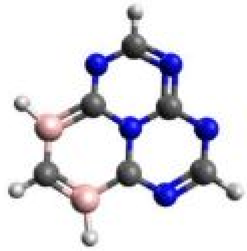

13

C 1.41035 0.00001 -0.00001

C 1.37196 -2.24815 -0.00012
C -0.67699 -1.22183 0.00009
N 0.03249 0.00000 0.00001
H 1.91373 -3.20144 -0.00024
N 2.08940 -1.15251 -0.00010
N 0.02846 -2.32664 0.00002
C -0.67702 1.22181 -0.00005
N 0.02841 2.32664 0.00000
N 2.08937 1.15255 0.00007
C 1.37192 2.24818 0.00011
C -3.03200 -0.00003 -0.00006
H -4.12968 -0.00005 -0.00008
H 1.91366 3.20148 0.00022
B -2.27725 -1.27336 0.00021
B -2.27727 1.27332 -0.00018
H -2.70563 -2.40913 0.00049
H -2.70567 2.40909 -0.00035

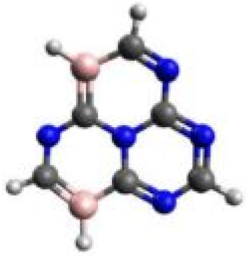

14

C 1.36021 0.18737 -0.00001
C 1.02182 2.43761 0.00007
C -0.84610 1.13722 -0.00005
N -0.03310 0.00074 -0.00001
H 1.46421 3.43985 0.00016
N 1.85793 1.40661 0.00005
N -0.29630 2.34801 -0.00000
C -0.58666 -1.28537 0.00004
N 2.25462 -0.83805 -0.00007
C 1.85202 -2.07408 -0.00006
C -2.80328 -0.50256 0.00000
H -3.84885 -0.84317 0.00004
H 2.67270 -2.80810 -0.00013
B -2.38149 0.95497 -0.00010

H -3.10457 1.92096 -0.00017
H -0.05177 -3.60715 0.00019
N -1.92143 -1.47814 0.00006
B 0.35113 -2.47052 0.00007

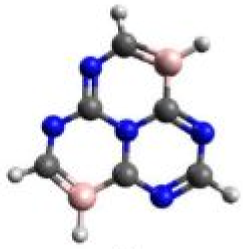

15

C -0.76151 -1.20825 -0.00001
C -2.69913 0.00036 -0.00002
C -0.76119 1.20844 0.00002
N -0.04460 0.00000 0.00000
H -3.79443 0.00050 -0.00004
C 1.34486 -0.00018 -0.00001
C 1.52072 -2.33795 0.00003
C 1.52134 2.33756 -0.00003
H 2.25535 3.15649 -0.00006
H 2.25451 -3.15707 0.00005
B 0.01419 2.54383 0.00004
H -0.57796 3.59452 0.00011
H -0.57891 -3.59437 0.00000
N 2.05908 1.14110 -0.00003
N 2.05878 -1.14163 0.00001
N -2.08590 -1.17585 -0.00002
N -2.08558 1.17640 0.00002
B 0.01352 -2.54383 0.00000

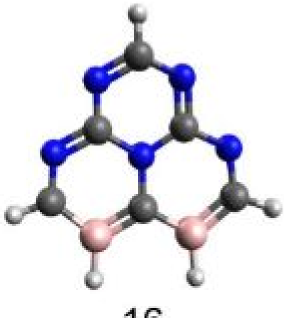

16

C -1.19377 -0.65626 -0.00002
C -2.55440 1.22308 -0.00006
C 0.00000 1.47405 0.00006
N 0.00000 0.07246 0.00001
H -3.60295 1.56086 -0.00014
C 1.19377 -0.65626 -0.00002
C 0.00000 -2.58421 0.00006
C 2.55440 1.22308 -0.00006
H 3.60295 1.56086 -0.00015

H 0.00000 -3.67990 0.00012
B 1.33016 2.18051 0.00007
H 1.42427 3.38613 0.00017
H -1.42427 3.38613 0.00015
N 2.42331 -0.06952 -0.00008
N 1.17507 -1.97814 0.00002
N -1.17507 -1.97814 0.00002
B -1.33017 2.18051 0.00006
N -2.42331 -0.06952 -0.00008

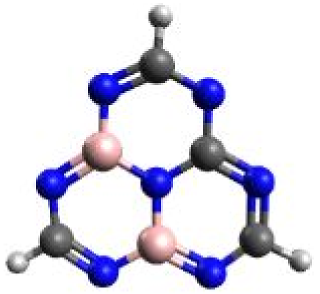

17

C -1.36165 0.00023 0.00095
C -1.35736 -2.30011 0.00166
B 0.72086 -1.25246 -0.00613
N -0.00732 0.00005 -0.00538
H -1.98204 -3.20507 0.00511
N -2.05151 -1.15285 0.00331
N -0.04098 -2.45114 -0.00241
N 2.15725 -1.19438 -0.00319
B 0.72120 1.25223 -0.00617
N 2.15782 1.19379 -0.00274
N -0.04035 2.45134 -0.00276
N -2.05120 1.15322 0.00341
C -1.35664 2.30055 0.00195
C 2.74851 -0.00046 0.01097
H -1.98138 3.20552 0.00444
H 3.85016 -0.00086 0.02697

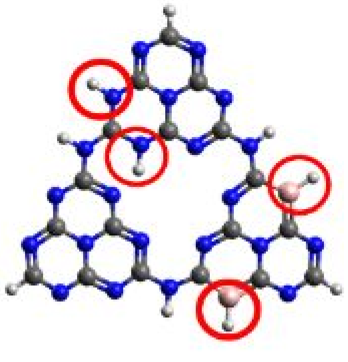

18

C 1.81551 -1.94503 0.53793
C 0.24947 -3.30551 -0.35355
C 2.40018 -3.99760 -0.61023
N 2.80228 -2.80415 0.01234
N 3.34664 -4.86904 -0.97960

C	4.15087	-2.57654	0.28496
N	5.05263	-3.48566	-0.04319
C	3.56724	-0.44640	1.43132
C	4.59353	-4.56810	-0.67504
N	-1.05619	-3.57852	-0.63920
N	3.77169	0.77376	0.64092
C	-6.77599	-1.57741	0.34136
C	-4.63675	-2.25461	-0.09835
C	-5.20876	0.08365	0.23960
C	-3.27524	1.74555	0.12298
C	-2.17756	-2.75479	-0.47472
C	-2.92023	-0.55047	-0.21049
N	-5.91450	-2.55365	0.08881
N	-6.46540	-0.28581	0.41710
N	-4.23600	-0.90451	-0.01950
B	-3.58765	-3.33819	-0.37010
N	-2.51116	0.72478	-0.16619
B	-4.78613	1.55925	0.32385
N	-1.97074	-1.46428	-0.44543
N	-2.67992	3.00058	0.18513
C	-1.32879	3.27931	0.20220
C	0.77919	2.54601	0.53191
C	0.30078	4.71413	-0.44630
C	2.07046	5.96508	-1.09191
C	2.93568	1.79592	0.48708
C	2.60338	3.88880	-0.34697
N	-0.51954	2.37217	0.72347
N	-0.99376	4.46802	-0.30615
N	1.22273	3.71541	-0.10406
N	1.65565	1.63996	0.89634
N	3.00346	5.06447	-0.85457
N	0.75223	5.86407	-0.93068
N	3.43840	2.92078	-0.08074
H	4.73575	1.00417	0.41752
H	-3.27236	3.80681	0.01798
H	-1.21240	-4.51074	-1.00794
N	4.46753	-1.46900	0.93486
H	-7.82446	-1.85351	0.49342
H	5.35551	-5.29831	-0.97454
H	2.42483	6.92811	-1.48269
N	2.21887	-0.91186	1.22866
N	0.54072	-2.23540	0.36739
N	1.12612	-4.22316	-0.81727
H	1.49563	-0.22241	1.47341
H	5.43105	-1.45489	1.25927
H	-5.59499	2.43310	0.51764
H	-3.88946	-4.50221	-0.47263

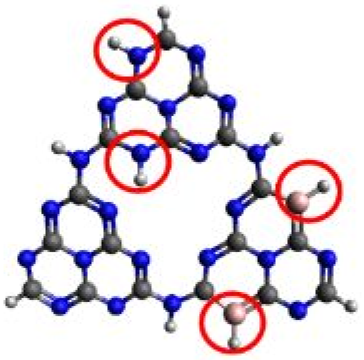

19

C -2.96875 -0.22515 -0.22183
C -2.58816 -2.54226 -0.16549
C -4.95143 -1.59087 0.02611
N -4.33228 -0.32559 -0.04251
N -6.25981 -1.66276 0.18409
C -5.13615 0.83225 0.06030
N -6.44737 0.68635 0.22003
C -2.96488 2.11853 -0.15620
C -6.96026 -0.53150 0.27655
N -1.66095 -3.56849 -0.15117
N -2.18159 3.28320 -0.18722
C 3.80996 -5.43847 -0.56545
C 1.72559 -4.55639 -0.30803
C 3.73304 -3.09259 -0.00349
C 3.48211 -0.91689 0.52483
C -0.28466 -3.51572 -0.02209
C 1.51692 -2.26564 0.45387
N 2.50432 -5.58228 -0.58326
N 4.43494 -4.22299 -0.29063
N 2.33369 -3.28835 0.02989
N 0.38291 -4.58795 -0.36487
N 2.14000 -1.12654 0.82915
N 4.28161 -1.92882 0.18490
N 0.23366 -2.35833 0.48934
N 3.96312 0.38477 0.49099
C 3.24725 1.53844 0.46065
C 1.16558 2.47205 0.38991
C 3.18916 3.75126 -0.02789
C 2.90058 5.94404 -0.52563
C -0.83322 3.42058 -0.03313
C 0.97511 4.77415 -0.30695
N 1.92993 1.44047 0.68256
N 3.91284 2.66961 0.17927
N 1.77951 3.66692 0.00277
N -0.14417 2.38156 0.42347
N 1.57429 5.93520 -0.56513
N 3.73267 4.93945 -0.29003
N -0.33674 4.62814 -0.34826
H -2.66759 4.15445 -0.37216
H 4.95669 0.47547 0.29975
H -2.01625 -4.50513 -0.31075
B -4.48349 2.21090 0.00121

H -5.13210 3.22724 0.07097
H -4.58146 -3.95899 -0.03935
H 1.56322 -0.27822 0.95801
H 5.44273 -4.13621 -0.33845
H 4.47240 -6.27861 -0.78084
H -8.04383 -0.62131 0.40615
H 3.37378 6.91490 -0.72190
N -2.36132 0.96687 -0.30326
N -2.17441 -1.30357 -0.30724
B -4.08618 -2.85816 -0.05874

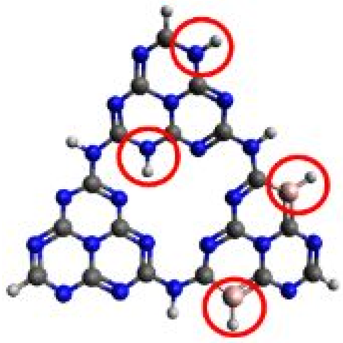

20

C -1.50535 -2.26105 0.36817
C 0.34144 -3.46713 -0.03003
C -1.64851 -4.51515 -0.24859
N -2.28597 -3.33643 0.04049
N -2.44413 -5.56180 -0.47354
C -3.80190 -3.20659 0.01689
N -4.46432 -4.31365 -0.23269
C -3.50561 -0.97591 0.43138
C -3.86918 -5.44881 -0.45211
N 1.69777 -3.53618 -0.17687
N -3.99046 0.33108 0.42754
C 6.97280 -0.43176 0.28767
C 4.97736 -1.52426 0.05719
C 5.13166 0.90091 0.04455
C 2.92718 2.16944 -0.15959
C 2.64159 -2.49751 -0.15678
C 2.98311 -0.17768 -0.21996
N 6.29230 -1.56835 0.21962
N 6.43883 0.78371 0.20634
N 4.34522 -0.26696 -0.03881
B 4.13290 -2.80127 -0.00869
N 2.35081 1.00320 -0.30063
B 4.45257 2.27668 -0.03152
N 2.21207 -1.27006 -0.30076
N 2.13477 3.31622 -0.16610
C 0.77099 3.43129 -0.03007
C -1.21682 2.45063 0.36241
C -1.04854 4.77276 -0.27856
C -2.98638 5.92709 -0.45803

C -3.29548 1.49020 0.42318
C -3.25604 3.71969 -0.00382
N 0.09993 2.37233 0.39104
N 0.26948 4.63799 -0.32598
N -1.83917 3.65175 0.01111
N -1.96451 1.40794 0.62544
N -3.80724 4.91690 -0.23357
N -1.65491 5.93053 -0.51084
N -3.96776 2.63420 0.18443
H -4.99025 0.40989 0.26227
H 2.60787 4.20080 -0.31734
H 2.05581 -4.47179 -0.33371
N -4.28781 -1.98794 0.16990
H 8.05773 -0.49809 0.41904
H -4.41674 -6.37002 -0.65263
H -3.46608 6.89927 -0.63133
N -2.12408 -1.12717 0.64242
N -0.19144 -2.32743 0.38318
N -0.33480 -4.60398 -0.31130
H -1.57976 -0.24791 0.76778
H 5.09567 3.29709 0.00837
H -1.99404 -6.43773 -0.71014
H 4.63655 -3.89702 0.05243

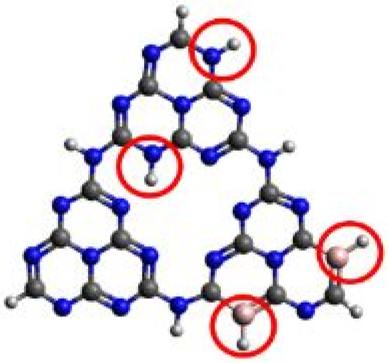

21

C 1.11731 2.44253 0.40602
C -0.87572 3.36396 -0.04325
C 0.94030 4.67974 -0.26314
N 1.73584 3.60904 0.05393
N 1.57711 5.82563 -0.49651
C 3.25602 3.69718 0.03278
N 3.75330 4.88506 -0.22855
C 3.27800 1.45059 0.46975
C 3.00375 5.92102 -0.46341
N -2.22568 3.20320 -0.22522
N 3.94077 0.22695 0.46008
C -6.98871 -0.51146 0.41461
C -4.89627 0.92001 0.08176
C -4.90651 -1.52883 0.05462
C -2.57636 -2.56915 -0.23193
C -2.91927 1.99550 -0.22547
C -2.85888 -0.27298 -0.34835

B -6.42551 0.89040 0.33231
N -6.22936 -1.58223 0.28013
N -4.23376 -0.29429 -0.05309
N -4.22016 2.06507 0.02224
N -2.10628 -1.37169 -0.46338
B -4.11270 -2.80470 -0.06069
N -2.25150 0.88674 -0.49191
N -1.68600 -3.61145 -0.14966
C -0.30233 -3.54011 -0.01408
C 1.51329 -2.27967 0.40212
C 1.68936 -4.59069 -0.28840
C 3.77529 -5.44344 -0.47979
C 3.42325 -1.02051 0.45815
C 3.71454 -3.22627 0.00497
N 0.19826 -2.41016 0.44723
N 0.36073 -4.64829 -0.34265
N 2.30256 -3.36949 0.02170
N 2.09436 -1.13913 0.67223
N 4.43614 -4.32766 -0.24082
N 2.45569 -5.64086 -0.53649
N 4.25668 -2.05151 0.20742
H 4.94024 0.29257 0.28501
H -2.05581 -4.55405 -0.22482
H -2.78202 4.04469 -0.33282
N 3.90715 2.56077 0.19438
H -8.05044 -0.73661 0.59036
H 3.41373 6.90925 -0.67246
H 4.39152 -6.33279 -0.66453
N 1.88764 1.40962 0.69764
N -0.19429 2.33090 0.42767
N -0.37451 4.57374 -0.34982
H 1.47063 0.46602 0.83049
H -4.62041 -3.89879 -0.00306
H 1.00783 6.62228 -0.75716
H -7.03929 1.92471 0.44315

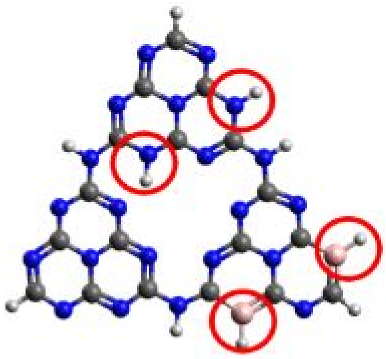

22

C -0.93395 2.81953 -1.17488
C 1.14448 3.26846 -0.29987
C -0.66200 4.65236 0.46343
N -1.48104 3.77822 -0.14789

N -1.11393 5.63344 1.22378
C -2.84721 3.84076 0.07054
N -3.32954 4.88580 0.76664
C -3.02704 1.73310 -0.73938
C -2.44911 5.71317 1.28542
N 2.50318 3.01206 -0.05466
N -3.83662 0.59937 -0.68420
C 6.92439 -1.12158 -0.19581
C 4.94614 0.50151 -0.05474
C 4.74015 -1.93820 0.07616
C 2.32734 -2.74506 0.29153
C 3.05355 1.76866 0.06365
C 2.79230 -0.47686 0.29442
B 6.48323 0.32497 -0.20609
N 6.06452 -2.11340 -0.06717
N 4.16994 -0.64228 0.09542
N 4.38047 1.69594 -0.08873
N 1.94183 -1.51167 0.42957
B 3.83600 -3.12984 0.19096
N 2.26740 0.72690 0.31904
N 1.34187 -3.72352 0.25674
C -0.00430 -3.54385 0.05427
C -1.68110 -2.13722 -0.47180
C -2.09579 -4.36874 0.35383
C -4.25787 -4.98349 0.61280
C -3.43557 -0.69989 -0.58889
C -3.95637 -2.81709 0.02644
N -0.39733 -2.40040 -0.49568
N -0.78818 -4.56245 0.41715
N -2.58048 -3.10512 -0.01793
N -2.15079 -0.95232 -0.83176
N -4.79330 -3.80097 0.34092
N -2.97514 -5.31907 0.65477
N -4.36576 -1.58637 -0.22470
H -4.80065 0.79516 -0.42822
H 1.62336 -4.68031 0.44728
H 3.18360 3.72813 -0.30024
N -3.63923 2.87581 -0.35747
H 7.97002 -1.44606 -0.29700
H -2.84958 6.56991 1.84164
H -4.96883 -5.78738 0.84146
N -1.77260 1.65901 -1.08608
N 0.37556 2.50932 -0.95591
N 0.67114 4.51406 0.23843
H -1.35949 0.73134 -1.24466
H 4.23694 -4.26939 0.19387
H 1.26540 5.08567 0.82705
H 7.19085 1.29727 -0.32033
